# Heat shock protein 90 inhibition attenuates inflammation in models of atopic dermatitis: a novel mechanism of action

**DOI:** 10.3389/fimmu.2023.1289788

**Published:** 2024-01-11

**Authors:** Hakim Ben Abdallah, Anne Bregnhøj, Gautam Ghatnekar, Lars Iversen, Claus Johansen

**Affiliations:** ^1^ Department of Dermatology and Venereology, Aarhus University Hospital, Aarhus, Denmark; ^2^ Regranion, Mount Pleasant, SC, United States

**Keywords:** Hsp90, atopic dermatitis, RGRN-305, small molecule, keratinocytes, MC903 mouse model

## Abstract

**Background:**

Heat shock protein 90 (HSP90) is an important chaperone supporting the function of many proinflammatory client proteins. Recent studies indicate HSP90 inhibition may be a novel mechanism of action for inflammatory skin diseases; however, this has not been explored in atopic dermatitis (AD).

**Objectives:**

Our study aimed to investigate HSP90 as a novel target to treat AD.

**Methods:**

Experimental models of AD were used including primary human keratinocytes stimulated with cytokines (TNF/IFNγ or TNF/IL-4) and a mouse model established by MC903 applications.

**Results:**

In primary human keratinocytes using RT-qPCR, the HSP90 inhibitor RGRN-305 strongly suppressed the gene expression of Th1- (*TNF*, *IL1B*, *IL6*) and Th2-associated (*CCL17*, *CCL22, TSLP)* cytokines and chemokines related to AD. We next demonstrated that topical and oral RGRN-305 robustly suppressed MC903-induced AD-like inflammation in mice by reducing clinical signs of dermatitis (oedema and erythema) and immune cell infiltration into the skin (T cells, neutrophils, mast cells). Interestingly, topical RGRN-305 exhibited similar or slightly inferior efficacy but less weight loss compared with topical dexamethasone. Furthermore, RNA sequencing of skin biopsies revealed that RGRN-305 attenuated MC903-induced transcriptome alterations, suppressing genes implicated in inflammation including AD-associated cytokines (*Il1b, Il4, Il6, Il13*), which was confirmed by RT-qPCR. Lastly, we discovered using Western blot that RGRN-305 disrupted JAK-STAT signaling by suppressing the activity of STAT3 and STAT6 in primary human keratinocytes, which was consistent with enrichment analyses from the mouse model.

**Conclusion:**

HSP90 inhibition by RGRN-305 robustly suppressed inflammation in experimental models mimicking AD, proving that HSP90 inhibition may be a novel mechanism of action in treating AD.

## Introduction

Atopic dermatitis (AD) is a common inflammatory skin disorder characterised by erythematous, scaly and itchy skin that imposes a high disease burden on patients and the healthcare system, affecting up to 20% of children and up to 5% of adults (1–3). AD is associated with atopic comorbidities (e.g., food allergy, allergic rhinitis and asthma), but other comorbidities have been reported including cardiovascular and neuropsychiatric disorders (4, 5). Although the multifactorial pathophysiology remains to be fully explored, the mechanisms underlying AD implicate a complex interaction of components involving skin barrier dysfunction, immune dysregulation, altered skin microbiome and genetic predisposition (6). Recent molecular insights into the disease mechanisms have revealed significant pathways and novel targets, enabling the vast ongoing drug development and the recent approval of therapeutics targeting IL-4, IL-13 and JAK1/2 (7). Nonetheless, some groups of patients do not achieve satisfactory long-term control or tolerate current treatments, highlighting the pressing need for novel treatments.

Heat shock protein 90 (HSP90) is a common chaperone that folds and supports the activity of client proteins including proteins involved in inflammation. Hence, HSP90 inhibition may be a wide-ranging approach targeting different inflammatory pathways, representing a novel mechanism of action for treating inflammatory skin diseases. In accordance, HSP90 inhibition demonstrated significant alleviation of skin inflammation in preclinical studies of psoriasis, irritative contact dermatitis and epidermolysis bullosa acquisita (8–11). The anti-inflammatory effects of HSP90 inhibition have also been demonstrated in several experimental models beyond dermatology including rheumatoid arthritis, systemic sclerosis, systemic lupus erythematous, encephalomyelitis and colitis (12–17). This suggests that HSP90 plays an important role in modulating inflammation. Recently, two recent proof-of-concept studies revealed that orally administered HSP90 inhibitor (RGRN-305) led to marked improvements in psoriasis (open-label study; n=13) and hidradenitis suppurativa (randomised controlled trial; n=15) (18, 19). Another recent study revealed that HSP90 serum levels were 2.79-fold increased (P > 0.001) in AD patients (n = 27) compared with healthy controls (n = 70) and correlated with disease severity (20). Yet, to the best of our knowledge, no studies have explored the effects of HSP90 inhibition in atopic dermatitis. Therefore, our study aimed to investigate HSP90 as a novel target for treating atopic dermatitis using experimental models.

## Materials and methods

### HSP90 inhibitor

Regranion (Mount Pleasant, SC, USA) kindly provided the HSP90 inhibitor RGRN-305 (MW = 443 g/mol), formerly named Debio 0932/CUDC-305, which inhibits HSP90α/β (IC50 ~ 0.1 μM).

### Cell culture and experiments

Primary normal human epidermal keratinocytes (NHEKs) were isolated from nine healthy donors as described previously (21). The keratinocytes were plated in 6-well plates and cultured in keratinocyte SFM (Gibco, ThermoFisher Scientific, Waltham, MA, USA) supplemented with growth factors and 5 µg/ml Gentamicin (Gibco) at 37°C in a humidified incubator containing 5% CO_2_ until 60-70% confluency. Subsequently, the keratinocytes were starved for 24 hours in keratinocyte SFM medium without growth factors before the initiation of experiments. After 2 hours of preincubation with vehicle (0.2% ethanol diluted in water), RGRN-305 (5 µM) or dexamethasone (0.1 µM; Sigma-Aldrich, Burlington, MA, USA), the keratinocytes were stimulated with TNF (10 ng/mL; PeproTech, London, UK) in combination with IFNγ (10 ng/mL; PeproTech) or IL-4 (50 ng/mL; PeproTech) for up to 24 hours to induce an AD-like phenotype (22–24). The used concentration of RGRN-305 was based on previous concentration studies demonstrating anti-inflammatory properties and low toxicity in stimulated NHEKs (8, 10).

### Human samples

Four-millimetres punch biopsies from six AD patients were collected from lesional and non-lesional skin and stored in liquid nitrogen until RNA isolation.

### RNA isolation from NHEKs

Keratinocytes were washed with phosphate-buffered saline (PBS; Gibco) followed by RNA extraction using SV 96 Total RNA Isolation System (Promega, Madison, WI, USA) per the manufacturers’ instructions. NanoDrop 2000 Spectrophotometer (ThermoFisher) was used to determine the RNA concentration and purity.

### RNA isolation from punch biopsies

Punch biopsies were stored in 750 µL RNAlater-ICE (ThermoFisher) at -80°C for 20 minutes and overnight at -20°C. Then, the biopsies were added to 175 µL SV RNA lysis buffer with β-mercaptoethanol (SV Total RNA Isolation System; Promega) followed by homogenization using TissueLyser (Qiagen, Hilden, Germany). The remaining steps including RNA purification and DNase treatment were conducted following the manufacturer’s instructions.

### Reverse transcription-quantitative PCR

cDNA was generated using total RNA, TaqMan Reverse Transcription Reagents with random hexamers (ThermoFisher) and Peltier Thermal Cycler-200 (MJ Research Inc, Waltham, MA, USA) following the manufacturer’s instructions. Real-time PCR was carried out with 20 ng cDNA per 20 µL reaction in StepOnePlus Real-Time PCR system (ThemoFisher) using TaqMan Universal PCR Master Mix, primers and probes (ThermoFisher) for *TNF* (Hs00174128_m1), *IL1B* (Hs01555410_m1), *IL6* (Hs00174131_m1), *TSLP* (Hs00263639_m1), *CCL17* (Hs00171074_m1), *CCL22* (Hs01574247_m1), *HSPA1A* (Hs00359163_s1), *HSPB1* (Hs00356629_g1), *Il1b* (Mm00434228_m1), *Il4* (Mm00445259_m1), *Il6* (Mm00446190_m1), *Il13* (Mm00434204_m1), *RPLP0* (Hs99999902_m1) and *Gapdh* (Mm99999915_m1). Three technical replicates for each sample underwent real-time PCR; 2 minutes at 50°C and 10 minutes at 95°C followed by 40 cycles of 15 seconds at 95°C and 1 minute at 60°C. The standard curve method with StepOne Software v2.1 and reference genes (*RPLP0* and *Gapdh)* were used to obtain normalised relative expression levels of target genes (25).

### Protein isolation from NHEKs

PBS-washed keratinocytes were added to cell lysis buffer with cOmplete Protease Inhibitor Cocktail and phenylmethylsulphonyl fluoride (Sigma-Aldrich). The lysate was centrifuged at 13,000 g for 3 minutes followed by collection of the supernatant containing the protein extract. The protein concentration was determined by Bradford Protein Assay.

### Western blot

A total of 20 µg protein extract for each sample was loaded on 10% mini-PROTEAN TGX PreCast Gel (Bio-Rad, Hercules, CA, USA) and separated by gel electrophoresis using Mini Trans-Blot Cell (Bio-Rad). The subsequent protein blotting onto nitrocellulose membranes was performed with Trans-Blot Turbo Transfer System (Bio-Rad). Membranes were incubated overnight at 4°C with the following primary antibodies from Cell Signaling Technology Danvers, MA, USA (catalog#): P-STAT1 (#9167), P-STAT3 (#9145) P-p65 (#3033), and P-STAT6 (#56554). After washing, the membranes were incubated with HRP-conjugated anti-rabbit IgG antibody (Cell Signaling Technology; catalog#7074) at room temperature for 1 hour. Protein bands were detected with an enhanced chemiluminescence (ECL) reaction using Clarity Western ECL Substrate (Bio-Rad) and digitally imaged with C-DiGit Blot Scanner (LI-COR, Lincoln, NE, USA). To normalize the protein expression to β-actin levels, the membranes were stripped and reprobed with an anti-β-actin antibody (A1978; Sigma-Aldrich), which was detected by HRP-conjugated anti-mouse IgG (p0447; Dako, Glostrup, Denmark). Relative intensities of bands were quantified by densitometric analyses using Image Studio Digits Version 3.1 (LI-COR).

### Mice

Female Balb/cAnNRj (8 weeks old) mice were purchased from Janvier Labs (Le Genest-Saint-Isle, France), and housed in animal facilities at 19-25°C with 12-hour light/dark cycles and free access to laboratory rodent diet and water. The mice had at least 1 week acclimation period before the experiments started.

### Atopic dermatitis mouse model

Mice were randomly divided to receive: vehicle and drug-vehicle (negative control); MC903 (MedChemExpress, Monmouth Junction, NJ, USA) and drug-vehicle (disease control); MC903 and topical or oral RGRN-305 (RGRN-305 treatment groups); or MC903 and topical dexamethasone (positive control; purchased from Sigma-Aldrich). Four to eight mice were allocated to each group, totalling 76 mice for all experiments.

To induce AD-like skin inflammation, 1 or 2.5 nmol of MC903 in 25 µL absolute ethanol was topically applied once daily to the ventral and dorsal surface of the right ear for six consecutive days. After MC903 challenge, the mice were treated with topical RGRN-305 (1 mg in 25 µL ethanol), oral RGRN-305 (100 mg/kg), topical dexamethasone (20 nmol in 25 µl ethanol), or drug-vehicle (topical ethanol or oral 5% kleptose [HPB, Roquette Pharma, Lestrem, France]). The topical treatments were applied 2 hours post-MC903 challenge, whereas the oral treatments were administered by oral gavage immediately post-MC903 challenge. At the end of the experiment on day 9, clinical photos of the ears were taken and the mice were euthanised by cervical dislocation. Two punch biopsies (4 mm) from the ear were collected for RNA isolation and histological assessment. Ear thickness, the primary clinical endpoint for inflammation, was measured daily with a digital caliper (Mitutoyo Corporation, Kawasaki, Japan), and body weight was measured throughout the study to monitor the welfare of mice. During the experimental procedures, the mice were anesthetized with 2% isoflurane.

### Histology

Four-millimetres punch biopsies from the ear were fixed in 4% formaldehyde overnight at 4°C, paraffin-embedded, and serially sliced into 4 µm sections subjected to staining procedures. For haematoxylin & eosin (HE) staining, a standard protocol was followed. To detect mast cells, sections were stained with 0.1% toluidine blue solution (Sigma-Aldrich) at pH 2.3 and room temperature for 3 minutes. For immunohistochemical staining, heat-induced antigen retrieval was performed (25 minutes at 97**°** C) in Tris-EGTA buffer (pH 9), and stained with anti-CD4 antibody (1:1000; EPR19514, Abcam, Cambridge, UK) or anti-Ly6g antibody (1:2000; EPR22909-135, Abcam) for 1 hour at room temperature. The remaining steps for detection were performed with Ultravision Quanto Detection System (ThermoFisher) following the manufacturer’s instructions. The sections were counterstained with hematoxylin.

All slides were scanned with 20× objective using the whole slide scanner NanoZoomer 2.0-HT (Hamamatsu Photonics K.K, Hamamatsu City, Japan). Quantitative image analysis was performed in QuPath 0.4.2 using the *Cell Detection* command with default settings followed by a manual review and corrections (26). The number of counted cells was normalised to the length of the section

### RNA sequencing

Paired-end RNA sequencing of total RNA was conducted by Eurofins Genomics Europe Sequencing GmbH (Konstanz, Germany) in accordance with their protocols. To prepare the RNA library, NEBNext Ultra II Directional RNA Library Prep Kit for Illumina was used with 100 ng of total RNA. The mRNA quality was assessed by Fragment Analyzer. Illumina NovaSeq 6000 platform in 2x150 Sequence mode was performed to acquire at least 20 million read pairs. Raw sequence data quality was assessed using FastQC (version 0.12.0). Read alignment to the mouse reference genome (GRCm39 primary assembly) and counting of uniquely aligned unambiguous reads were performed with the R package Subread (version 2.10.3) (27). Regularized logarithm transformation (rlog) performed with the R package DESeq2 (1.36.0) was used for library size correction and variance stabilization (28). The rlog normalized counts were used for principal component analysis (PCA) and heat map analysis (pheatmap 1.0.12). DESeq2 with independent filtering and Wald test enabled were used for the differential gene expression analyses (28). To adjust for multiple comparisons, the p-values were adjusted by setting the false discovery rate (FDR) at 0.05 with the Benjamini and Hochberg procedure. Kyoto Encyclopaedia of Genes and Genomes (KEGG) and Gene Ontology (GO) enrichment analyses were generated with DAVID and validated with ShinyGO 0.76 (29, 30).

### Statistical analysis

The statistical significance threshold was set at P < 0.05. Differences between treatment groups in clinical endpoints, mRNA (qPCR) and protein levels (Western blot) were analysed by unpaired t-tests for mice and paired t-tests for keratinocytes. If data were not normally distributed, Mann-Whitney and Wilcoxon signed-rank tests were performed. All statistical analyses were generated with GraphPad Prism 9.0 and R 4.2.0 software.

### Ethical statement

The experiments with NHEKs were approved by the Central Jutland Regional Committee on Health Research Ethics (M-20110027), and informed consent was obtained from the donors. Permission to obtain punch biopsies from patients with atopic dermatitis was approved by Central Jutland Regional Committee on Health Research Ethics (M-20090102).

The animal experiments were approved by The Danish Animal Experiments Inspectorate (2022-15-0201-01267) and carried out in agreement with the Danish Animal Welfare Act for the Care and Use of Animals for Scientific Purposes. The animal experiments conducted at Comparative Biosciences were approved by their institutional review board. All efforts were made to minimize the distress experienced by the laboratory animals.

## Results

### RGRN-305 robustly inhibited AD-associated cytokine expression in NHEKs

**Figure 1:**
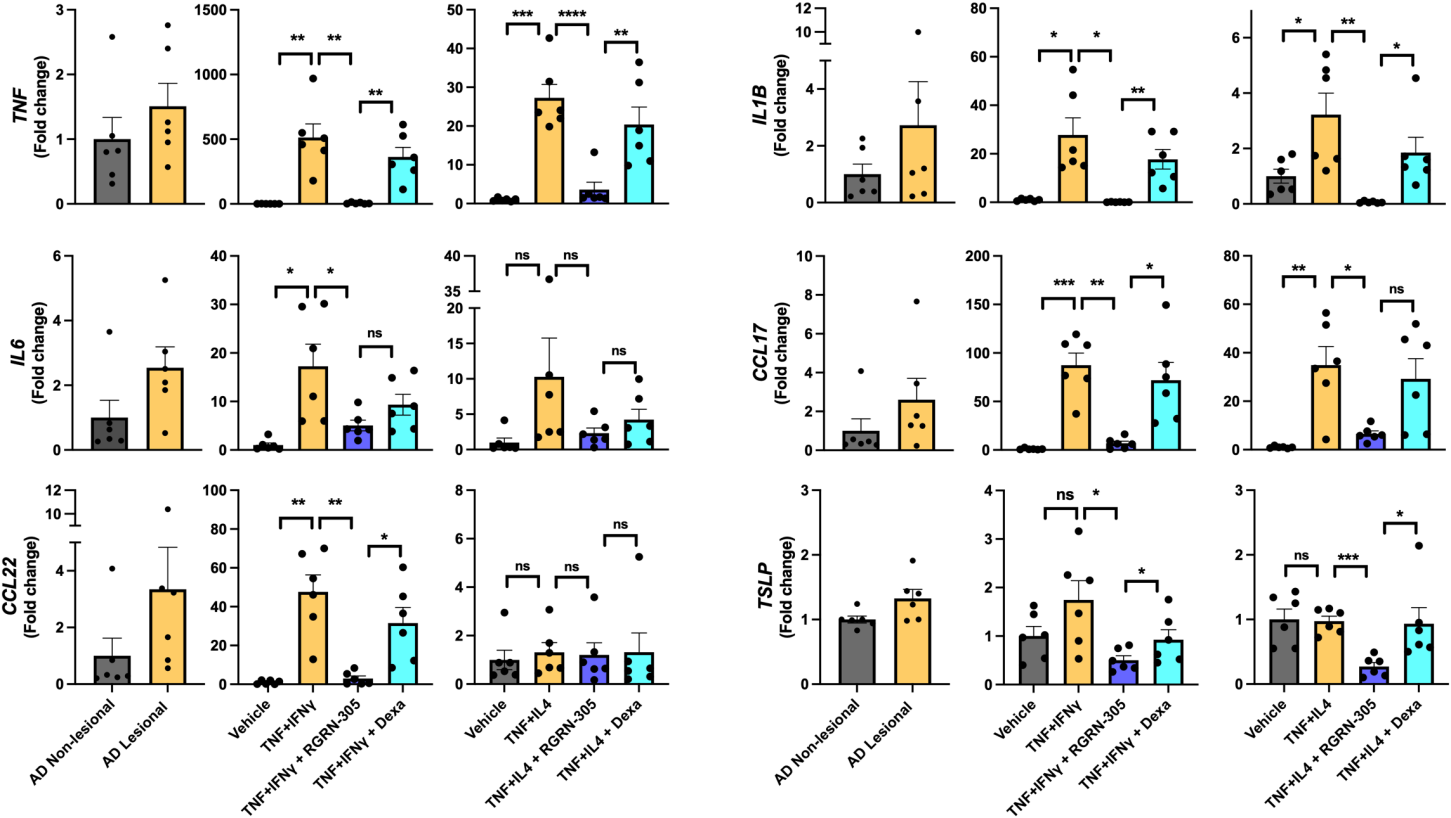
RT-qPCR analysis of AD-related gene expression in lesional/non-lesional AD skin and cytokine-stimulated primary human keratinocytes. Effects of RGRN-305 (5 µM) or dexamethasone (0.1 µM) on inflammatory gene expression in primary human keratinocytes stimulated with TNF (10 ng/ml) in combination with IFNγ (10 ng/ml) or IL-4 (100 ng/mL) for 24 hours (three independent experiments, n = 6). Data are shown as mean ± SEM. *p < 0.05, **p ≤0.01, ***p ≤0.001, ****p ≤ 0.0001. ns, not significant. AD, atopic dermatitis. Dexa, dexamethasone.

To explore the anti-inflammatory effects of HSP90 inhibition in atopic dermatitis models *in vitro*, NHEKs from healthy donors were stimulated with TNF/IFNγ or TNF/IL-4, as experimental models, to mimic an AD-related gene expression. qPCR analysis demonstrated that inflammatory cytokines (*TNF*, *IL1B*, *IL6*) and chemokines (*CCL17*, *CCL22*) were statistically significantly upregulated in these models, consistent with an observed increased expression in human AD lesional skin compared with non-lesional skin (**Figure 1**). Interestingly, RGRN-305 robustly inhibited the expression of these AD-associated cytokines and chemokines compared with stimulated/vehicle- and stimulated/dexamethasone-treated NHEKs. While not significantly upregulated in our models, RGRN-305 also suppressed the expression of *TSLP*, supporting a convincing anti-inflammatory effect mediated by HSP90 inhibition in keratinocytes. RGRN-305 caused low (2-5%) cytotoxicity in the stimulated primary human keratinocytes (**Supplementary Figure S1**).

### RGRN-305 suppresses the activity of STAT3 and STAT6 signalling pathways in NHEKs

**Figure 2:**
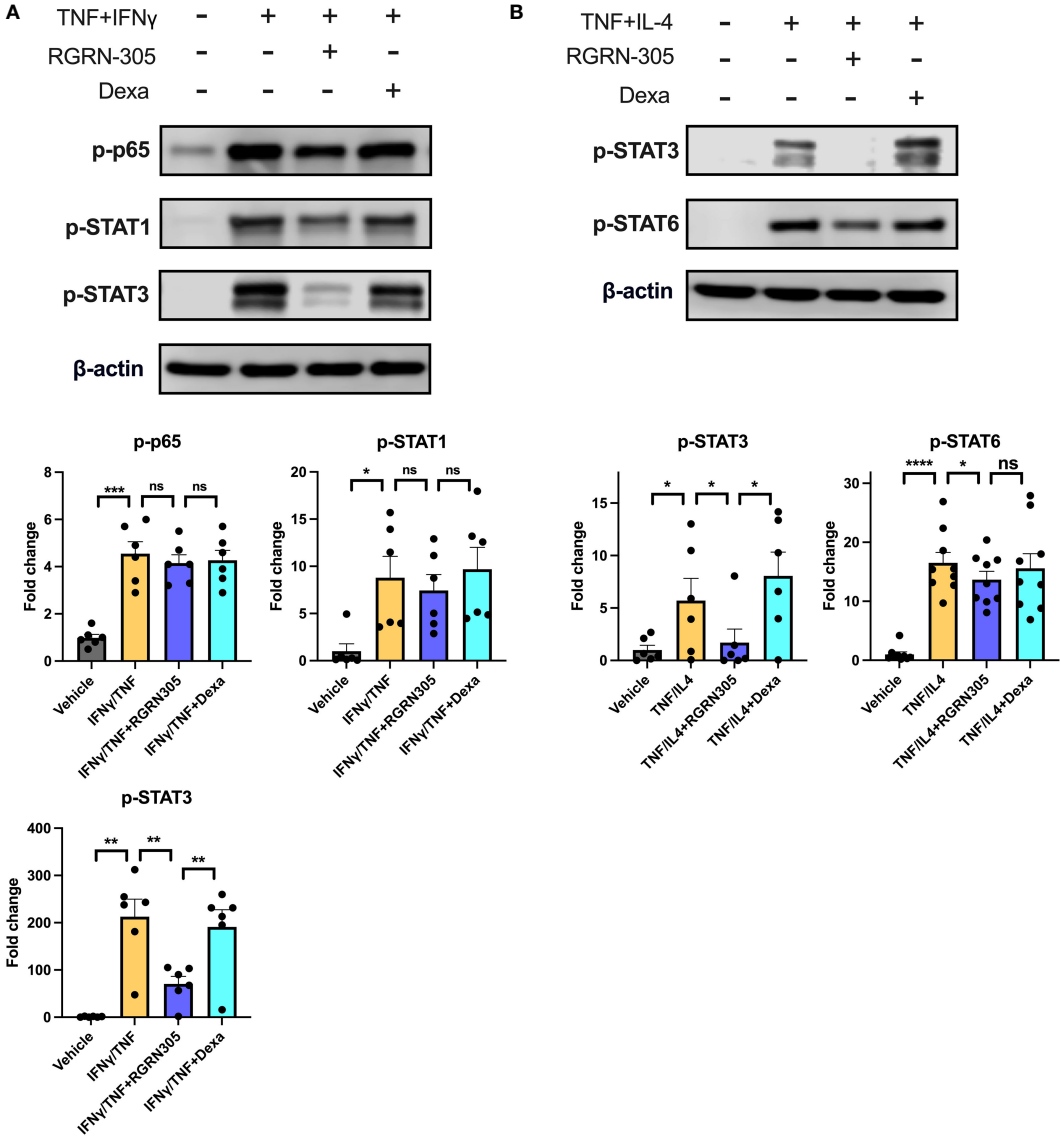
Effects of RGRN-305 (5 µM) or dexamethasone (0.1 µM) on the phosphorylation status of key signalling proteins determined by Western blot in primary human keratinocytes stimulated with TNF (10 ng/ml) in combination with **(A)** IFNγ (10 ng/ml) or **(B)** IL-4 (100 ng/mL) for 1 hour (three independent experiments, n = 6-9). Representative Western blots and densitometric results (mean ± SEM) are shown. *p < 0.05, **p ≤0.01, ***p ≤0.001, ****p ≤ 0.0001. ns, not significant. Dexa, dexamethasone.

To further elucidate the potential AD-related pathways targeted by HSP90 inhibition in keratinocytes, the phosphorylation status of key signalling proteins, including STAT1, STAT3, STAT6 and p65 (subunit of NF-κB), was determined by Western blot (**Figure 2**). Importantly, RGRN-305 significantly suppressed the phosphorylation of STAT3 under TNF/IFNγ or TNF/IL-4 stimulation, and to a lesser degree STAT6 (∼20% reduction) under TNF/IL-4 stimulation. However, RGRN-305 had no significant effects on the phosphorylation of STAT1 and p65.

### Topical RGRN-305 attenuates MC903-induced AD-like inflammation in mice

**Figure 3:**
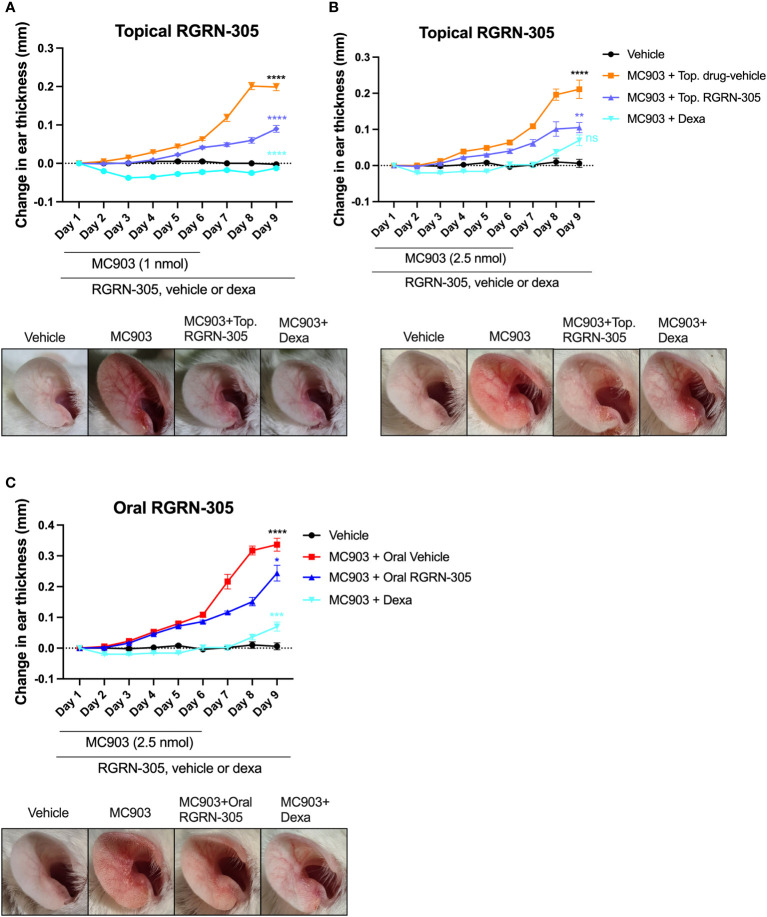
Ear thickness changes throughout the experiments and photographs on day 9 in MC903-induced atopic dermatitis mouse model. Female Balb/cAnNRj ears were challenged daily with **(A)** 1 nmol or **(B)** 2.5 nmol of MC903 for 6 days and treated once daily with topical drug-vehicle, topical dexamethasone (20 nmol) or topical RGRN-305 (1 mg). **(C)** Mice were challenged daily with 2.5 nmol for 6 days and treated once daily with oral drug-vehicle, topical dexamethasone (20 nmol) or oral RGRN-305 (100 mg/kg). Four to eight mice in each group, in total 76 mice. Two independent experiments were conducted. Data are shown as mean ± SEM. *p < 0.05, **p ≤0.01, ***p ≤0.001, ****p ≤ 0.0001. ns, not significant. Statistical significance denotes pair-wise comparison on day 9; Black, MC903+Drug-vehicle vs. Vehicle; Blue, MC903+RGRN-305 vs. MC903+Drug-vehicle; Teal, MC903+RGRN-305 vs. MC903+Dexa. Dexa, dexamethasone. Top, topical.

Next, we investigated the effects of HSP90 inhibition on AD-like skin inflammation in a mouse model for atopic dermatitis. The right ears of BALB/c mice were challenged daily with 1 nmol MC903 or vehicle for 6 days and received topical treatment with drug-vehicle (ethanol), RGRN-305 or dexamethasone throughout the experiment for 9 days. Treatment with RGRN-305 resulted in visibly reduced erythema and a highly significant reduction (55%) in ear thickness (a marker of inflammation) compared with drug-vehicle at the end of the experiment on day 9 (**Figure 3A**). Dexamethasone treatment was highly potent resulting in ear thickness below baseline levels and was more effective than RGRN-305. However, dexamethasone-treated mice lost significantly more weight than RGRN-305-treated mice, indicating a more favourable safety profile for RGRN-305 (**Supplementary Figure S2**).

Next, we repeated the experiment with 2.5 nmol (increased from 1 nmol) of MC903 challenge to observe whether RGRN-305 could suppress a stronger stimulus of inflammation (**Figure 3B**). Interestingly, topical RGRN-305 still significantly suppressed the MC903-induced ear thickening by 50% on day 9, which was comparable to dexamethasone treatment (P = 0.13).

### Oral RGRN-305 ameliorates MC903-induced AD-like inflammation in mice

To evaluate the feasibility of orally administered RGRN-305, mice were challenged with 2.5 nmol MC903 for six days and administered 100 mg/kg RGRN-305 by oral gavage once daily throughout the experiment. Oral RGRN-305 treatment led to visibly reduced erythema and significantly reduced ear thickening (28% reduction) compared with drug-vehicle, but RGRN-305 was less effective than dexamethasone (**Figure 3C**). Furthermore, an independent testing facility (Comparative Biosciences, Sunnyvale, CA, USA) demonstrated a dose-dependent improvement by oral RGRN-305 (20, 50 and 100 mg/kg) in alleviating AD-like inflammation in BALB/c mice using an alternative MC903 regimen, further supporting our findings (**Supplementary Figure S3**, **Supplementary Appendix S1**).

### Topical RGRN-305 decreases MC903-induced immune cell infiltration in mice

**Figure 4:**
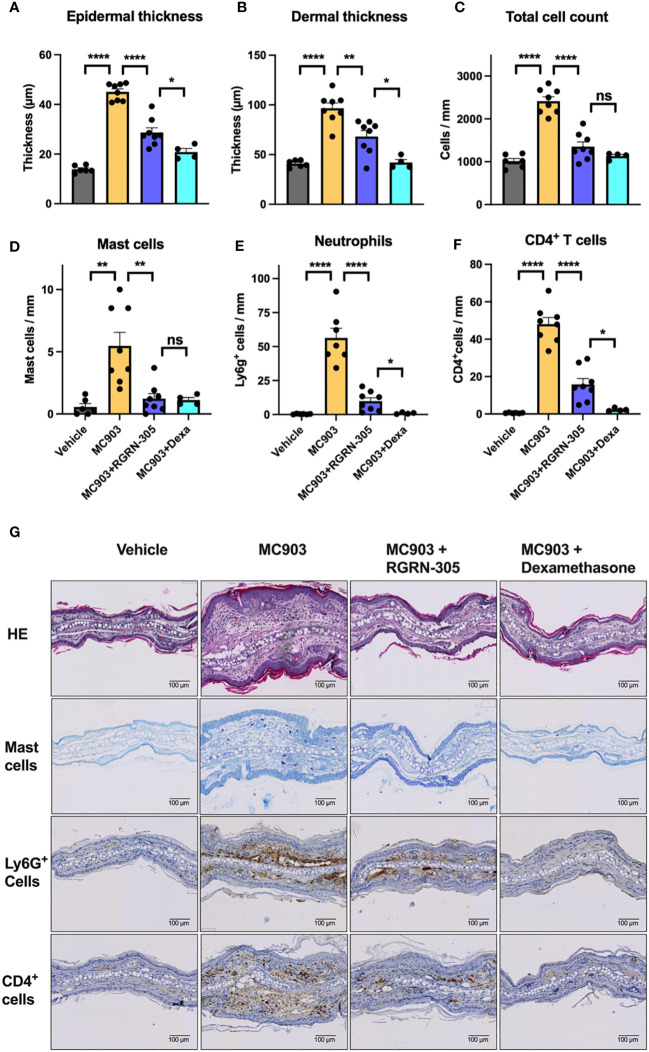
Histological analysis of ear biopsies from mice challenged daily with 1 nmol of MC903 and treated with drug-vehicle, topical RGRN-305 or topical dexamethasone. **(A)** Epidermal thickness, **(B)** dermal thickness and **(C)** total number of cells were determined from haematoxylin & eosin (HE) stained sections. **(D)** Quantification of mast cells in toluidine blue stained sections. **(E)** Quantification of Lyg6+ cells (neutrophils) and **(F)** CD4+ cells (T cells) in immunohistochemical stained sections. **(G)** Representative images of stained sections. Data are shown as mean ± SEM. *p < 0.05, **p ≤0.01, ****p ≤ 0.0001. ns, not significant. Dexa, dexamethasone.

To gain insight into the mechanisms of HSP90 inhibition, punch biopsies derived from the experiment with topical RGRN-305 and 1 nmol MC903 challenge were investigated. Histological analyses confirmed that MC903 increased the epidermal and dermal thickness, which was significantly decreased by RGRN-305 or dexamethasone (**Figures 4A, B**). Moreover, the total number of cells within histological sections was significantly reduced by RGRN-305 to levels broadly similar with dexamethasone (**Figure 4C**). In line with this, RGRN-305 significantly suppressed immune cell infiltration into the skin including mast cells, Ly6g^+^ cells (neutrophils) and CD4^+^ cells (T-cells; **Figures 4D-G**).

### Topical RGRN-305 inhibits MC903-induced transcriptome alterations and AD-associated cytokine expression in mice

**Figure 5:**
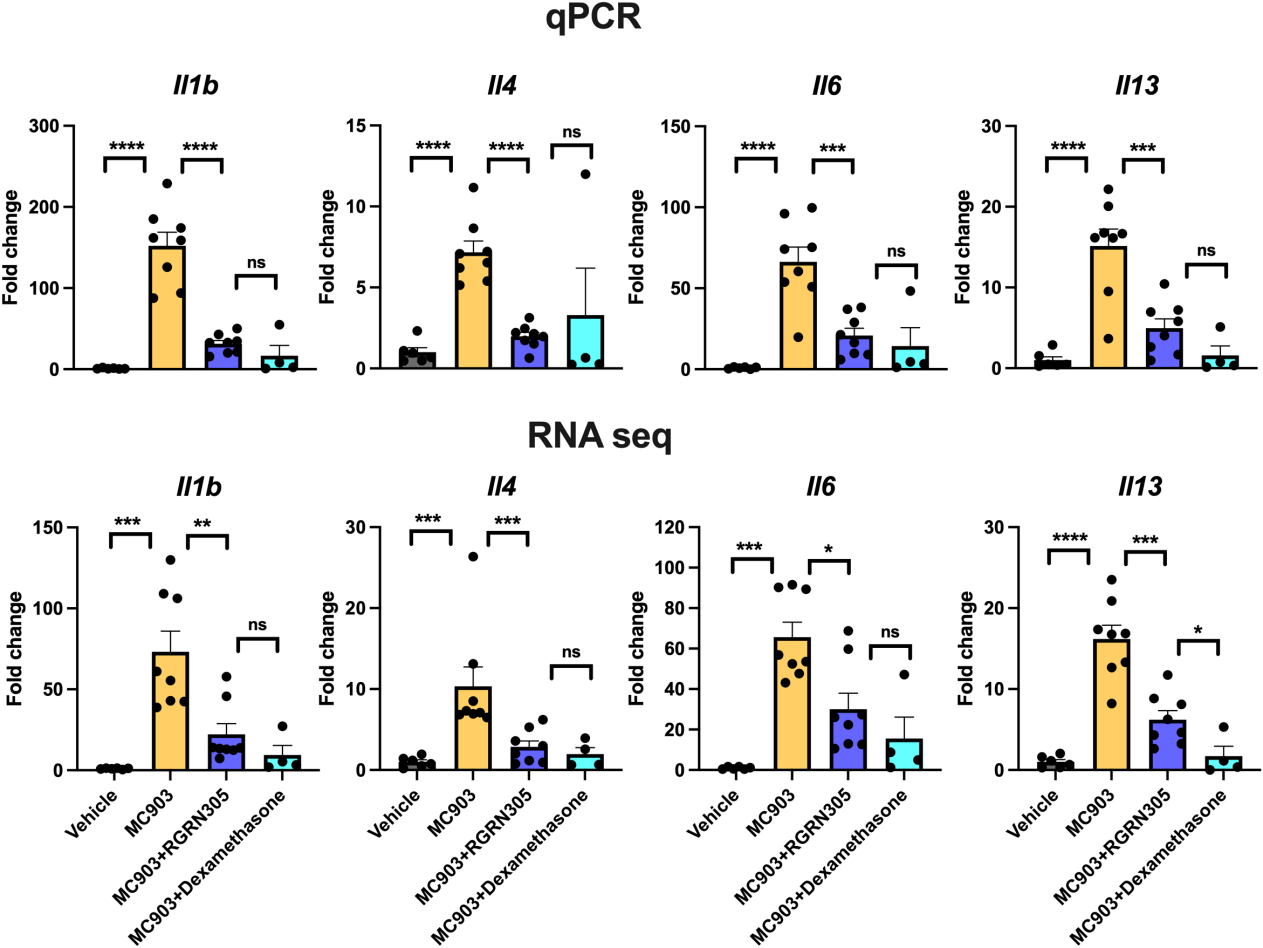
RT**-**qPCR (upper panels) and RNA sequencing analysis (lower panels) of AD-related cytokine gene expression in ear tissue from mice challenged daily with 1 nmol of MC903 and treated with drug-vehicle, topical RGRN-305 or topical dexamethasone. Data are shown as mean ± SEM. *p < 0.05, **p ≤0.01, ***p ≤0.001, ****p ≤ 0.0001. ns, not significant.

**Figure 6:**
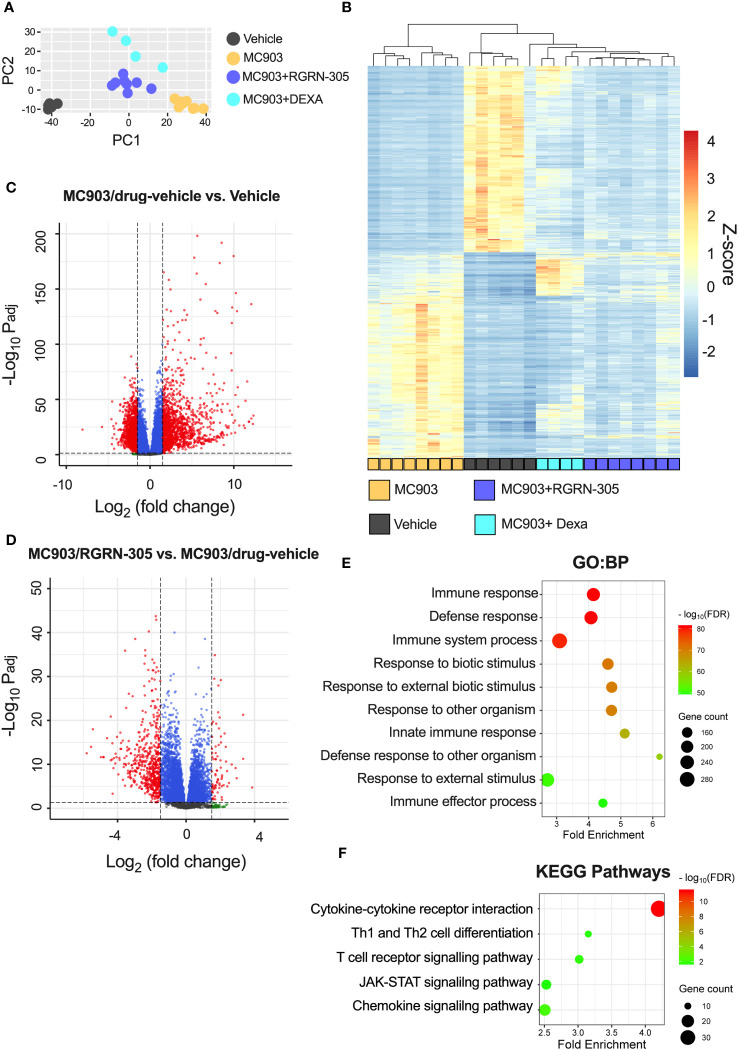
RNA sequencing analysis of ear tissue from mice challenged daily with 1 nmol of MC903 and treated with drug-vehicle, topical RGRN-305 or topical dexamethasone. **(A)** Principal component analysis (PCA) of regularized log (rlog) transformed data. **(B)** Heatmap of differentially expressed genes (DEGs; log_2_[foldchange] > 1.5 and FDR-adjusted p-value < 0.05) upon MC903-induced inflammation. The genes were filtered to include those with a mean of normalised counts > 25. **(C, D)** Volcano plots for two different comparisons. The dashed horizontal lines mark an FDR-adjusted p-value of 0.05, whereas the vertical lines mark a log_2_(fold change) of 1.5. DEGs are marked as red dots. **(E, F)** Enrichment analyses of down-regulated DEGs by RGRN-305 compared with drug-vehicle. **(E)** The top ten significant GO biological processes terms and **(F)** five relevant significantly enriched KEGG pathway terms are presented. Dexa, dexamethasone. PCA, Principal component analysis.

To examine alterations in gene expression, RNA sequencing and qPCR analyses of ear tissue were performed and showed that topical RGRN-305-treated mice exhibited significantly reduced AD-related cytokine expression of *Il1b*, *Il4*, *Il6* and *Il13* compared with drug-vehicle-treated mice (**Figure 5**). Using RNA sequencing data, a principal component analysis (PCA) and a hierarchically clustered heatmap revealed that the mice clustered into their respective treatment groups (**Figures 6A, B**). The MC903/drug-vehicle-treated mice exhibited a distinctive expression pattern separated from the RGRN-305-treated mice, indicating that RGRN-305 treatment mitigates MC903-induced transcriptome alternations. In addition, volcano plots illustrate that MC903 challenge skewed the trend of differentially expressed genes (DEGs) to the right, indicating upregulation of genes (**Figure 6C**); whereas, RGRN-305 treatment compared with drug-vehicle, skewed the trend of DEGs to the left, indicating down-regulation of genes induced by MC903 (**Figure 6D**). To explore the biological functions of the DEGs, enrichment analyses were performed for down-regulated DEGs by RGRN-305 compared with drug-vehicle. The analyses revealed that the top ten significant GO biological process (BP) terms were mostly related to inflammation, revealing that RGRN-305 suppressed genes involved in inflammation (**Figure 6E**). Furthermore, KEGG pathway enrichment analysis showed that RGRN-305 down-regulated genes implicated in inflammatory pathways including JAK-STAT signalling (**Figure 6F**). Lists of all genes derived from the differential expression analyses are present in **Supplementary Table S1**.

### RGRN-305 upregulates the gene expression of *HSPA1A/Hspa1a* (HSP70)

To investigate a potential compensatory heat shock response secondary to HSP90 inhibition by RGRN-305, the gene expression of *HSPA1A* (HSP70) and *HSPB1* (HSP27) was determined by qPCR and RNA sequencing (**Supplementary Figure S4**). In primary human keratinocytes, RGRN-305 treatment caused a trend towards upregulation of *HSPA1A* (*P* = 0.059), whereas no change was observed for *HSPB1*. In the MC903-mouse model, RGRN-305 treatment led to a highly significant upregulation of *Hspa1a* and *Hspb1.*

## Discussion

In this study, we discovered HSP90 inhibition by RGRN-305 robustly suppressed inflammation in experimental models of AD by significantly reducing clinical symptoms of dermatitis (erythema and oedema), immune cell infiltrations, expression of key cytokines (e.g., IL-4 and IL-13) and signalling pathways (STAT3 and STAT6) related to AD. Thus, our encouraging results suggest HSP90 may be considered a novel therapeutic target for AD, providing preclinical validation for further clinical investigations in patients.

While HSP90 inhibitors have been researched for oncological disorders, the therapeutic potential of HSP90 inhibitors in inflammation has been sparsely examined. To the best of our knowledge, two proof-of-concept clinical studies and a small number of preclinical studies have been conducted (18, 19, 31, 32). Our findings are in line with other preclinical studies beyond atopic dermatitis wherein HSP90 inhibition exerted anti-inflammatory effects by targeting several inflammatory cytokines (e.g. TNF, IL-1β, and IL-6) and signalling pathways (e.g. ERK1/2, p38, and JNKs) (8, 12, 14, 33).

Atopic dermatitis is routinely treated with emollients in combination with topical anti-inflammatory treatments such as corticosteroids or calcineurin inhibitors as first-line therapies, whereas systemic treatments are reserved for more severe or recalcitrant cases (34). We demonstrated that both topical and oral RGRN-305 significantly reduced AD-like skin inflammation in mice. While both routes of administration may be feasible in treating AD, topically delivered RGRN-305 may be the better option as the reduction of ear thickness was 50-54% compared with 28% for oral RGRN-305. Moreover, topical RGRN-305 demonstrated similar or slightly inferior efficacy compared with topical dexamethasone, a very potent corticosteroid (35). Yet, RGRN-305 treatment resulted in significantly less weight loss compared with dexamethasone, indicating a more favourable safety profile. In agreement, oral treatment with RGRN-305 has been well-tolerated in proof-of-concept studies with psoriasis and hidradenitis suppurative patients (18, 19). It is worth nothing that RGRN-305 has been shown to accumulate in the skin (9), thus additional time than this duration study investigated (nine days) may allow orally delivered RGRN-305 to build sufficient levels in the skin and thereby increase the efficacy. Considering that atopic dermatitis is treated frequently with topical corticosteroids, it may be relevant to consider whether treatment with RGRN-305 in combination with dexamethasone may enhance the immunosuppressive and clinical effects in AD, warranting further investigation.

In the acute phase of AD, a Th2 response is initiated and shifts towards a dominancy of Th1 response as the disease progresses into its chronic stage (36, 37). Activated keratinocytes, as key effector cells, play a crucial role in promoting immune dysregulation by secreting a variety of cytokines and chemokines (e.g. IL-1β, IL-6, CCL17, TSLP), contributing to the pathogenesis in AD (38). In primary human keratinocytes and mice, RGRN-305 treatment strongly suppressed Th1- and Th2-associated cytokines and chemokines implicated in AD (**Figures 1**, **5**). In human keratinocytes, RGRN-305 inhibited the phosphorylation (i.e., activation) of STAT3 and, to a lesser extent, STAT6 (**Figure 2**); two key signalling proteins downstream of the IL-4 receptor (39). In accordance, enrichment analysis from the mouse model showed JAK-STAT signalling was highly downregulated by RGRN-305. Interestingly, another recent study (n = 7) discovered that HSP90 inhibition disrupted JAK-STAT signalling by potently decreasing JAK2 expression and the phosphorylation of STAT3 and STAT5 in patients with myeloproliferative neoplasms (40). In addition, the relation between HSP90 and activation of STAT3, STAT5 and STAT6 have been reported in preclinical cancer studies, further supporting our findings (41–43). The recent success of therapeutics targeting JAK-STAT pathways highlights the importance of these pathways in AD, and it may be one of the mechanisms by which HSP90 inhibition ameliorates AD (44). However, considering the wide range of client proteins targeted by HSP90 inhibition, the anti-inflammatory effects are most likely attributable to other mechanisms as well.

While the pathophysiological mechanisms underlying AD are complex, it is noteworthy that inhibition of cell-surface HSP90 has been demonstrated to reduce Toll-like receptor (TLR) signalling in response to pathogen-associated molecular patterns (PAMPs) in human monocytes, suggesting that HSP90 promotes TLR signalling (45). This relationship may have implications for AD, as TLR signalling can influence various cell types with wide-ranging effects, potentially affecting vitamin D metabolism (46). Further research is warranted to investigate the significance of this potential mechanism in atopic dermatitis.

HSP90 inhibitors may trigger a heat shock response by dissociating heat shock factor 1 (HSF1) from HSP90, which may induce the expression of HSP70 and HSP27 (47, 48). RGRN-305 treatment was associated with an increased expression of *HSPA1A* (HSP70), indicating a heat shock response. However, the clinical significance of a heat shock response in inflammatory skin diseases is still not fully understood (31). HSP70 has been reported to elicit immunosuppressive effects in an animal model of atopic dermatitis, whereas HSP70 has displayed both anti- and pro-inflammatory effects in other models of inflammatory skin diseases (49–52). Further research is needed to determine the role of a heat shock response in atopic dermatitis.

Limitations of the study include the inherent nature of basic research and experimental models that may encounter challenges when translating the findings into clinical practice. Nonetheless, the accumulating evidence of HSP90 inhibition exerting broad anti-inflammatory effects together with the findings from this study increases the likelihood of clinical success in treating AD. Another limitation is that the effects of RGRN-305 in specific cell types beyond keratinocytes have not been evaluated. However, the mouse experiments capture the complex interplay and contribution of various cell types in the skin. Lastly, the permeability of mice skin varies to human skin, and therefore further studies should evaluate optimal drug formulations enhancing the permeation and efficacy of topical RGRN-305.

In conclusion, HSP90 inhibition by RGRN-305 potently suppressed inflammation using *in vitro* and *in vivo* experimental models mimicking AD, providing evidence that HSP90 inhibition may be a novel mechanism of action in treating AD.

## Data availability statement

The datasets presented in this study can be found in online repositories. The names of the repository/repositories and accession number(s) can be found below: https://www.ncbi.nlm.nih.gov/geo/query/acc.cgi?&acc=GSE246569.

## Ethics statement

The studies involving humans were approved by Central Jutland Regional Committee on Health Research Ethics (M-20110027). The studies were conducted in accordance with the local legislation and institutional requirements. The participants provided their written informed consent to participate in this study. The animal study was approved by The Danish Animal Experiments Inspectorate (2022-15-0201-01267). The study was conducted in accordance with the local legislation and institutional requirements.

## Author contributions

HB: Conceptualization, Formal analysis, Funding acquisition, Investigation, Methodology, Visualization, Writing – original draft, Writing – review & editing. AB: Methodology, Supervision, Writing – review & editing. GG: Conceptualization, Funding acquisition, Writing – review & editing. LI: Conceptualization, Funding acquisition, Methodology, Supervision, Writing – review & editing. CJ: Conceptualization, Formal analysis, Funding acquisition, Investigation, Methodology, Supervision, Writing – review & editing.

## Supplementary material

Supplementary Appendix S1 Study report from Comparative Biosciences, Inc.

Supplementary Figure 1 Cytotoxicity of RGRN-305 in stimulated primary human keratinocytes.

Supplementary Figure 2 Relative body weights throughout the experiments.

Supplementary Figure 3 Ear thickness measures from experiments conducted at Comparative Biosciences, Inc.

Supplementary Figure 4 Gene expression of *HSPA1A/Hspa1a* and *HSPB1/Hspb1*.

Supplementary Table 1 Lists of all genes derived from the differential expression analyses.
