# Supplementary material for: Heat shock protein 90 inhibition attenuates inflammation in models of atopic dermatitis: a novel mechanism of action
Source: Front Immunol. 2024 Jan 11;14:1289788. doi: 10.3389/fimmu.2023.1289788 (PMC10808526; doi:10.3389/fimmu.2023.1289788)
Supplement: Supplementary Appendix S1 — Study report from Comparative Biosciences, Inc. [file DataSheet_1.docx]

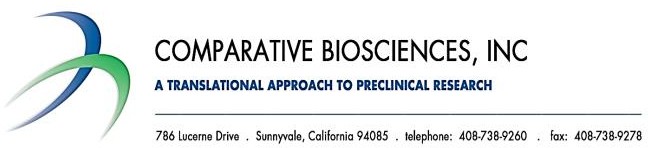


# STUDY REPORT

**Model Development and Efficacy Study of an Orally Administered HSP90 Inhibitor (RGRN-305) in Atopic Dermatitis Induced by MC903 in BALB/c Mice**

# Study Number: CB21-5006-M-EF IACUC Proposal Number: CB21-5006-M-EF

## Testing Facility:

**Comparative Biosciences, Inc.**

## 786 Lucerne Drive

**Sunnyvale, CA 94085**

## Sponsor:

**Regranion, LLC 194 Ionsborough Street**

## Mount Pleasant, SC 29464

**Sponsor's Test Article:**

**RGRN-305**


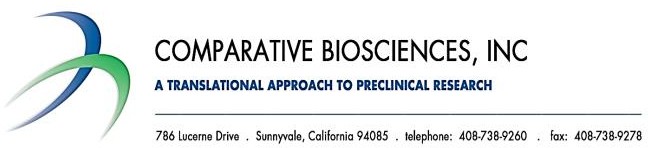


# KEY PERSONNEL

**Model Development and Efficacy Study of an Orally Administered HSP90 Inhibitor (RGRN-305) in Atopic Dermatitis Induced by MC903 in BALB/c Mice**

# Study Number: CB21-5006-M-EF IACUC Proposal Number: CB21-5006-M-EF

### Key Study Personnel:

Study Director: T. Kristian von Almen, PhD

Lead Biologist: Ethan Haslett

Attending Veterinarian: Miranda Abrahams, DVM Histopathology: Carol Meschter, DVM, PhD, DACVP Histology Manager: Fariba Chalajour, PhD

Testing Laboratory: Comparative Biosciences, Inc.

786 Lucerne Drive

Sunnyvale, CA 94085

Histopathology: Carol Meschter, DVM, PhD, DACVP

Comparative Biosciences, Inc.

Test Sites: Additional Tissue Analysis

Icahn School of Medicine at Mount Sinai Icahn Medical Institute #13-76

1425 Madison Ave New York, NY 10029 ATTN: Yeriel Estrada

Sponsor Representative: Gautam Ghatnekar, PhD

Chief Executive Officer Phone: 843-860-8785

Email: [ghatnekar@regranion.com](mailto:ghatnekar@regranion.com)

### Key Study Dates:

Study Initiation: 30 July 2021

Study Completion: Date of final report signature


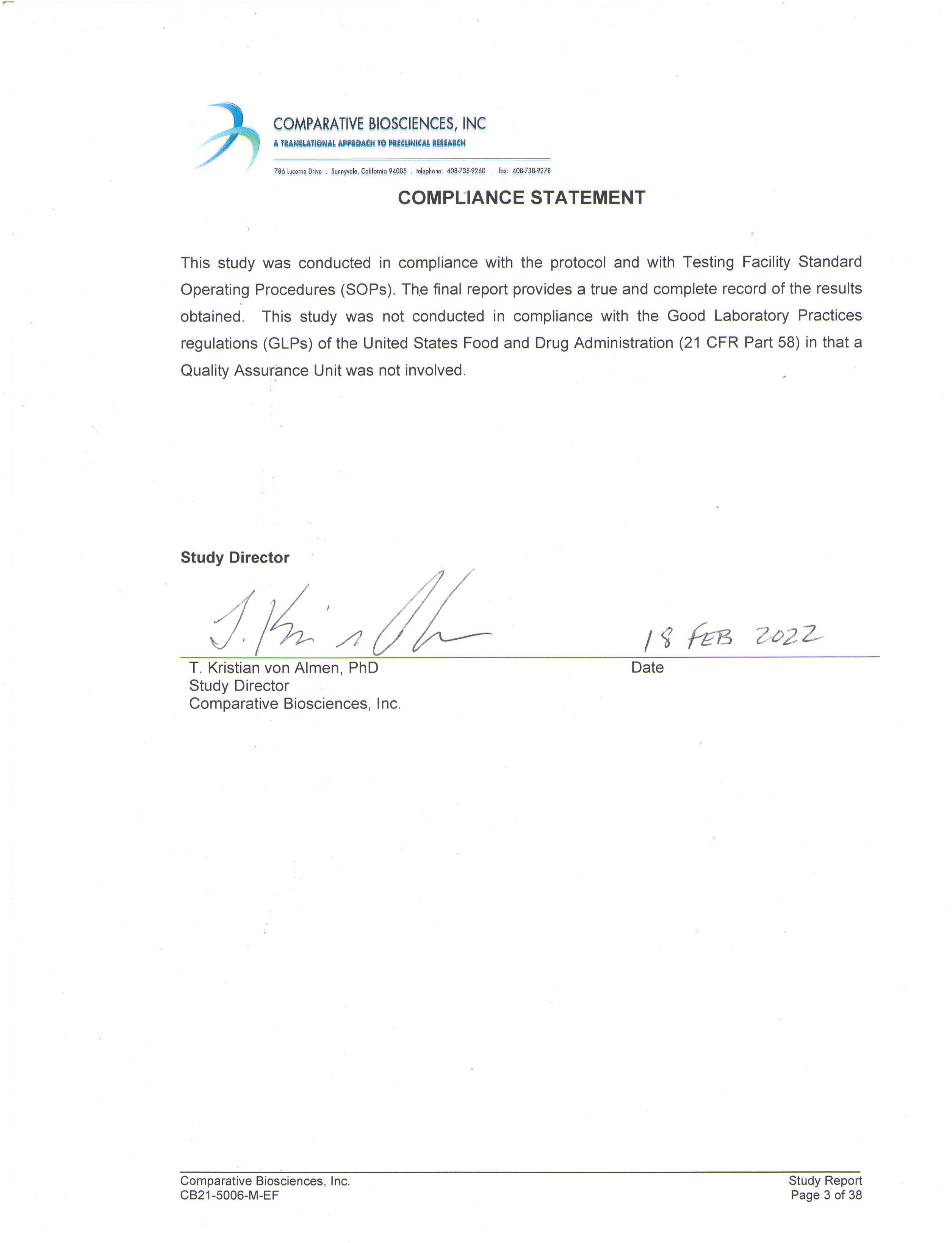


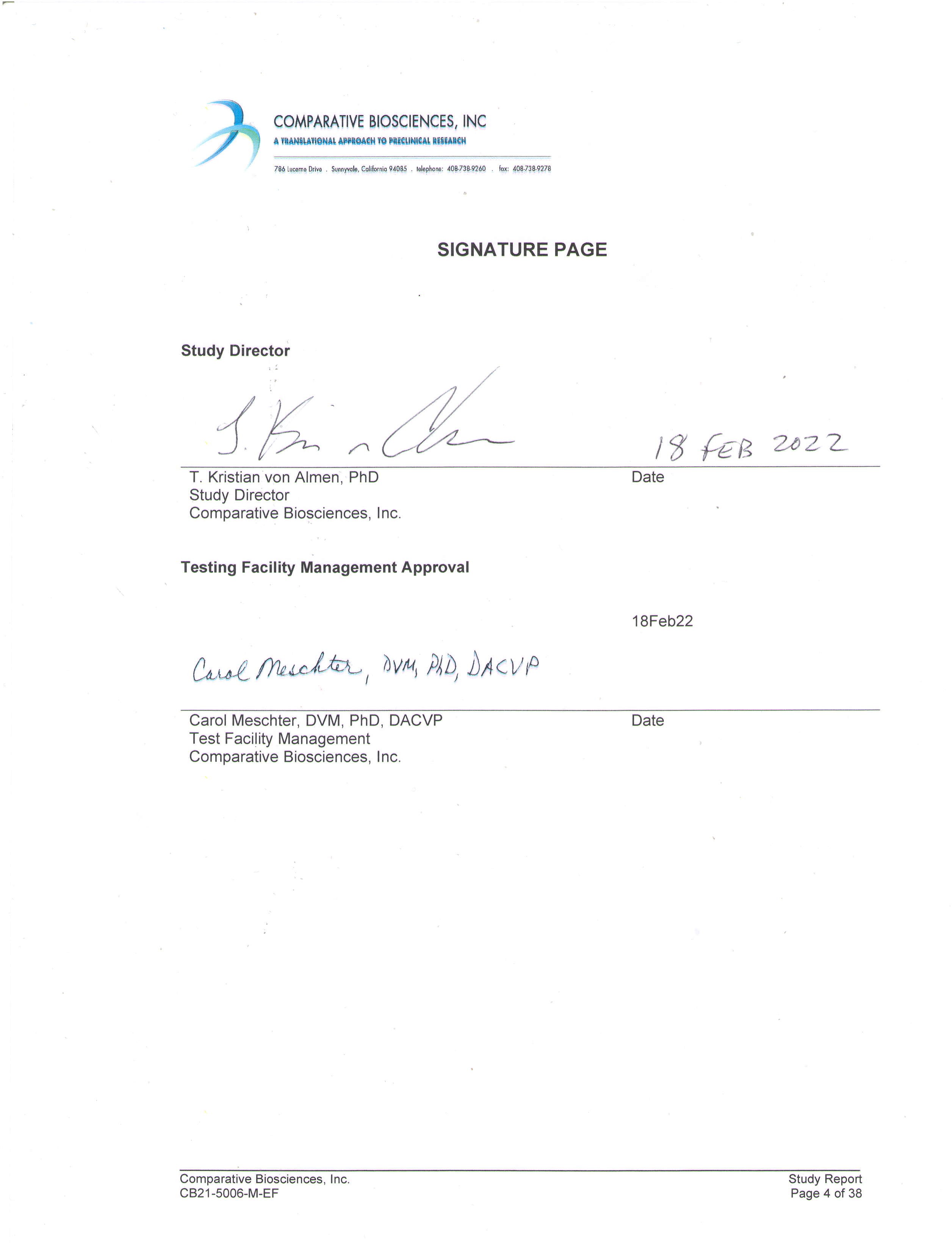


**TABLE OF CONTENTS**

(This report consists of 38 pages plus 3 appendices, which are numbered separately.)

[KEY PERSONNEL 2](#_bookmark0)

[COMPLIANCE STATEMENT 3](#_bookmark1)

[SIGNATURE PAGE 4](#_bookmark2)

[TABLE OF CONTENTS 5](#_bookmark3)

[SUMMARY 7](#_bookmark4)

1. [INTRODUCTION 8](#_bookmark5)
2. [STUDY DESIGN 8](#_bookmark6)

[Table 1. Summary of Pilot Study Design 9](#_bookmark7)

[Table 2. Summary of Efficacy Study Design 9](#_bookmark8)

1. [MATERIALS AND METHODS 9](#_bookmark9)
   1. [Test and Control Articles 9](#_bookmark10)
   2. [Dose Preparation 10](#_bookmark11)
   3. [Stability Under the Conditions of Administration 10](#_bookmark12)
   4. [Test System 11](#_bookmark13)
   5. [Atopic Dermatitis Induction 13](#_bookmark14)
   6. [Dose Administration 13](#_bookmark15)
   7. [Referrence1 13](#_bookmark16)
2. [MEASURES AND ASSESSMENTS 13](#_bookmark18)
   1. [Clinical Observations 13](#_bookmark19)
   2. [Body Weights 14](#_bookmark20)
   3. [Food Consumption 14](#_bookmark21)
   4. [Water Consumption 14](#_bookmark22)
   5. [Draize Scoring 14](#_bookmark23)
   6. [Ear Thickness Measures 14](#_bookmark24)
   7. [Ear Photography 14](#_bookmark25)
   8. [Euthanasia and Tissue Collection 14](#_bookmark26)
   9. [Histopathology 14](#_bookmark27)
   10. [Statistical Analysis 15](#_bookmark28)
   11. [Archives 15](#_bookmark29)
3. [RESULTS 15](#_bookmark30)
   1. [Model Induction 15](#_bookmark31)
   2. [Acclimation 15](#_bookmark32)
   3. [Mortality 15](#_bookmark33)
4. [RESULTS (Pilot Study) 15](#_bookmark34)
   1. [Clinical Observations 15](#_bookmark35)
   2. [Body Weights 16](#_bookmark36)
   3. [Draize Scores 16](#_bookmark37)
   4. [Ear Thickness Scores 16](#_bookmark38)
   5. [Euthanasia 16](#_bookmark39)
   6. [Histopathology 16](#_bookmark40)
   7. [Summary and Conclusions 16](#_bookmark41)
5. [RESULTS (Efficacy Study) 17](#_bookmark42)
   1. [Clinical Observations 17](#_bookmark43)
   2. [Body Weights 17](#_bookmark44)
   3. [Draize Scores 17](#_bookmark45)
   4. [Ear Thickness Scores 17](#_bookmark46)
   5. [Ear Photographs 18](#_bookmark47)
   6. [Necropsy 18](#_bookmark48)
   7. [Histopathology 18](#_bookmark49)
6. [DISCUSSION AND CONCLUSIONS 18](#_bookmark50)
7. [DATA TABLES and FIGURES 20](#_bookmark51)

[PILOT STUDY DATA 21](#_bookmark52)

[Table 3. Clinical Observations (Pilot Study) 22](#_bookmark53)

[Table 4. Absolute and Relative Body Weights (Pilot Study) 22](#_bookmark54)

[Table 5. DRAIZE Scores (Pilot Study) 23](#_bookmark55)

[Table 6. Ear Thickness Measures (Pilot Study) 24](#_bookmark56)

[Table 7. Histology Scores (Pilot Study) 25](#_bookmark57)

[EFFICACY STUDY DATA 26](#_bookmark58)

[Table 8. Clinical Observations (Efficacy Study) 27](#_bookmark59)

[Table 9. Absolute and Relative Body Weights (Efficacy Study) 28](#_bookmark60)

[Table 10. Draize Scores (Efficacy Study) 29](#_bookmark61)

[Table 11. Ear Thickness Measures (Efficacy Study) 32](#_bookmark62)

[Figure 1. Representative Ear Photographs, Day 0 34](#_bookmark63)

[Figure 2. Representative Ear Photographs, Day 6 35](#_bookmark65)

[Figure 3. Representative Ear Photographs, Day 9 36](#_bookmark66)

[Figure 4. Representative Ear Photographs, Day 9 37](#_bookmark67)

[Table 12. Histology Scores (Efficacy Study) 38](#_bookmark68)

[APPENDIX A: Protocol and Amendment A1](#_bookmark69)

[APPENDIX B: Certificates of Analysis and MSDS B1](#_bookmark100)

[END of REPORT 63](#_bookmark101)

## SUMMARY

The objective of this study was to provide data on the efficacy of an orally administered HSP90 inhibitor (RGRN-305) in atopic dermatitis induced by MC903 in mice. Initially, a pilot study was conducted to assure MC903 would induce atopic dermatitis and that treatment with a positive control (dexamethasone) would ameliorate the dermatitis. Atopic dermatitis was successfully induced in animals with topical administration of MC903[1](#_bookmark16) applied to both shaved ears from Day 0- 4 and Day 7-8. Group 1 received no treatment; Group 2 was treated orally or subcutaneously with dexamethasone. Clinical observations, body weight, Draize scores (erythema, edema) and ear thickness measures were taken during in-life, beginning Day 0 (prior to induction). Ears were collected and evaluated histopathologically post treatment. Results from the pilot study indicated successful induction of atopic dermatitis with MC903. Untreated animals exhibited both clinical and histological signs of atopic dermatitis, specifically increased ear thickness, erythema, edema and histologic findings. Animals treated with dexamethasone lost weight and exhibited less severe clinical and histologic findings compared to untreated controls. Animals treated subcutaneously with dexamethasone had less severe histological findings than orally treated animals. As such, subcutaneous dexamethasone was used as the Positive Control in the efficacy study.

Efficacy study consisted of the following. Group 1 were naïve, untreated controls. Atopic dermatitis was induced in Groups 2-7 with topical administration of MC903[1](#_bookmark16) (per above). Mice in Groups 2-7 were administered no treatment, oral Vehicle, oral Test Article (RGRN-305 at low, medium or high dose), or subcutaneous dexamethasone prior to induction of skin inflammation, then daily thereafter through in-life. Materials and methods were the same as the pilot study (above) with exception that terminal blood was collected (cytokine analysis) at termination in the efficacy study. Efficacy study results indicated that atopic dermatitis was successfully induced with MC903. RGRN-305 was well tolerated clinically and dexamethasone treated animals lost weight as expected. Induced, untreated, Vehicle and low dose RGRN-305 treated animals exhibited severe clinical and histological signs of atopic dermatitis, specifically increased ear thickness, erythema, edema and histologic findings. Animals treated with mid and high dose RGRN-305 exhibited moderate, less severe clinical and histologic findings compared to untreated and Vehicle controls. Animals treated with subcutaneous dexamethasone had minimal clinical and histological findings compared to untreated and Vehicle controls. As such, mid and high dose RGRN-305 effectively ameliorated clinical and histologic signs of atopic dermatitis but were markedly less effective than dexamethasone.

## INTRODUCTION

The objective of this study was to provide data on the efficacy of an orally administered HSP90 inhibitor (RGRN-305) in atopic dermatitis (AD) induced by MC903 in mice. Initially, a pilot study was conducted which demonstrated that MC903 induced atopic dermatitis and that treatment with Positive Control (dexamethasone) ameliorated the dermatitis.

## STUDY DESIGN

The study protocol and one amendment are included [Appendix A**.**](#_bookmark69) The study design is outlined in [Table 1](#_bookmark7) (Pilot Model Development Study) and [Table 2](#_bookmark8) (Efficacy Study), below. Pilot study consisted of two groups of 4 animals each (2 male, 2 female). AD was induced bilaterally in shaved ears of all mice ([Table 1](#_bookmark7)) with topical application of MC903[1](#_bookmark16) applied to both shaved ears from Day 0-4 and Day 7-8. Group 1 received no treatment; Group 2 was treated orally or subcutaneously (SC) with dexamethasone prior to induction of skin inflammation, then daily thereafter through in-life. Daily clinical observations, body weights, Draize scores, ear thickness measures and photos were recorded. Ears were collected for histopathologic evaluation at termination.

Efficacy study ([Table 2](#_bookmark8)) consisted of the following. Group 1 were naïve, untreated controls. AD was induced in Groups 2-7 with topical administration of MC903[1](#_bookmark16) (per above). Mice in Groups 2-7 were administered no treatment, oral Vehicle, oral RGRN-305 (low, medium or high dose), or SC dexamethasone prior to induction, then daily thereafter through in-life. Clinical observations, body weights, Draize scores and ear thickness were measured and photos were taken. Terminal blood and ears were collected at end life. Ears (punch biopsies) were standardly processed for histopathology. Slides were read by a board-certified veterinary pathologist. Additional ear samples (formalin fixed, snap frozen and stored in RNAlater) were collected and shipped to the Icahn School of Medicine at Mount Sinai (New York, NY) for analysis and reporting. Carcasses were disposed of with no further analysis.

### Table 1. Summary of Pilot Study Design

| **Group** | **Animal Numbers** | **Induction1 (topical)** | **Treatment2** | **In-Life Duration** | **Tests or Assays** |
| --- | --- | --- | --- | --- | --- |
| **1** | **101-102M** | MC903  25 µl per ear (12.5 µl dorsal,  12.5 µl ventral) (1.125nmol per ear)  Applied to both shaved ears (Day 0 – Day 4;  Day 7-8) | NA | Acclimation  +  9 Days | - Daily clinical observations - Weekly body weights - Daily Draize scoring^3^ - Ear thickness3, with photos |
|  | **151-152F** |  |  |  |  |
| **2** | **203-204M** |  | Positive Control (Dexamethasone) Oral or SC (5mg/kg) |  |  |
|  | **253-254F** |  |  |  |  |

M=male, F=female, ^1^ Administered 1 hour after Test/Control Article, ^2^ Daily administration Day 0-Day 8, SC=subcutaneous,

^3^ Measurements prior to Test Article administration-weekdays only

### Table 2. Summary of Efficacy Study Design

| **Group** | **Animal Numbers** | **Induction1 (topical)** | **Treatment2** | **Dose (mg/kg)** | **In-Life Duration** | **Tests or Assays** |
| --- | --- | --- | --- | --- | --- | --- |
| **1** | **101M**  **151F** | N/A | Naïve, untreated | N/A | Acclimation  +  9 Days | - Daily clinical observations - Weekly body weights - Daily Draize scoring, ear thickness^3^, with photos (weekdays only) - Necropsy, collect ears - Histopathology of ears - Remaining ear tissue4:   1) fixed in formalin   1. snap frozen 2. stored in RNAlater |
| **2** | **201-202M**  **251-252F** | MC903  Topical Administration  25 µl per ear (12.5 µl dorsal,  12.5 µl ventral) (1.125nmol per ear)  Applied to both shaved ears Day 0 – Day 4;  Day 7-8 | Model Control  (No treatment) | N/A |  |  |
| **3** | **301-302M**  **351-352F** |  | Vehicle  oral | N/A |  |  |
| **4** | **401-403M**  **451-453F** |  | RGRN-305  oral | 20 |  |  |
| **5** | **501-503M**  **551-553F** |  | RGRN-305  oral | 50 |  |  |
| **6** | **601-603M**  **651-653F** |  | RGRN-305  oral | 100 |  |  |
| **7** | **701-703M**  **751-753F** |  | Positive Control (Dexamethasone) SC  (10mg/kg) | 5 |  |  |

M=male, F=female, ^1^ Administered 1 hour after Test/Control Article, ^2^ Daily administration Day 0-Day 8, SC=subcutaneous,

^3^ Measurements prior to Test Article administration, ^4^Sent to Sponsor’s lab for analysis and reporting

## MATERIALS AND METHODS

### Test and Control Articles

- - 1. **Test Article**

Test Article, RGRN-305 (an HSP90 Inhibitor), was provided by Sponsor as a lyophilized powder. It was received in good condition on 18 August 2021. Certificate of Analysis and MSDS are attached in [Appendix B.](#_bookmark100) Test Article was stored at 4ºC.

### Vehicle Article

Vehicle Article was 5% Kleptose solution (pH 4.5). The Testing Facility obtained the Vehicle Article from a commercial source and provide an amount sufficient for use in the study. Purity and identity of the Vehicle Article was accepted based on the product labeling. Vehicle solution was stored at 4ºC.

### Positive Control Article

Positive Control Article was Dexamethasone. The Testing Facility obtained the Positive Control Article from a commercial source and provide an amount sufficient for use in the study. Purity and identity of the Positive Control Article was accepted based on the product labeling. Positive Control Article was stored at room temperature.

### Dose Preparation

Test Article and Vehicle were prepared as follows.

Test Article: RGRN-305

Test Article solution was prepared in batches of 15 mL, stored at 4°C and used within one week.

- 120 mg of RGRN-305 was mixed with 11 mL of vehicle solution in a glass vial
- The solution was mixed by vortexing for 1 minute
- The pH was adjusted to 4.5 with 0.1N HCl (approximately 500 uL), the solution was vortexed regularly during pH adjustment to solubilize particles remaining on the vial wall
- The solution was vortexed for 10 min, this was repeated as needed if particles remained visible
- The solution was completed with vehicle solution up to 15 mL
- The solution was filtered on 0.2 or 0.22 um GHP or PSE filters and stored in a sterile vial

Vehicle: 5% Kleptose solution (pH 4.5)

Vehicle solution was prepared in batches of 30 mL, stored at 4°C and used within one week.

- 1.5 g of Kleptose (HPB Oral grade) was dissolved in 20 mL deionized water in a glass vial
- The solution was mixed by vortexing for 1 minute
- The pH was adjusted to 4.5 with 0.1N HCl
- The solution was completed with deionized water up to 30 mL
- The solution was filtered on 0.2 or 0.22 um GHP or PSE filters and stored in a sterile vial

Oral Vehicle and Test Articles were administered daily, starting on Day 0 one hour before the induction administration. Group 1 received Vehicle and Groups 2-4 – RGRN-305 at low, medium and high dose, respectively.

### Stability Under the Conditions of Administration

On the day of dosing, Test Article, RGRN-305, was diluted in Vehicle to permit dosing at low (20 mg/kg), medium (50 mg/kg) and high dose (100 mg/kg). Dose volumes were

calculated based on body weights. Animals were dosed orally via gavage. Dosing solutions were considered stable under the conditions of the study.

### Test System

Total 42 mice (*Mus musculus*; 21 males, 21 females) on study plus 2 male and 2 female spares (Model development = 4M, 4F; Main Study = 17M, 17F) were obtained from Charles River Laboratory (5 November 2021).

### Institutional Animal Care and Use Committee Approval

This study was conducted in accordance with a research proposal approved by the Institutional Animal Care and Use Committee of Comparative Biosciences, Inc.

### Receipt, Quarantine and Acclimation

The animals were received in good condition on 5 November 2021 and acclimated for 3 days. During the acclimation period, the mice were observed at least once daily for clinical signs of abnormality. No unusual signs were observed. On 8 November 2021, all of the animals appeared clinically normal and were released by Testing Facility veterinarian for use in the study.

### Identification

The mice were arbitrarily assigned sequential temporary animal identification numbers upon receipt at the Testing Facility. During the acclimation period and until assignment to study groups, the animals were identified by study number and temporary identification numbers displayed on each cage card. On 8 November 2021, the mice were weighed and randomly assigned to study groups. Upon assignment to a study group, each animal was ear-marked with a unique permanent identification number using indelible ink. A cage card displaying the animals’ permanent identification number and study number was displayed throughout the in-life period. The permanent animal identification numbers and their groups are shown in the Summary of Study Design Table above.

### Dosing Cohorts

Animals were dosed as a single cohort. The dose and route of administration were selected by the Sponsor based on the anticipated clinical use.

### Temperature and relative humidity

Controls were set to maintain the temperature and relative humidity of the animal rooms at 68-79ºF (20-26ºC) and 30-70%, respectively. These environmental parameters were monitored and recorded daily. During the treatment phase of the study, there were minor excursions from the specified temperature and humidity range. These minor temperature and humidity excursions were not considered to have affected the study results.

### Light cycle

Twelve hours of light and twelve hours of dark were provided in the animal rooms. A fluorescent light source was used, with lights turned on at approximately 0700 hours and turned off at approximately 1900 hours each day.

### Feed

LabDiet® 5001 Rodent Diet (Purina Mills, Inc., St. Louis, MO) or other approved diet were provided ad libitum throughout the acclimation and in-life phases. Lot number(s) and Certificate(s) of Analysis (as applicable) were maintained by the Testing Facility. There are no known contaminants that are reasonably expected to be present in the diet that are known to be capable of interfering with the purpose or conduct of the study.

### Water

Fresh water from Sunnyvale Municipal Water Supply was provided *ad libitum* to the animals via a rack watering system. The water supply is periodically monitored for chlorine content and bacterial contamination. Results of these analyses are maintained on file at the testing facility. There are no known contaminants that are reasonably expected to be present in the water that are known to be capable of interfering with the purpose or conduct of the study.

### Husbandry

Animals were group-housed in stainless steel cages. General procedures for animal housing and husbandry were conducted according to testing facility SOPs and met all regulations concerning use of animals in research, including the U.S. Department of Agriculture regulations (9 CFR Chapter 1) implementing the Animal Welfare Act (7 USC 2131 *et seq*.) and the recommendations of the National

Academy Press, 2011).

### Sanitation

All animal enclosures and equipment were cleaned and sanitized according to Testing Facility SOP.

### Final Selection and Randomization of Animals

No earlier than one (1) day prior to dosing, all animals were examined for general health by a qualified veterinarian. Animals not excluded for health reasons were weighed and selected for the study based on normal clinical presentation and moderate body weight. Animals were randomly assigned study groups. Details of the allocation procedure were filed with the raw data.

### Atopic Dermatitis Induction

Atopic Dermatitis (AD) was induced bilaterally in shaved ears of all mice ([Table 1,](#_bookmark7) [ref 1](#_bookmark17)) with topical application of MC903 (1.125nmol per ear or 25 µl total: 12.5 µl on dorsal,

12.5 µl ventral side of each ear) on Day 0-4 and 7-8. MC903 was administered 1-hour after Test or Control Article treatment.

### Dose Administration

On the day of dosing, Test Article, RGRN-305 8 mg/mL, was diluted in Vehicle to permit dosing at low (20 mg/kg), medium (50 mg/kg) and high dose (100 mg/kg). Dose volumes were calculated based on body weights. Animals were dosed orally via gavage.

### Referrence^1^

Moosbrugger-Martinz V, Schmuth M, Dubrac S. A Mouse Model for Atopic Dermatitis Using Topical Application of Vitamin D3 or of Its Analog MC903. Methods Mol Biol.

2017;1559:91-106. DOI: 10.1007/978-1-4939-6786-5_8. PMID: 28063040.

## MEASURES AND ASSESSMENTS

### Clinical Observations

Clinical observations, including overt signs of non-normal response were recorded once daily in all animals from Day 0 and throughout the study duration. All signs of clinical abnormality were recorded.

### Body Weights

The animals were weighed prior to Day 0, then weekly during study conduct.

### Food Consumption

Food consumption was not measured.

### Water Consumption

Water consumption was not measured.

### Draize Scoring

Bilateral DRAIZE scores were recorded daily (weekdays only), prior to Test Article administration.

### Ear Thickness Measures

Bilateral ear thickness (mm) was measured daily (weekdays), prior to Test Article administration.

### Ear Photography

Bilateral ear photographs were taken daily (weekdays), prior to Test Article administration.

### Euthanasia and Tissue Collection

The animals were euthanized on Day 9. Ears were collected and processed as follows. Punch biopsies (3 mm, taken from the center of the injection area) were standardly processed for histology evaluation at the Testing Facility. Remaining ear tissue from efficacy study animals was divided into three parts and processed for additional analysis. A section of each ear tissue was formalin fixed, another section snap frozen and another section was stored in RNAlater. All samples were shipped to Icahn School of Medicine, Mount Sinai (New York, NY) for analysis. Carcasses were disposed of with no further analysis.

### Histopathology

Bilateral ear tissue (punch biopsies) was examined histopathologically. Tissues were dehydrated, embedded in paraffin, sectioned at 3-5 µm, and stained with hematoxylin and eosin. Slides were evaluated via light microscopy by a board-certified veterinary pathologist including evaluation of the epidermis, subdermis and presence of inflammation. Changes were scored using the industry standard 5-point scoring system (Mann, et all, 2012).

### Statistical Analysis

Means and standard deviations were calculated using Microsoft Excel®. Results are detailed in tables and graphically in the report. Due to the small group size, statistical analysis was not performed data.

### Archives

Original raw data, or exact copies, will be stored under the control of the Testing Facility. The final report will be delivered to the Sponsor and a copy will be retained at the Testing Facility. All specimens will be returned to the Sponsor or to a facility to be designated by the Sponsor for storage.

## RESULTS

Pilot study data is presented in [Tables 3-7,](#_bookmark53) efficacy study data is located in [Tables 8-12.](#_bookmark59)

### Model Induction

MC903 successfully induced AD in both the pilot and efficacy study animal ears. Increases in erythema (redness), edema (swelling) and ear thickness measures were evident beginning on Day 3, which became more severe over time, especially in untreated animals. In addition, significant histologic findings were evident in induced, untreated or Vehicle treated ears in comparison to induced, dexamethasone treated ears. As such, dexamethasone successfully ameliorated clinical and histological signs of AD in this study.

### Acclimation

Animals in the pilot and efficacy study were acclimated for a minimum of three (3) days after receipt at Comparative Biosciences, Inc. Animals were observed daily during the acclimation period for clinical signs of abnormality. No unusual clinical signs were observed during the acclimation period in pilot or efficacy study animals.

### Mortality

There was no mortality in pilot or efficacy study.

## RESULTS (Pilot Study)

### Clinical Observations

Clinical observations are summarized in [Table 3.](#_bookmark53) A couple animals in each group were observed to be hunched or to have rough coat. This finding occurred in untreated and treated (dexamethasone) animals and is likely due to handling or induction procedure.

### Body Weights

Individual and group mean absolute and relative (% of Day 0) body weights are presented in [Tables 4.](#_bookmark54) Animals is both groups lost about 10% (Day 7) and 20% (Necropsy) of baseline weight. As expected, dexamethasone treated animals lost slightly more weight than untreated animals.

### Draize Scores

Individual and group mean Draize scoring of ears is detailed in [Table 5.](#_bookmark55) Erythema and edema were evident in ears of treated and untreated animals starting on Day 3. Findings were less severe in dexamethasone treated animals compared to untreated animals that evidenced increased signs of AD over time. There was no appreciable difference in findings between oral and SC dexamethasone treated animals.

### Ear Thickness Scores

Individual and group mean absolute and relative (% of Day 0) ear thickness measures are presented in [Table 6.](#_bookmark56) Ear thickness (mm) measures began increasing on Day 4 in treated and untreated animals. Measures were lower in dexamethasone treated animals compared to control animal values which increased significantly over time. There was no difference in oral and SC dexamethasone treated animals.

### Euthanasia

The animals were euthanized on Day 9 per CBI SOP. Ears were collected; punch biopsies (3 mm, taken from the center of the treatment area) were taken and standardly processed for histology evaluation at Testing Facility.

### Histopathology

Histopathological evaluation of ear biopsies is in [Table 7.](#_bookmark57) There was severe acute suppurative inflammation with edema, hyperemia and marked hyperplasia of the epithelium with multifocal ulceration and loss of the epithelium in Vehicle and orally treated (dexamethasone) animals. These findings were less severe (moderate) in subcutaneously treated (dexamethasone) animals.

### Summary and Conclusions – Pilot Study

AD was successfully induced with MC903 in the pilot, model development study. More severe clinical (erythema, edema, ear thickness) and histological (inflammation, edema, hyperemia, hyperplasia, ulceration) findings were observed in induced, untreated animals compared to those treated with dexamethasone. Animals treated SC with dexamethasone

had less severe findings than untreated or orally treated dexamethasone treated animals. As such, efficacy study used SC dexamethasone as the Positive Control treatment.

## RESULTS (Efficacy Study)

### Clinical Observations

Clinical observations are summarized in [Table 8.](#_bookmark59) There were no Test Article related clinical observations recorded. Incidental findings included bite marks (likely cage fighting) and rough coat in a couple animals.

### Body Weights

Individual and group mean absolute and relative (% of Day 0) body weights are presented in [Table 9.](#_bookmark60) Uninduced animals gained weight while induced, untreated animals lost about 6% of baseline weight during in-life. There was no appreciable Test Article effect on body weight as Vehicle and Test Article treated animals lost from 12% (Vehicle) to 8-12% (Test Article treated) of baseline body weight during study. Findings indicate that induction accounted for slight weight loss during study. As expected, dexamethasone treated animals lost substantial weight (15% at Day 7 and 22% at Day 9) during study.

### Draize Scores

Individual and group mean Draize scoring of ears is detailed in [Table 10.](#_bookmark61) No findings were recorded in uninduced, untreated animals (Group 1). Increased erythema and edema were evident in induced, untreated and Vehicle treated groups (Group 1 and 2). These observations were slightly less severe in low dose animals (Group 4) and findings were substantially less in mid and high dose animals (Group 5, Group 6) indicating that Test Article at these doses minimized clinically observed erythema and edema. As expected, dexamethasone treated animals displayed virtually no signs of erythema or edema during in-life.

### Ear Thickness Scores

Individual and group mean absolute and relative (% of Day 0) ear thickness measures (mm) are detailed in [Table 11.](#_bookmark62) Values in control, uninduced animals (Group 1) remained minimal during in-life. Thickness measures in induced, untreated (Group 2), Vehicle (Group 3) and low dose treated (Group 4) animals significantly increased (~2x) over time compared to control animals. Ear thickness was slightly improved in mid (Group 5) and high (Group 6) dose animals. As expected, dexamethasone treated animals evidenced only a slight increase in thickness and values were similar to control animals.

### Ear Photographs

Representative photographs of ears are displayed in [Figure 1 – 4.](#_bookmark63)

### Necropsy

The animals were euthanized on Day 9 per CBI SOP. Ears were successfully collected; punch biopsies (3 mm, taken from the center of the treatment area) were taken and standardly processed for histology evaluation. Remaining ear tissue from efficacy study animals was divided into three parts and processed for additional analysis. A section of each ear tissue was formalin fixed, another section snap frozen and another section was stored in RNAlater. All samples were shipped to Icahn School of Medicine, Mount Sinai (New York, NY) for analysis. Carcasses were fixed in 10% neutral buffered formalin for subsequent analysis by Additional Work Order.

### Histopathology

Histopathological evaluation of ear biopsies is in [Table 12.](#_bookmark64) There were no findings in control, uninduced animals. There was severe acute suppurative inflammation with edema, hyperemia and marked hyperplasia of the epithelium with multifocal ulceration and loss of the epithelium in induced, untreated (Group 2) Vehicle (Group 3) and low dose treated (Group 4) animals. These findings were less severe (moderate) in mid (Group 5) and high dose (Group 6) treated animals. Findings were mild in dexamethasone treated animals, as expected.

## DISCUSSION AND CONCLUSIONS

The objective of this study was to provide data on the efficacy of an orally administered HSP90 inhibitor (RGRN-305) in AD induced by MC903 in mice. Initially, a pilot study was conducted which demonstrated successful induction of AD which was ameliorated with positive control (dexamethasone). Untreated animals exhibited both clinical and histological signs of atopic dermatitis, specifically increased ear thickness, erythema, edema and histologic findings. Animals treated with dexamethasone lost weight and exhibited less severe clinical and histologic findings compared to untreated controls. Animals treated subcutaneously with dexamethasone had less severe histological findings than orally treated animals. As such, subcutaneous dexamethasone was used as the Positive Control in the efficacy study.

Efficacy study consisted of the following. Group 1 were naïve, untreated controls. Atopic dermatitis was induced in Groups 2-7 with topical administration of MC903[1.](#_bookmark16) Mice in Groups 2-7 were administered no treatment, oral Vehicle, oral RGRN-305 (low, medium or high dose),

or subcutaneous dexamethasone prior to induction, then daily thereafter. Clinical observations, body weight and ear assessments were recorded during in-life. Ears and terminal blood were collected at termination. Efficacy study results indicated that atopic dermatitis was successfully induced with MC903. RGRN-305 was well tolerated clinically and dexamethasone treated animals lost weight as expected. Induced, untreated, Vehicle and low dose RGRN-305 treated animals exhibited severe clinical and histological signs of atopic dermatitis, specifically increased ear thickness, erythema, edema and histologic findings. Animals treated with mid and high dose RGRN-305 exhibited moderate, less severe clinical and histologic findings compared to untreated and Vehicle controls. Animals treated with subcutaneous dexamethasone had minimal clinical and histological findings compared to untreated and Vehicle controls. As such, mid and high dose RGRN-305 ameliorated clinical and histologic signs of atopic dermatitis but were markedly less effective than dexamethasone.

1. **DATA TABLES and FIGURES**

# PILOT STUDY DATA

### Table 3. Clinical Observations (Pilot Study)

| **Group** | **Animal Number** | **Clinical Observation (days observed)** |
| --- | --- | --- |
| **1** | **101** | NSO (Day 0 - 6), Slight redness of ears (Day 7 - 10), Slight redness of ears, white spot on back of left ear, TS (Day 11) |
|  | **102** | NSO (Day 0 - 6), Slight redness of ears (Day 7 - 10), Redness of ears, TS (Day 11) |
|  | **151** | NSO (Day 0 - 6), Slight redness of ears, scabbing on back of right ear (Day 7), Swelling and redness of ears, scabbing on back of right ear (Day 8 - 9), Hunched, swelling and redness of ears, scabbing on back of right ear (Day 10), Hunched with  rough coat, severe swelling and redness of ears, TS (Day 11) |
|  | **152** | NSO (Day 0 - 6), Slight redness of ears (Day 7 - 9), Swelling and redness of ears (Day 10), Hunched, swelling and redness of ears, TS (Day 11) |
| **2** | **201** | NSO (Day 0 -7), Slight redness of ears (Day 8 - 10), Slight redness and swelling of ears, TS (Day 11) |
|  | **202** | NSO (Day 0 -2), Redness around edge of right ear (Day 3), Redness on both ears  (Day 4 - 6), Slight redness of ears (Day 7 - 10), Slight redness of ears, TS (Day 11) |
|  | **251** | NSO (Day 0 -9), Slight redness of ears (Day 10), Slight redness of ears, TS (Day 11) |
|  | **252** | NSO (Day 0 -9), Slight redness of ears (Day 10), Hunched and slight redness of  ears, TS (Day 11) |

### Table 4. Absolute and Relative Body Weights (Pilot Study)

| **Animal Number** | **Absolute (g)** | | | **Relative (% of Day 0)** | | |
| --- | --- | --- | --- | --- | --- | --- |
|  | **Day 0** | **Day 7** | **NX** | **Day 0** | **Day 7** | **NX** |
| **101** | 32.3 | 32.3 | 31.8 | 100.0 | 100.0 | 98.5 |
| **102** | 36.0 | 34.8 | 34.2 | 100.0 | 96.7 | 95.0 |
| **151** | 23.4 | 20.4 | 16.5 | 100.0 | 87.2 | 70.5 |
| **152** | 23.2 | 20.8 | 17.2 | 100.0 | 89.7 | 74.1 |
| **MEAN** | **28.7** | **27.1** | **24.9** | **100.0** | **93.4** | **84.5** |
| **SD** | **6.44** | **7.55** | **9.38** | **0.00** | **5.97** | **14.24** |
| **201** | 28.8 | 25.0 | 23.3 | 100.0 | 86.8 | 80.9 |
| **202** | 28.5 | 25.9 | 23.3 | 100.0 | 90.9 | 81.8 |
| **251** | 22.9 | 21.4 | 18.4 | 100.0 | 93.4 | 80.3 |
| **252** | 21.8 | 19.8 | 17.3 | 100.0 | 90.8 | 79.4 |
| **MEAN** | **25.5** | **23.0** | **20.6** | **100.0** | **90.5** | **80.6** |
| **SD** | **3.67** | **2.90** | **3.18** | **0.00** | **2.74** | **1.00** |

### Table 5. DRAIZE Scores (Pilot Study)

| **Animal Number** | **Day 0** | | **Day 1** | | **Day 2** | | **Day 3** | | **Day 4** | | **Day 7** | | **Day 8** | | **Day 9** | | **Day 10** | | **Day 11** | |
| --- | --- | --- | --- | --- | --- | --- | --- | --- | --- | --- | --- | --- | --- | --- | --- | --- | --- | --- | --- | --- |
|  | **Ery** | **Ede** | **Ery** | **Ede** | **Ery** | **Ede** | **Ery** | **Ede** | **Ery** | **Ede** | **Ery** | **Ede** | **Ery** | **Ede** | **Ery** | **Ede** | **Ery** | **Ede** | **Ery** | **Ede** |
| **101 R** | 0 | 0 | 0 | 0 | 0 | 0 | 0 | 0 | 0 | 0 | 1 | 1 | 1 | 1 | 0 | 1 | 0 | 1 | 1 | 1 |
| **101 L** | 0 | 0 | 0 | 0 | 0 | 0 | 0 | 0 | 0 | 0 | 1 | 1 | 1 | 2 | 1 | 1 | 1 | 2 | 1 | 2 |
| **102 R** | 0 | 0 | 0 | 0 | 0 | 0 | 0 | 0 | 0 | 0 | 1 | 1 | 1 | 1 | 1 | 2 | 1 | 2 | 2 | 2 |
| **102 L** | 0 | 0 | 0 | 0 | 0 | 0 | 0 | 0 | 0 | 0 | 1 | 1 | 1 | 1 | 1 | 1 | 1 | 1 | 2 | 1 |
| **151 R** | 0 | 0 | 0 | 0 | 0 | 0 | 1 | 1 | 1 | 1 | 1 | 2 | 2 | 3 | 2 | 3 | 3 | 3 | 4 | 3 |
| **151 L** | 0 | 0 | 0 | 0 | 0 | 0 | 1 | 1 | 1 | 1 | 2 | 2 | 2 | 2 | 2 | 2 | 2 | 2 | 4 | 3 |
| **152 R** | 0 | 0 | 0 | 0 | 0 | 0 | 0 | 0 | 0 | 1 | 1 | 2 | 1 | 2 | 1 | 3 | 2 | 3 | 3 | 3 |
| **152 L** | 0 | 0 | 0 | 0 | 0 | 0 | 0 | 0 | 0 | 1 | 1 | 2 | 1 | 2 | 1 | 2 | 2 | 2 | 3 | 3 |
| **MEAN** | **0.00** | **0.00** | **0.00** | **0.00** | **0.00** | **0.00** | **0.25** | **0.25** | **0.25** | **0.50** | **1.13** | **1.50** | **1.25** | **1.75** | **1.13** | **1.88** | **1.50** | **2.00** | **2.50** | **2.25** |
| **SD** | **0.00** | **0.00** | **0.00** | **0.00** | **0.00** | **0.00** | **0.46** | **0.46** | **0.46** | **0.53** | **0.35** | **0.53** | **0.46** | **0.71** | **0.64** | **0.83** | **0.93** | **0.76** | **1.20** | **0.89** |
| **201 R** | 0 | 0 | 0 | 0 | 0 | 0 | 0 | 1 | 0 | 1 | 0 | 0 | 1 | 0 | 1 | 1 | 1 | 1 | 1 | 2 |
| **201 L** | 0 | 0 | 0 | 0 | 0 | 0 | 0 | 1 | 0 | 1 | 0 | 0 | 1 | 0 | 1 | 1 | 1 | 2 | 1 | 2 |
| **202 R** | 0 | 0 | 0 | 0 | 0 | 0 | 1 | 0 | 1 | 0 | 0 | 0 | 1 | 0 | 1 | 0 | 1 | 1 | 1 | 1 |
| **202 L** | 0 | 0 | 0 | 0 | 0 | 0 | 0 | 0 | 1 | 0 | 0 | 0 | 1 | 0 | 1 | 0 | 1 | 1 | 1 | 1 |
| **251 R** | 0 | 0 | 0 | 0 | 0 | 0 | 0 | 0 | 0 | 0 | 0 | 0 | 0 | 1 | 0 | 0 | 1 | 1 | 1 | 2 |
| **MEAN1** | **0.00** | **0.00** | **0.00** | **0.00** | **0.00** | **0.00** | **0.25** | **0.50** | **0.50** | **0.50** | **0.00** | **0.00** | **1.00** | **0.00** | **1.00** | **0.50** | **1.00** | **1.25** | **1.00** | **1.50** |
| **SD** | **0.00** | **0.00** | **0.00** | **0.00** | **0.00** | **0.00** | **0.50** | **0.58** | **0.58** | **0.58** | **0.00** | **0.00** | **0.00** | **0.00** | **0.00** | **0.58** | **0.00** | **0.50** | **0.00** | **0.58** |
| **251 L** | 0 | 0 | 0 | 0 | 0 | 0 | 0 | 0 | 0 | 0 | 0 | 0 | 0 | 1 | 0 | 1 | 1 | 2 | 1 | 2 |
| **252 R** | 0 | 0 | 0 | 0 | 0 | 0 | 0 | 0 | 0 | 0 | 0 | 0 | 0 | 0 | 0 | 0 | 1 | 1 | 1 | 1 |
| **252 L** | 0 | 0 | 0 | 0 | 0 | 0 | 0 | 0 | 0 | 0 | 0 | 0 | 0 | 0 | 0 | 0 | 1 | 1 | 1 | 1 |
| **MEAN2** | **0.00** | **0.00** | **0.00** | **0.00** | **0.00** | **0.00** | **0.00** | **0.00** | **0.00** | **0.00** | **0.00** | **0.00** | **0.00** | **0.50** | **0.00** | **0.25** | **1.00** | **1.25** | **1.00** | **1.50** |
| **SD** | **0.00** | **0.00** | **0.00** | **0.00** | **0.00** | **0.00** | **0.00** | **0.00** | **0.00** | **0.00** | **0.00** | **0.00** | **0.00** | **0.58** | **0.00** | **0.50** | **0.00** | **0.50** | **0.00** | **0.58** |
| **Total MEAN** | **0.00** | **0.00** | **0.00** | **0.00** | **0.00** | **0.00** | **0.13** | **0.25** | **0.25** | **0.25** | **0.00** | **0.00** | **0.50** | **0.25** | **0.50** | **0.38** | **1.00** | **1.25** | **1.00** | **1.50** |
| **SD** | **0.00** | **0.00** | **0.00** | **0.00** | **0.00** | **0.00** | **0.35** | **0.46** | **0.46** | **0.46** | **0.00** | **0.00** | **0.53** | **0.46** | **0.53** | **0.52** | **0.00** | **0.46** | **0.00** | **0.53** |

1=oral treatment, 2=subcutaneous treatment, Ery=erythema, Ede-edema

### Table 6. Ear Thickness Measures (Pilot Study)

| **Animal Number** | **Raw (mm)** | | | | | | | | | | **Relative (%)** | | | | | | | | | |
| --- | --- | --- | --- | --- | --- | --- | --- | --- | --- | --- | --- | --- | --- | --- | --- | --- | --- | --- | --- | --- |
|  | **Day 0** | **Day 1** | **Day 2** | **Day 3** | **Day 4** | **Day 7** | **Day 8** | **Day 9** | **Day 10** | **Day 11** | **Day 0** | **Day 1** | **Day 2** | **Day 3** | **Day 4** | **Day 7** | **Day 8** | **Day 9** | **Day 10** | **Day 11** |
| **101 R** | 0.31 | 0.29 | 0.31 | 0.34 | 0.43 | 0.44 | 0.52 | 0.52 | 0.44 | 0.55 | 100.0 | 93.5 | 100.0 | 109.7 | 138.7 | 141.9 | 167.7 | 167.7 | 141.9 | 177.4 |
| **101 L** | 0.28 | 0.28 | 0.31 | 0.34 | 0.47 | 0.56 | 0.68 | 0.68 | 0.79 | 0.71 | 100.0 | 100.0 | 110.7 | 121.4 | 167.9 | 200.0 | 242.9 | 242.9 | 282.1 | 253.6 |
| **102 R** | 0.25 | 0.27 | 0.33 | 0.31 | 0.38 | 0.60 | 0.57 | 0.77 | 0.80 | 0.67 | 100.0 | 108.0 | 132.0 | 124.0 | 152.0 | 240.0 | 228.0 | 308.0 | 320.0 | 268.0 |
| **102 L** | 0.26 | 0.27 | 0.34 | 0.32 | 0.39 | 0.52 | 0.59 | 0.53 | 0.61 | 0.61 | 100.0 | 103.8 | 130.8 | 123.1 | 150.0 | 200.0 | 226.9 | 203.8 | 234.6 | 234.6 |
| **151 R** | 0.28 | 0.29 | 0.33 | 0.38 | 0.38 | 0.82 | 0.92 | 1.05 | 1.20 | 1.14 | 100.0 | 103.6 | 117.9 | 135.7 | 135.7 | 292.9 | 328.6 | 375.0 | 428.6 | 407.1 |
| **151 L** | 0.32 | 0.31 | 0.38 | 0.44 | 0.42 | 0.67 | 0.76 | 0.76 | 0.76 | 0.83 | 100.0 | 96.9 | 118.8 | 137.5 | 131.3 | 209.4 | 237.5 | 237.5 | 237.5 | 259.4 |
| **152 R** | 0.26 | 0.29 | 0.33 | 0.37 | 0.43 | 0.76 | 0.79 | 0.96 | 0.99 | 1.09 | 100.0 | 111.5 | 126.9 | 142.3 | 165.4 | 292.3 | 303.8 | 369.2 | 380.8 | 419.2 |
| **152 L** | 0.25 | 0.29 | 0.29 | 0.31 | 0.49 | 0.61 | 0.70 | 0.76 | 0.82 | 0.89 | 100.0 | 116.0 | 116.0 | 124.0 | 196.0 | 244.0 | 280.0 | 304.0 | 328.0 | 356.0 |
| **MEAN** | **0.28** | **0.29** | **0.33** | **0.35** | **0.42** | **0.62** | **0.69** | **0.75** | **0.80** | **0.81** | **100.0** | **104.2** | **119.1** | **127.2** | **154.6** | **227.6** | **251.9** | **276.0** | **294.2** | **296.9** |
| **SD** | **0.027** | **0.013** | **0.027** | **0.044** | **0.041** | **0.125** | **0.132** | **0.185** | **0.228** | **0.218** | **0.00** | **7.49** | **10.76** | **10.59** | **21.34** | **50.80** | **50.62** | **75.41** | **90.60** | **86.92** |
| **201 R** | 0.24 | 0.26 | 0.25 | 0.48 | 0.34 | 0.40 | 0.44 | 0.51 | 0.58 | 0.60 | 100.0 | 108.3 | 104.2 | 200.0 | 141.7 | 166.7 | 183.3 | 212.5 | 241.7 | 250.0 |
| **201 L** | 0.24 | 0.28 | 0.22 | 0.43 | 0.30 | 0.40 | 0.45 | 0.55 | 0.63 | 0.67 | 100.0 | 116.7 | 91.7 | 179.2 | 125.0 | 166.7 | 187.5 | 229.2 | 262.5 | 279.2 |
| **202 R** | 0.24 | 0.24 | 0.26 | 0.35 | 0.29 | 0.37 | 0.41 | 0.44 | 0.50 | 0.54 | 100.0 | 100.0 | 108.3 | 145.8 | 120.8 | 154.2 | 170.8 | 183.3 | 208.3 | 225.0 |
| **202 L** | 0.21 | 0.24 | 0.26 | 0.36 | 0.30 | 0.38 | 0.39 | 0.44 | 0.46 | 0.53 | 100.0 | 114.3 | 123.8 | 171.4 | 142.9 | 181.0 | 185.7 | 209.5 | 219.0 | 252.4 |
| **MEAN^1^** | **0.23** | **0.26** | **0.25** | **0.41** | **0.31** | **0.39** | **0.42** | **0.49** | **0.54** | **0.59** | **100.0** | **109.8** | **107.0** | **174.1** | **132.6** | **167.1** | **181.8** | **208.6** | **232.9** | **251.6** |
| **SD** | **0.015** | **0.019** | **0.019** | **0.061** | **0.022** | **0.015** | **0.028** | **0.054** | **0.077** | **0.065** | **0.00** | **7.43** | **13.26** | **22.38** | **11.31** | **10.95** | **7.54** | **18.95** | **24.14** | **22.14** |
| **251 R** | 0.20 | 0.20 | 0.24 | 0.27 | 0.24 | 0.39 | 0.40 | 0.46 | 0.49 | 0.60 | 100.0 | 100.0 | 120.0 | 135.0 | 120.0 | 195.0 | 200.0 | 230.0 | 245.0 | 300.0 |
| **251 L** | 0.19 | 0.23 | 0.22 | 0.23 | 0.25 | 0.38 | 0.46 | 0.52 | 0.60 | 0.66 | 100.0 | 121.1 | 115.8 | 121.1 | 131.6 | 200.0 | 242.1 | 273.7 | 315.8 | 347.4 |
| **252 R** | 0.20 | 0.24 | 0.22 | 0.29 | 0.28 | 0.37 | 0.41 | 0.48 | 0.55 | 0.51 | 100.0 | 120.0 | 110.0 | 145.0 | 140.0 | 185.0 | 205.0 | 240.0 | 275.0 | 255.0 |
| **252 L** | 0.19 | 0.20 | 0.21 | 0.27 | 0.27 | 0.36 | 0.42 | 0.43 | 0.54 | 0.56 | 100.0 | 105.3 | 110.5 | 142.1 | 142.1 | 189.5 | 221.1 | 226.3 | 284.2 | 294.7 |
| **MEAN^2^** | **0.20** | **0.22** | **0.22** | **0.27** | **0.26** | **0.38** | **0.42** | **0.47** | **0.55** | **0.58** | **100.0** | **111.6** | **114.1** | **135.8** | **133.4** | **192.4** | **217.0** | **242.5** | **280.0** | **299.3** |
| **SD** | **0.006** | **0.021** | **0.013** | **0.025** | **0.018** | **0.013** | **0.026** | **0.038** | **0.045** | **0.063** | **0.00** | **10.56** | **4.73** | **10.69** | **10.04** | **6.53** | **18.97** | **21.58** | **29.15** | **37.83** |
| **TOTAL MEAN** | **0.21** | **0.24** | **0.24** | **0.34** | **0.28** | **0.38** | **0.42** | **0.48** | **0.54** | **0.58** | **100.0** | **110.7** | **110.5** | **154.9** | **133.0** | **179.7** | **199.4** | **225.6** | **256.4** | **275.5** |
| **SD** | **0.023** | **0.027** | **0.020** | **0.087** | **0.032** | **0.015** | **0.025** | **0.044** | **0.058** | **0.059** | **0.00** | **8.50** | **9.97** | **26.14** | **9.91** | **15.87** | **23.08** | **26.10** | **35.33** | **38.37** |

1=oral treatment, 2= subcutaneous treatment


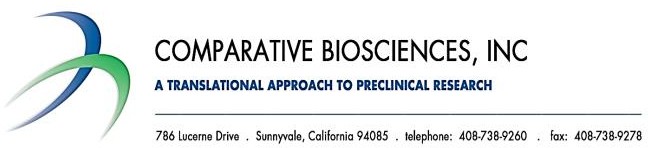


### Table 7. Histology Scores (Pilot Study)

| **Group** | **Animal Number** | **Right Ear** | **Left Ear** | **Histopathologic Findings** |
| --- | --- | --- | --- | --- |
| **1**  **NO**  **Treatment** | **101** | 4 | 4 | Severe acute suppurative inflammation composed of neutrophils and fewer lymphoid cells with edema and hyperemia, consistent in all animals. There is marked hyperplasia of the epithelium with multifocal ulceration and loss of the epithelium. |
|  | **102** | 3 | 3 |  |
|  | **151** | 4 | 4 |  |
|  | **152** | 4 | 4 |  |
|  | **MEAN** | **3.8** | **3.8** |  |
|  | **SD** | **0.50** | **0.50** |  |
| **2**  **Oral Dexamethasone (5mg/kg)** | **201** | 4 | 4 |  |
|  | **202** | 4 | 4 |  |
|  | **MEAN** | **4.0** | **4.0** |  |
|  | **SD** | **0.00** | **0.00** |  |
| **2**  **SC**  **Dexamethasone (5mg/kg)** | **251** | 3 | 3 | Moderate suppurative inflammation composed of neutrophils and fewer lymphoid cells with edema and hyperemia, consistent in all animals. There is moderate hyperplasia of the epithelium with multifocal ulceration and  loss of the epithelium. |
|  | **252** | 3 | 3 |  |
|  | **MEAN** | **3.0** | **3.0** |  |
|  | **SD** | **0.00** | **0.00** |  |

SC=subcutaneous

# EFFICACY STUDY DATA

### Table 8. Clinical Observations (Efficacy Study)

| **Group** | **Animal Number** | **Clinical Observation (days observed)** |
| --- | --- | --- |
| 1 | 101 | NSO (Day 0-8), NSO, TS (Day 9) |
|  | 151 | NSO (Day 0-8), NSO, TS (Day 9) |
| 2 | 201 | NSO (Day 0-4), Slight redness on Right ear (Day 5), Slight redness on both ears (Day 6), Slight redness on both ears, right ear swollen (Day 7), Redness on both ears, right ear swollen (Day 8), Redness on both ears, both ears swollen, TS (Day 9) |
|  | 202 | NSO (Day 0-4), Slight redness on both ears (Day 5-8), Slight redness on both ears, TS (Day 9) |
|  | 251 | NSO (Day 0-5), Slight redness on both ears (Day 6), Slight redness on both ears, right ear swollen (Day 7), Slight redness on both ears, both ears swollen (Day 8), Slight redness on both ears, both ears swollen, TS (Day 9) |
|  | 252 | NSO (Day 0-4), Slight redness on Right ear (Day 5), Slight redness on both ears (Day 6), Slight redness on both  ears, Left ear swollen (Day 7), Slight redness on both ears, right ear swollen (Day 8), Slight redness on both ears, both ears swollen, TS (Day 9) |
| 3 | 301 | Slight redness on tip of left ear (Day 0-4), Slight redness on both ears (Day 5-8), Slight redness on both ears, TS (Day 9) |
|  | 302 | NSO (Day 0-4), Slight redness on left ear (Day 5), Slight redness on both ears (Day 6-7), Slight redness on both ears, right ear swollen (Day 8), Slight redness on both ears, both ears swollen, TS (Day 9) |
|  | 351 | NSO (Day 0-4), Slight redness on both ears (Day 5-6), Slight redness on both ears, Left ear swollen (Day 7-8), Slight redness on both ears, both ears swollen, TS (Day 9) |
|  | 352 | NSO (Day 0-5), Slight redness on left ear (Day 6), Slight redness on both ears (Day 7-8), Slight redness both ears, both ears swollen, TS (Day 9) |
| 4 | 401 | NSO (Day 0-7), Slight redness on right ear (Day 8), Rough coat, slight redness both ears, TS (Day 9) |
|  | 402 | NSO (Day 0-6), Slight redness on both ears (Day 7-8), Slight redness on both ears, both ears swollen, TS (Day 9) |
|  | 403 | NSO (Day 0-4), Red spots on left ear (Day 5), NSO (Day 6), Slight redness on both ears (Day 7-8), Slight redness on both ears, TS (Day 9) |
|  | 451 | NSO (Day 0), Small red spots on left ear (Day 1-5), Slight redness on left ear (Day 6), Slight redness on both ears (Day 7-8), Slight redness on both ears, TS (Day 9) |
|  | 452 | NSO (Day 0-5), Slight redness on both ears (Day 6-7), Slight redness on both ears, right ear swollen (Day 8), Slight redness on both ears, both ears swollen (Day 9) |
|  | 453 | NSO (Day 0-4), Slight redness on left ear (Day 5), Slight redness on both ears (Day 6-8), Redness on left ear, slight redness on right ear, TS (Day 9) |
| 5 | 501 | NSO (Day 0-5), Slight redness both ears (Day 6), NSO (Day 7-8), NSO, TS (Day 9) |
|  | 502 | NSO (Day 0-5), Slight redness on right ear (Day 6), NSO (Day 7-8), Slight redness on left ear, TS (Day 9) |
|  | 503 | NSO (Day 0-4), Slight redness on left ear (Day 5), Slight redness on both ears (Day 6-8), Slight redness on both ears, TS (Day 9) |
|  | 551 | NSO (Day 0-4), Slight redness on both ears (Day 5-6), NSO (Day 7), Slight redness on both ears (Day 8), Slight redness on both ears, TS (Day 9) |
|  | 552 | NSO (Day 0-5), Slight redness on left ear (Day 6), NSO (Day 7-8), NSO, TS (Day 9) |
|  | 553 | NSO (Day 0-7), Slight redness on both ears (Day 8), Slight redness on both ears, TS (Day 9) |
| 6 | 601 | NSO (Day 0-8), NSO, TS (Day 9) |
|  | 602 | NSO (Day 0-8), NSO, TS (Day 9) |
|  | 603 | NSO (Day 0-8), NSO, TS (Day 9) |
|  | 651 | NSO (Day 0-8), NSO, TS (Day 9) |
|  | 652 | NSO (Day 0-8), NSO, TS (Day 9) |
|  | 653 | NSO (Day 0-8), NSO, TS (Day 9) |
| 7 | 701 | NSO (Day 0-5), Bite marks on tail (Day 6-7), Bite marks on tail healing (Day 8), Bite marks on tail healing, TS (Day 9) |
|  | 702 | NSO (Day 0-4), Bite marks on tail (Day 5-8), Bite marks on tail healing, TS (Day 9) |
|  | 703 | Rough coat (Day 0), NSO (Day 1-8), NSO, TS (Day 9) |
|  | 751 | NSO (Day 0-8), NSO, TS (Day 9) |
|  | 752 | NSO (Day 0-8), NSO, TS (Day 9) |
|  | 753 | NSO (Day 0-8), NSO, TS (Day 9) |

NSO=no abnormal observations, TS=terminal sacrifice

### Table 9. Absolute and Relative Body Weights (Efficacy Study)

| **Animal Number** | **Absolute (g)** | | | **Relative (% of Day 0)** | | |
| --- | --- | --- | --- | --- | --- | --- |
|  | **Day 0** | **Day 7** | **NX** | **Day 0** | **Day 7** | **NX** |
| **101** | 21.2 | 22.4 | 22.8 | 100.0 | 105.7 | 107.5 |
| **151** | 17.9 | 18.7 | 19.0 | 100.0 | 104.5 | 106.1 |
| **Mean** | **19.6** | **20.6** | **20.9** | **100.0** | **105.1** | **106.8** |
| **SD** | **2.3** | **2.6** | **2.7** | **0.0** | **0.8** | **1.0** |
| **201** | 19.5 | 19.1 | 17.0 | 100.0 | 97.9 | 87.2 |
| **202** | 22.1 | 23.2 | 21.6 | 100.0 | 105.0 | 97.7 |
| **251** | 16.0 | 16.4 | 14.8 | 100.0 | 102.5 | 92.5 |
| **252** | 18.8 | 18.8 | 18.3 | 100.0 | 100.0 | 97.3 |
| **Mean** | **19.1** | **19.4** | **17.9** | **100.0** | **101.4** | **93.7** |
| **SD** | **2.5** | **2.8** | **2.8** | **0.0** | **3.0** | **5.0** |
| **301** | 20.3 | 18.1 | 16.4 | 100.0 | 89.2 | 80.8 |
| **302** | 22.1 | 22.2 | 20.9 | 100.0 | 100.5 | 94.6 |
| **351** | 16.4 | 15.5 | 14.2 | 100.0 | 94.5 | 86.6 |
| **352** | 18.8 | 17.5 | 17.1 | 100.0 | 93.1 | 91.0 |
| **Mean** | **19.4** | **18.3** | **17.2** | **100.0** | **94.3** | **88.2** |
| **SD** | **2.4** | **2.8** | **2.8** | **0.0** | **4.7** | **5.9** |
| **401** | 21.1 | 20.4 | 19.3 | 100.0 | 96.7 | 91.5 |
| **402** | 22.1 | 21.2 | 20.4 | 100.0 | 95.9 | 92.3 |
| **403** | 22.5 | 22.0 | 21.1 | 100.0 | 97.8 | 93.8 |
| **451** | 18.6 | 18.0 | 15.8 | 100.0 | 96.8 | 84.9 |
| **452** | 17.0 | 15.4 | 14.2 | 100.0 | 90.6 | 83.5 |
| **453** | 16.6 | 15.4 | 14.2 | 100.0 | 92.8 | 85.5 |
| **Mean** | **19.7** | **18.7** | **17.5** | **100.0** | **95.1** | **88.6** |
| **SD** | **2.6** | **2.9** | **3.1** | **0.0** | **2.8** | **4.4** |
| **501** | 22.2 | 21.2 | 20.9 | 100.0 | 95.5 | 94.1 |
| **502** | 21.3 | 20.8 | 20.4 | 100.0 | 97.7 | 95.8 |
| **503** | 22.2 | 21.3 | 21.2 | 100.0 | 95.9 | 95.5 |
| **551** | 16.9 | 15.0 | 14.3 | 100.0 | 88.8 | 84.6 |
| **552** | 18.4 | 16.3 | 15.8 | 100.0 | 88.6 | 85.9 |
| **553** | 17.5 | 16.1 | 16.0 | 100.0 | 92.0 | 91.4 |
| **Mean** | **19.8** | **18.5** | **18.1** | **100.0** | **93.1** | **91.2** |
| **SD** | **2.4** | **2.9** | **3.1** | **0.0** | **3.9** | **4.9** |
| **601** | 22.2 | 21.1 | 20.8 | 100.0 | 95.0 | 93.7 |
| **602** | 21.0 | 15.9 | 15.5 | 100.0 | 75.7 | 73.8 |
| **603** | 22.3 | 21.1 | 21.0 | 100.0 | 94.6 | 94.2 |
| **651** | 17.8 | 17.0 | 17.4 | 100.0 | 95.5 | 97.8 |
| **652** | 16.6 | 16.2 | 16.2 | 100.0 | 97.6 | 97.6 |
| **653** | 17.5 | 16.9 | 16.8 | 100.0 | 96.6 | 96.0 |
| **Mean** | **19.6** | **18.0** | **18.0** | **100.0** | **92.5** | **92.2** |
| **SD** | **2.6** | **2.4** | **2.4** | **0.0** | **8.3** | **9.2** |
| **701** | 21.3 | 18.9 | 17.0 | 100.0 | 88.7 | 79.8 |
| **702** | 21.6 | 19.3 | 17.2 | 100.0 | 89.4 | 79.6 |
| **703** | 21.4 | 18.2 | 16.8 | 100.0 | 85.0 | 78.5 |
| **751** | 18.2 | 14.4 | 13.8 | 100.0 | 79.1 | 75.8 |
| **752** | 17.2 | 14.8 | 13.1 | 100.0 | 86.0 | 76.2 |
| **753** | 17.3 | 14.0 | 12.9 | 100.0 | 80.9 | 74.6 |
| **Mean** | **19.5** | **16.6** | **15.1** | **100.0** | **84.9** | **77.4** |
| **SD** | **2.1** | **2.4** | **2.1** | **0.0** | **4.1** | **2.2** |

NX=necropsy


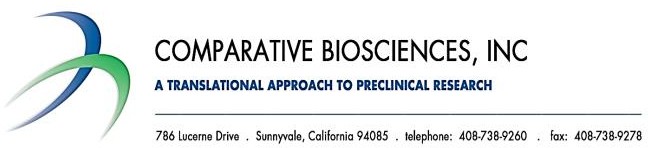


### Table 10. Draize Scores (Efficacy Study)

| **Animal Number** | **Day 0** | | **Day 1** | | **Day 2** | | **Day 5** | | **Day 6** | | **Day 7** | | **Day 8** | | **Day 9** | |
| --- | --- | --- | --- | --- | --- | --- | --- | --- | --- | --- | --- | --- | --- | --- | --- | --- |
|  | **Ery** | **Ede** | **Ery** | **Ede** | **Ery** | **Ede** | **Ery** | **Ede** | **Ery** | **Ede** | **Ery** | **Ede** | **Ery** | **Ede** | **Ery** | **Ede** |
| **101 R** | 0 | 0 | 0 | 0 | 0 | 0 | 0 | 0 | 0 | 0 | 0 | 0 | 0 | 0 | 0 | 0 |
| **101 L** | 0 | 0 | 0 | 0 | 0 | 0 | 0 | 0 | 0 | 0 | 0 | 0 | 0 | 0 | 0 | 0 |
| **151 R** | 0 | 0 | 0 | 0 | 0 | 0 | 0 | 0 | 0 | 0 | 0 | 0 | 0 | 0 | 0 | 0 |
| **151 L** | 0 | 0 | 0 | 0 | 0 | 0 | 0 | 0 | 0 | 0 | 0 | 0 | 0 | 0 | 0 | 0 |
| **MEAN** | **0.00** | **0.00** | **0.00** | **0.00** | **0.00** | **0.00** | **0.00** | **0.00** | **0.00** | **0.00** | **0.00** | **0.00** | **0.00** | **0.00** | **0.00** | **0.00** |
| **SD** | **0.00** | **0.00** | **0.00** | **0.00** | **0.00** | **0.00** | **0.00** | **0.00** | **0.00** | **0.00** | **0.00** | **0.00** | **0.00** | **0.00** | **0.00** | **0.00** |
| **201 R** | 0 | 0 | 0 | 0 | 0 | 0 | 1 | 1 | 1 | 2 | 1 | 2 | 1 | 2 | 1 | 2 |
| **201 L** | 0 | 0 | 0 | 0 | 0 | 0 | 0 | 1 | 1 | 1 | 1 | 1 | 1 | 2 | 1 | 2 |
| **202 R** | 0 | 0 | 0 | 0 | 0 | 0 | 1 | 1 | 1 | 1 | 1 | 1 | 1 | 1 | 1 | 1 |
| **202 L** | 0 | 0 | 0 | 0 | 0 | 0 | 1 | 1 | 1 | 1 | 1 | 1 | 1 | 1 | 1 | 1 |
| **251 R** | 0 | 0 | 0 | 0 | 0 | 0 | 0 | 1 | 1 | 1 | 1 | 2 | 1 | 2 | 1 | 2 |
| **251 L** | 0 | 0 | 0 | 0 | 0 | 0 | 0 | 1 | 1 | 1 | 1 | 1 | 1 | 2 | 1 | 2 |
| **252 R** | 0 | 0 | 0 | 0 | 0 | 0 | 1 | 1 | 1 | 1 | 1 | 1 | 1 | 2 | 1 | 2 |
| **252 L** | 0 | 0 | 0 | 0 | 0 | 0 | 0 | 1 | 1 | 1 | 1 | 2 | 1 | 2 | 1 | 2 |
| **MEAN** | **0.00** | **0.00** | **0.00** | **0.00** | **0.00** | **0.00** | **0.50** | **1.00** | **1.00** | **1.13** | **1.00** | **1.38** | **1.00** | **1.75** | **1.00** | **1.75** |
| **SD** | **0.00** | **0.00** | **0.00** | **0.00** | **0.00** | **0.00** | **0.53** | **0.00** | **0.00** | **0.35** | **0.00** | **0.52** | **0.00** | **0.46** | **0.00** | **0.46** |
| **301 R** | 0 | 0 | 0 | 0 | 0 | 0 | 1 | 1 | 1 | 1 | 1 | 1 | 1 | 1 | 1 | 1 |
| **301 L** | 0 | 0 | 0 | 0 | 0 | 0 | 1 | 1 | 1 | 1 | 1 | 1 | 1 | 1 | 1 | 1 |
| **302 R** | 0 | 0 | 0 | 0 | 0 | 0 | 0 | 1 | 1 | 1 | 1 | 1 | 1 | 2 | 1 | 2 |
| **302 L** | 0 | 0 | 0 | 0 | 0 | 0 | 1 | 1 | 1 | 1 | 1 | 1 | 1 | 1 | 1 | 2 |
| **351 R** | 0 | 0 | 0 | 0 | 0 | 0 | 1 | 1 | 1 | 1 | 1 | 1 | 1 | 2 | 2 | 2 |
| **351 L** | 0 | 0 | 0 | 0 | 0 | 0 | 1 | 1 | 1 | 2 | 1 | 2 | 1 | 2 | 1 | 2 |
| **352 R** | 0 | 0 | 0 | 0 | 0 | 0 | 0 | 1 | 1 | 2 | 1 | 1 | 1 | 1 | 1 | 2 |
| **352 L** | 0 | 0 | 0 | 0 | 0 | 0 | 0 | 1 | 1 | 1 | 1 | 1 | 1 | 1 | 1 | 2 |
| **MEAN** | **0.00** | **0.00** | **0.00** | **0.00** | **0.00** | **0.00** | **0.63** | **1.00** | **1.00** | **1.25** | **1.00** | **1.13** | **1.00** | **1.38** | **1.13** | **1.75** |
| **SD** | **0.00** | **0.00** | **0.00** | **0.00** | **0.00** | **0.00** | **0.52** | **0.00** | **0.00** | **0.46** | **0.00** | **0.35** | **0.00** | **0.52** | **0.35** | **0.46** |
| **401 R** | 0 | 0 | 0 | 0 | 0 | 0 | 0 | 1 | 0 | 1 | 0 | 1 | 1 | 1 | 1 | 1 |
| **401 L** | 0 | 0 | 0 | 0 | 0 | 0 | 0 | 1 | 0 | 1 | 0 | 1 | 0 | 1 | 1 | 1 |
| **402 R** | 0 | 0 | 0 | 0 | 0 | 0 | 0 | 1 | 0 | 1 | 1 | 1 | 1 | 1 | 1 | 2 |
| **402 L** | 0 | 0 | 0 | 0 | 0 | 0 | 0 | 1 | 0 | 1 | 1 | 1 | 1 | 1 | 1 | 2 |
| **403 R** | 0 | 0 | 0 | 0 | 0 | 0 | 0 | 1 | 0 | 1 | 1 | 1 | 1 | 1 | 1 | 1 |
| **403 L** | 0 | 0 | 0 | 0 | 0 | 0 | 1 | 1 | 0 | 1 | 1 | 1 | 1 | 1 | 1 | 1 |
| **451 R** | 0 | 0 | 0 | 0 | 0 | 0 | 0 | 1 | 0 | 1 | 1 | 1 | 1 | 1 | 1 | 1 |
| **451 L** | 0 | 0 | 0 | 0 | 0 | 0 | 1 | 1 | 1 | 1 | 1 | 1 | 1 | 1 | 1 | 1 |

| **Animal Number** | **Day 0** | | **Day 1** | | **Day 2** | | **Day 5** | | **Day 6** | | **Day 7** | | **Day 8** | | **Day 9** | |
| --- | --- | --- | --- | --- | --- | --- | --- | --- | --- | --- | --- | --- | --- | --- | --- | --- |
|  | **Ery** | **Ede** | **Ery** | **Ede** | **Ery** | **Ede** | **Ery** | **Ede** | **Ery** | **Ede** | **Ery** | **Ede** | **Ery** | **Ede** | **Ery** | **Ede** |
| **452 R** | 0 | 0 | 0 | 0 | 0 | 0 | 0 | 1 | 1 | 2 | 1 | 1 | 1 | 2 | 1 | 2 |
| **452 L** | 0 | 0 | 0 | 0 | 0 | 0 | 0 | 1 | 1 | 2 | 1 | 1 | 1 | 1 | 1 | 2 |
| **453 R** | 0 | 0 | 0 | 0 | 0 | 0 | 0 | 1 | 1 | 1 | 1 | 1 | 1 | 1 | 1 | 1 |
| **453 L** | 0 | 0 | 0 | 0 | 0 | 0 | 1 | 1 | 1 | 1 | 1 | 1 | 1 | 1 | 2 | 1 |
| **MEAN** | **0.00** | **0.00** | **0.00** | **0.00** | **0.00** | **0.00** | **0.25** | **1.00** | **0.42** | **1.17** | **0.83** | **1.00** | **0.92** | **1.08** | **1.08** | **1.33** |
| **SD** | **0.00** | **0.00** | **0.00** | **0.00** | **0.00** | **0.00** | **0.45** | **0.00** | **0.51** | **0.39** | **0.39** | **0.00** | **0.29** | **0.29** | **0.29** | **0.49** |
| **501 R** | 0 | 0 | 0 | 0 | 0 | 0 | 0 | 1 | 1 | 2 | 0 | 1 | 0 | 1 | 0 | 1 |
| **501 L** | 0 | 0 | 0 | 0 | 0 | 0 | 0 | 1 | 1 | 2 | 0 | 1 | 0 | 1 | 0 | 1 |
| **502 R** | 0 | 0 | 0 | 0 | 0 | 0 | 0 | 1 | 1 | 1 | 0 | 1 | 0 | 1 | 0 | 1 |
| **502 L** | 0 | 0 | 0 | 0 | 0 | 0 | 0 | 1 | 0 | 1 | 0 | 1 | 0 | 1 | 1 | 1 |
| **503 R** | 0 | 0 | 0 | 0 | 0 | 0 | 0 | 1 | 1 | 1 | 1 | 1 | 1 | 1 | 1 | 1 |
| **503 L** | 0 | 0 | 0 | 0 | 0 | 0 | 1 | 1 | 1 | 1 | 1 | 1 | 1 | 1 | 1 | 1 |
| **551 R** | 0 | 0 | 0 | 0 | 0 | 0 | 1 | 1 | 1 | 1 | 0 | 1 | 1 | 1 | 1 | 1 |
| **551 L** | 0 | 0 | 0 | 0 | 0 | 0 | 1 | 1 | 1 | 1 | 0 | 1 | 1 | 1 | 1 | 1 |
| **552 R** | 0 | 0 | 0 | 0 | 0 | 0 | 0 | 1 | 0 | 1 | 0 | 1 | 0 | 1 | 1 | 1 |
| **552 L** | 0 | 0 | 0 | 0 | 0 | 0 | 0 | 1 | 1 | 1 | 0 | 1 | 0 | 1 | 1 | 1 |
| **553 R** | 0 | 0 | 0 | 0 | 0 | 0 | 0 | 1 | 0 | 1 | 0 | 1 | 1 | 1 | 1 | 1 |
| **553 L** | 0 | 0 | 0 | 0 | 0 | 0 | 0 | 1 | 0 | 1 | 0 | 1 | 1 | 1 | 1 | 1 |
| **MEAN** | **0.00** | **0.00** | **0.00** | **0.00** | **0.00** | **0.00** | **0.25** | **1.00** | **0.67** | **1.17** | **0.17** | **1.00** | **0.50** | **1.00** | **0.75** | **1.00** |
| **SD** | **0.00** | **0.00** | **0.00** | **0.00** | **0.00** | **0.00** | **0.45** | **0.00** | **0.49** | **0.39** | **0.39** | **0.00** | **0.52** | **0.00** | **0.45** | **0.00** |
| **601 R** | 0 | 0 | 0 | 0 | 0 | 0 | 0 | 1 | 0 | 1 | 0 | 1 | 0 | 1 | 1 | 1 |
| **601 L** | 0 | 0 | 0 | 0 | 0 | 0 | 0 | 1 | 0 | 1 | 0 | 1 | 0 | 1 | 1 | 1 |
| **602 R** | 0 | 0 | 0 | 0 | 0 | 0 | 0 | 1 | 0 | 1 | 0 | 1 | 0 | 1 | 1 | 1 |
| **602 L** | 0 | 0 | 0 | 0 | 0 | 0 | 0 | 1 | 0 | 1 | 0 | 1 | 0 | 1 | 1 | 1 |
| **603 R** | 0 | 0 | 0 | 0 | 0 | 0 | 0 | 1 | 0 | 1 | 0 | 1 | 0 | 1 | 1 | 1 |
| **603 L** | 0 | 0 | 0 | 0 | 0 | 0 | 0 | 1 | 0 | 1 | 0 | 1 | 0 | 1 | 1 | 1 |
| **651 R** | 0 | 0 | 0 | 0 | 0 | 0 | 0 | 1 | 0 | 1 | 0 | 1 | 0 | 1 | 0 | 1 |
| **651 L** | 0 | 0 | 0 | 0 | 0 | 0 | 0 | 1 | 0 | 1 | 0 | 1 | 0 | 1 | 0 | 1 |
| **652 R** | 0 | 0 | 0 | 0 | 0 | 0 | 0 | 1 | 0 | 1 | 0 | 1 | 0 | 1 | 0 | 1 |
| **652 L** | 0 | 0 | 0 | 0 | 0 | 0 | 0 | 1 | 0 | 1 | 0 | 1 | 0 | 1 | 0 | 1 |
| **653 R** | 0 | 0 | 0 | 0 | 0 | 0 | 0 | 1 | 0 | 1 | 0 | 1 | 0 | 1 | 0 | 1 |
| **653 L** | 0 | 0 | 0 | 0 | 0 | 0 | 0 | 1 | 0 | 1 | 0 | 1 | 0 | 1 | 0 | 1 |
| **MEAN** | **0.00** | **0.00** | **0.00** | **0.00** | **0.00** | **0.00** | **0.00** | **1.00** | **0.00** | **1.00** | **0.00** | **1.00** | **0.00** | **1.00** | **0.50** | **1.00** |
| **SD** | **0.00** | **0.00** | **0.00** | **0.00** | **0.00** | **0.00** | **0.00** | **0.00** | **0.00** | **0.00** | **0.00** | **0.00** | **0.00** | **0.00** | **0.52** | **0.00** |
| **701 R** | 0 | 0 | 0 | 0 | 0 | 0 | 0 | 0 | 0 | 0 | 0 | 0 | 0 | 0 | 0 | 0 |
| **701 L** | 0 | 0 | 0 | 0 | 0 | 0 | 0 | 0 | 0 | 0 | 0 | 0 | 0 | 0 | 0 | 0 |
| **702 R** | 0 | 0 | 0 | 0 | 0 | 0 | 0 | 0 | 0 | 0 | 0 | 0 | 0 | 1 | 0 | 0 |

| **Animal Number** | **Day 0** | | **Day 1** | | **Day 2** | | **Day 5** | | **Day 6** | | **Day 7** | | **Day 8** | | **Day 9** | |
| --- | --- | --- | --- | --- | --- | --- | --- | --- | --- | --- | --- | --- | --- | --- | --- | --- |
|  | **Ery** | **Ede** | **Ery** | **Ede** | **Ery** | **Ede** | **Ery** | **Ede** | **Ery** | **Ede** | **Ery** | **Ede** | **Ery** | **Ede** | **Ery** | **Ede** |
| **702 L** | 0 | 0 | 0 | 0 | 0 | 0 | 0 | 0 | 0 | 0 | 0 | 0 | 0 | 1 | 0 | 0 |
| **703 R** | 0 | 0 | 0 | 0 | 0 | 0 | 0 | 1 | 0 | 0 | 0 | 0 | 0 | 1 | 0 | 0 |
| **703 L** | 0 | 0 | 0 | 0 | 0 | 0 | 0 | 0 | 0 | 0 | 0 | 0 | 0 | 1 | 0 | 0 |
| **751 R** | 0 | 0 | 0 | 0 | 0 | 0 | 0 | 0 | 0 | 0 | 0 | 0 | 0 | 1 | 0 | 0 |
| **751 L** | 0 | 0 | 0 | 0 | 0 | 0 | 0 | 0 | 0 | 0 | 0 | 0 | 0 | 0 | 0 | 0 |
| **752 R** | 0 | 0 | 0 | 0 | 0 | 0 | 0 | 0 | 0 | 0 | 0 | 0 | 0 | 0 | 0 | 0 |
| **752 L** | 0 | 0 | 0 | 0 | 0 | 0 | 0 | 0 | 0 | 0 | 0 | 0 | 0 | 0 | 0 | 0 |
| **753 R** | 0 | 0 | 0 | 0 | 0 | 0 | 0 | 0 | 0 | 0 | 0 | 0 | 0 | 0 | 0 | 0 |
| **753 L** | 0 | 0 | 0 | 0 | 0 | 0 | 0 | 0 | 0 | 0 | 0 | 0 | 0 | 0 | 0 | 0 |
| **MEAN** | **0.00** | **0.00** | **0.00** | **0.00** | **0.00** | **0.00** | **0.00** | **0.08** | **0.00** | **0.00** | **0.00** | **0.00** | **0.00** | **0.42** | **0.00** | **0.00** |
| **SD** | **0.00** | **0.00** | **0.00** | **0.00** | **0.00** | **0.00** | **0.00** | **0.29** | **0.00** | **0.00** | **0.00** | **0.00** | **0.00** | **0.51** | **0.00** | **0.00** |

Ery=erythema, Ede=edema


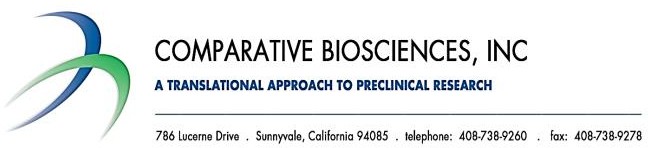


### Table 11. Ear Thickness Measures (Efficacy Study)

| **Animal Number** | **Absolute (mm)** | | | | | | | | **Relative (%)** | | | | | | | |
| --- | --- | --- | --- | --- | --- | --- | --- | --- | --- | --- | --- | --- | --- | --- | --- | --- |
|  | **Day 0** | **Day 1** | **Day 2** | **Day 5** | **Day 6** | **Day 7** | **Day 8** | **Day 9** | **Day 0** | **Day 1** | **Day 2** | **Day 5** | **Day 6** | **Day 7** | **Day 8** | **Day 9** |
| **101 R** | 0.23 | 0.24 | 0.24 | 0.25 | 0.26 | 0.24 | 0.26 | 0.27 | 100.0 | 104.3 | 104.3 | 108.7 | 113.0 | 104.3 | 113.0 | 117.4 |
| **101 L** | 0.23 | 0.22 | 0.23 | 0.23 | 0.24 | 0.23 | 0.24 | 0.27 | 100.0 | 95.7 | 100.0 | 100.0 | 104.3 | 100.0 | 104.3 | 117.4 |
| **151 R** | 0.21 | 0.22 | 0.21 | 0.22 | 0.23 | 0.23 | 0.25 | 0.22 | 100.0 | 104.8 | 100.0 | 104.8 | 109.5 | 109.5 | 119.0 | 104.8 |
| **151 L** | 0.21 | 0.21 | 0.20 | 0.22 | 0.21 | 0.23 | 0.24 | 0.23 | 100.0 | 100.0 | 95.2 | 104.8 | 100.0 | 109.5 | 114.3 | 109.5 |
| **Mean** | **0.22** | **0.22** | **0.22** | **0.23** | **0.24** | **0.23** | **0.25** | **0.25** | **100.0** | **101.2** | **99.9** | **104.6** | **106.7** | **105.8** | **112.7** | **112.3** |
| **SD** | **0.01** | **0.01** | **0.02** | **0.01** | **0.02** | **0.00** | **0.01** | **0.03** | **0.00** | **4.27** | **3.72** | **3.56** | **5.73** | **4.60** | **6.13** | **6.23** |
| **201 R** | 0.24 | 0.26 | 0.27 | 0.38 | 0.55 | 0.64 | 0.48 | 0.57 | 100.0 | 108.3 | 112.5 | 158.3 | 229.2 | 266.7 | 200.0 | 237.5 |
| **201 L** | 0.23 | 0.24 | 0.25 | 0.35 | 0.44 | 0.46 | 0.49 | 0.50 | 100.0 | 104.3 | 108.7 | 152.2 | 191.3 | 200.0 | 213.0 | 217.4 |
| **202 R** | 0.24 | 0.24 | 0.27 | 0.33 | 0.36 | 0.37 | 0.37 | 0.40 | 100.0 | 100.0 | 112.5 | 137.5 | 150.0 | 154.2 | 154.2 | 166.7 |
| **202 L** | 0.23 | 0.24 | 0.25 | 0.35 | 0.34 | 0.35 | 0.36 | 0.41 | 100.0 | 104.3 | 108.7 | 152.2 | 147.8 | 152.2 | 156.5 | 178.3 |
| **251 R** | 0.23 | 0.24 | 0.28 | 0.35 | 0.45 | 0.51 | 0.44 | 0.55 | 100.0 | 104.3 | 121.7 | 152.2 | 195.7 | 221.7 | 191.3 | 239.1 |
| **251 L** | 0.23 | 0.23 | 0.24 | 0.33 | 0.41 | 0.47 | 0.40 | 0.52 | 100.0 | 100.0 | 104.3 | 143.5 | 178.3 | 204.3 | 173.9 | 226.1 |
| **252 R** | 0.21 | 0.23 | 0.26 | 0.36 | 0.36 | 0.38 | 0.41 | 0.44 | 100.0 | 109.5 | 123.8 | 171.4 | 171.4 | 181.0 | 195.2 | 209.5 |
| **252 L** | 0.22 | 0.23 | 0.24 | 0.37 | 0.35 | 0.45 | 0.46 | 0.51 | 100.0 | 104.5 | 109.1 | 168.2 | 159.1 | 204.5 | 209.1 | 231.8 |
| **Mean** | **0.23** | **0.24** | **0.26** | **0.35** | **0.41** | **0.45** | **0.43** | **0.49** | **100.0** | **104.6** | **114.7** | **158.8** | **176.1** | **202.9** | **192.4** | **226.6** |
| **SD** | **0.01** | **0.01** | **0.01** | **0.02** | **0.07** | **0.09** | **0.05** | **0.06** | **0.00** | **3.39** | **6.76** | **11.43** | **27.26** | **37.18** | **22.67** | **27.24** |
| **301 R** | 0.20 | 0.24 | 0.25 | 0.32 | 0.36 | 0.41 | 0.41 | 0.40 | 100.0 | 120.0 | 125.0 | 160.0 | 180.0 | 205.0 | 205.0 | 200.0 |
| **301 L** | 0.21 | 0.24 | 0.25 | 0.36 | 0.36 | 0.40 | 0.41 | 0.42 | 100.0 | 114.3 | 119.0 | 171.4 | 171.4 | 190.5 | 195.2 | 200.0 |
| **302 R** | 0.24 | 0.23 | 0.27 | 0.33 | 0.38 | 0.37 | 0.41 | 0.44 | 100.0 | 95.8 | 112.5 | 137.5 | 158.3 | 154.2 | 170.8 | 183.3 |
| **302 L** | 0.24 | 0.21 | 0.25 | 0.37 | 0.38 | 0.36 | 0.42 | 0.46 | 100.0 | 87.5 | 104.2 | 154.2 | 158.3 | 150.0 | 175.0 | 191.7 |
| **351 R** | 0.25 | 0.22 | 0.25 | 0.33 | 0.35 | 0.39 | 0.46 | 0.45 | 100.0 | 88.0 | 100.0 | 132.0 | 140.0 | 156.0 | 184.0 | 180.0 |
| **351 L** | 0.25 | 0.23 | 0.25 | 0.38 | 0.45 | 0.50 | 0.52 | 0.47 | 100.0 | 92.0 | 100.0 | 152.0 | 180.0 | 200.0 | 208.0 | 188.0 |
| **352 R** | 0.24 | 0.23 | 0.24 | 0.35 | 0.45 | 0.36 | 0.42 | 0.47 | 100.0 | 95.8 | 100.0 | 145.8 | 187.5 | 150.0 | 175.0 | 195.8 |
| **352 L** | 0.23 | 0.22 | 0.25 | 0.39 | 0.40 | 0.44 | 0.40 | 0.45 | 100.0 | 95.7 | 108.7 | 169.6 | 173.9 | 191.3 | 173.9 | 195.7 |
| **Mean** | **0.23** | **0.23** | **0.25** | **0.35** | **0.39** | **0.40** | **0.43** | **0.45** | **100.0** | **92.9** | **102.2** | **149.8** | **170.4** | **174.3** | **185.2** | **189.9** |
| **SD** | **0.02** | **0.01** | **0.01** | **0.03** | **0.04** | **0.05** | **0.04** | **0.02** | **0.00** | **11.99** | **9.52** | **14.13** | **15.50** | **24.13** | **14.89** | **7.47** |
| **401 R** | 0.25 | 0.25 | 0.26 | 0.37 | 0.37 | 0.38 | 0.42 | 0.45 | 100.0 | 100.0 | 104.0 | 148.0 | 148.0 | 152.0 | 168.0 | 180.0 |
| **401 L** | 0.26 | 0.28 | 0.24 | 0.38 | 0.36 | 0.40 | 0.43 | 0.48 | 100.0 | 107.7 | 92.3 | 146.2 | 138.5 | 153.8 | 165.4 | 184.6 |
| **402 R** | 0.25 | 0.24 | 0.26 | 0.33 | 0.44 | 0.39 | 0.44 | 0.51 | 100.0 | 96.0 | 104.0 | 132.0 | 176.0 | 156.0 | 176.0 | 204.0 |
| **402 L** | 0.25 | 0.24 | 0.25 | 0.36 | 0.44 | 0.45 | 0.45 | 0.48 | 100.0 | 96.0 | 100.0 | 144.0 | 176.0 | 180.0 | 180.0 | 192.0 |
| **403 R** | 0.25 | 0.25 | 0.25 | 0.34 | 0.36 | 0.40 | 0.41 | 0.44 | 100.0 | 100.0 | 100.0 | 136.0 | 144.0 | 160.0 | 164.0 | 176.0 |
| **403 L** | 0.25 | 0.25 | 0.24 | 0.36 | 0.39 | 0.42 | 0.43 | 0.45 | 100.0 | 100.0 | 96.0 | 144.0 | 156.0 | 168.0 | 172.0 | 180.0 |
| **451 R** | 0.23 | 0.22 | 0.23 | 0.32 | 0.37 | 0.40 | 0.44 | 0.46 | 100.0 | 95.7 | 100.0 | 139.1 | 160.9 | 173.9 | 191.3 | 200.0 |
| **451 L** | 0.24 | 0.23 | 0.22 | 0.35 | 0.40 | 0.39 | 0.41 | 0.44 | 100.0 | 95.8 | 91.7 | 145.8 | 166.7 | 162.5 | 170.8 | 183.3 |
| **452 R** | 0.25 | 0.24 | 0.24 | 0.38 | 0.41 | 0.43 | 0.45 | 0.48 | 100.0 | 96.0 | 96.0 | 152.0 | 164.0 | 172.0 | 180.0 | 192.0 |
| **452 L** | 0.24 | 0.25 | 0.24 | 0.32 | 0.47 | 0.46 | 0.43 | 0.47 | 100.0 | 104.2 | 100.0 | 133.3 | 195.8 | 191.7 | 179.2 | 195.8 |
| **453 R** | 0.21 | 0.22 | 0.23 | 0.32 | 0.38 | 0.41 | 0.39 | 0.40 | 100.0 | 104.8 | 109.5 | 152.4 | 181.0 | 195.2 | 185.7 | 190.5 |
| **453 L** | 0.23 | 0.20 | 0.22 | 0.31 | 0.36 | 0.38 | 0.45 | 0.40 | 100.0 | 87.0 | 95.7 | 134.8 | 156.5 | 165.2 | 195.7 | 173.9 |
| **Mean** | **0.24** | **0.24** | **0.24** | **0.35** | **0.40** | **0.41** | **0.43** | **0.46** | **100.0** | **98.0** | **100.3** | **143.1** | **174.3** | **181.0** | **185.1** | **188.1** |
| **SD** | **0.01** | **0.02** | **0.01** | **0.03** | **0.04** | **0.03** | **0.02** | **0.03** | **0.00** | **5.47** | **5.16** | **7.10** | **16.60** | **14.08** | **9.99** | **9.54** |
| **501 R** | 0.24 | 0.28 | 0.24 | 0.39 | 0.45 | 0.37 | 0.39 | 0.46 | 100.0 | 116.7 | 100.0 | 162.5 | 187.5 | 154.2 | 162.5 | 191.7 |
| **501 L** | 0.23 | 0.24 | 0.27 | 0.36 | 0.46 | 0.38 | 0.35 | 0.41 | 100.0 | 104.3 | 117.4 | 156.5 | 200.0 | 165.2 | 152.2 | 178.3 |
| **502 R** | 0.25 | 0.25 | 0.23 | 0.36 | 0.36 | 0.36 | 0.39 | 0.43 | 100.0 | 100.0 | 92.0 | 144.0 | 144.0 | 144.0 | 156.0 | 172.0 |
| **502 L** | 0.27 | 0.23 | 0.25 | 0.34 | 0.35 | 0.36 | 0.41 | 0.42 | 100.0 | 85.2 | 92.6 | 125.9 | 129.6 | 133.3 | 151.9 | 155.6 |
| **503 R** | 0.22 | 0.25 | 0.21 | 0.32 | 0.37 | 0.39 | 0.39 | 0.42 | 100.0 | 113.6 | 95.5 | 145.5 | 168.2 | 177.3 | 177.3 | 190.9 |
| **503 L** | 0.24 | 0.23 | 0.24 | 0.39 | 0.35 | 0.35 | 0.40 | 0.40 | 100.0 | 95.8 | 100.0 | 162.5 | 145.8 | 145.8 | 166.7 | 166.7 |
| **551 R** | 0.21 | 0.21 | 0.22 | 0.31 | 0.31 | 0.32 | 0.37 | 0.39 | 100.0 | 100.0 | 104.8 | 147.6 | 147.6 | 152.4 | 176.2 | 185.7 |
| **551 L** | 0.22 | 0.20 | 0.20 | 0.30 | 0.37 | 0.34 | 0.39 | 0.42 | 100.0 | 90.9 | 90.9 | 136.4 | 168.2 | 154.5 | 177.3 | 190.9 |
| **552 R** | 0.22 | 0.21 | 0.24 | 0.30 | 0.33 | 0.32 | 0.38 | 0.37 | 100.0 | 95.5 | 109.1 | 136.4 | 150.0 | 145.5 | 172.7 | 168.2 |
| **552 L** | 0.21 | 0.20 | 0.22 | 0.29 | 0.31 | 0.32 | 0.36 | 0.38 | 100.0 | 95.2 | 104.8 | 138.1 | 147.6 | 152.4 | 171.4 | 181.0 |

| **Animal Number** | **Absolute (mm)** | | | | | | | | **Relative (%)** | | | | | | | |
| --- | --- | --- | --- | --- | --- | --- | --- | --- | --- | --- | --- | --- | --- | --- | --- | --- |
|  | **Day 0** | **Day 1** | **Day 2** | **Day 5** | **Day 6** | **Day 7** | **Day 8** | **Day 9** | **Day 0** | **Day 1** | **Day 2** | **Day 5** | **Day 6** | **Day 7** | **Day 8** | **Day 9** |
| **553 R** | 0.23 | 0.22 | 0.24 | 0.32 | 0.36 | 0.37 | 0.39 | 0.40 | 100.0 | 95.7 | 104.3 | 139.1 | 156.5 | 160.9 | 169.6 | 173.9 |
| **553 L** | 0.21 | 0.22 | 0.23 | 0.32 | 0.38 | 0.39 | 0.40 | 0.43 | 100.0 | 104.8 | 109.5 | 152.4 | 181.0 | 185.7 | 190.5 | 204.8 |
| **Mean** | **0.23** | **0.23** | **0.23** | **0.33** | **0.37** | **0.36** | **0.39** | **0.41** | **100.0** | **97.8** | **106.9** | **141.5** | **158.8** | **161.1** | **176.0** | **182.0** |
| **SD** | **0.02** | **0.02** | **0.02** | **0.03** | **0.05** | **0.03** | **0.02** | **0.02** | **0.00** | **8.98** | **8.12** | **11.28** | **20.72** | **14.61** | **11.53** | **13.62** |
| **601 R** | 0.25 | 0.26 | 0.26 | 0.34 | 0.35 | 0.33 | 0.38 | 0.42 | 100.0 | 104.0 | 104.0 | 136.0 | 140.0 | 132.0 | 152.0 | 168.0 |
| **601 L** | 0.25 | 0.23 | 0.25 | 0.36 | 0.36 | 0.41 | 0.47 | 0.46 | 100.0 | 92.0 | 100.0 | 144.0 | 144.0 | 164.0 | 188.0 | 184.0 |
| **602 R** | 0.28 | 0.25 | 0.25 | 0.35 | 0.38 | 0.36 | 0.42 | 0.44 | 100.0 | 89.3 | 89.3 | 125.0 | 135.7 | 128.6 | 150.0 | 157.1 |
| **602 L** | 0.26 | 0.24 | 0.24 | 0.36 | 0.37 | 0.32 | 0.39 | 0.43 | 100.0 | 92.3 | 92.3 | 138.5 | 142.3 | 123.1 | 150.0 | 165.4 |
| **603 R** | 0.24 | 0.24 | 0.22 | 0.33 | 0.36 | 0.33 | 0.42 | 0.44 | 100.0 | 100.0 | 91.7 | 137.5 | 150.0 | 137.5 | 175.0 | 183.3 |
| **603 L** | 0.26 | 0.24 | 0.22 | 0.32 | 0.34 | 0.33 | 0.39 | 0.43 | 100.0 | 92.3 | 84.6 | 123.1 | 130.8 | 126.9 | 150.0 | 165.4 |
| **651 R** | 0.23 | 0.23 | 0.22 | 0.33 | 0.34 | 0.30 | 0.35 | 0.39 | 100.0 | 100.0 | 95.7 | 143.5 | 147.8 | 130.4 | 152.2 | 169.6 |
| **651 L** | 0.22 | 0.21 | 0.22 | 0.31 | 0.36 | 0.33 | 0.36 | 0.42 | 100.0 | 95.5 | 100.0 | 140.9 | 163.6 | 150.0 | 163.6 | 190.9 |
| **652 R** | 0.22 | 0.23 | 0.23 | 0.31 | 0.37 | 0.33 | 0.41 | 0.43 | 100.0 | 104.5 | 104.5 | 140.9 | 168.2 | 150.0 | 186.4 | 195.5 |
| **652 L** | 0.23 | 0.21 | 0.24 | 0.32 | 0.36 | 0.34 | 0.36 | 0.40 | 100.0 | 91.3 | 104.3 | 139.1 | 156.5 | 147.8 | 156.5 | 173.9 |
| **653 R** | 0.23 | 0.21 | 0.22 | 0.32 | 0.39 | 0.34 | 0.39 | 0.41 | 100.0 | 91.3 | 95.7 | 139.1 | 169.6 | 147.8 | 169.6 | 178.3 |
| **653 L** | 0.24 | 0.22 | 0.20 | 0.31 | 0.36 | 0.35 | 0.39 | 0.42 | 100.0 | 91.7 | 83.3 | 129.2 | 150.0 | 145.8 | 162.5 | 175.0 |
| **Mean** | **0.24** | **0.23** | **0.23** | **0.33** | **0.36** | **0.34** | **0.39** | **0.42** | **100.0** | **94.7** | **97.0** | **137.1** | **161.1** | **147.9** | **168.7** | **180.7** |
| **SD** | **0.02** | **0.02** | **0.02** | **0.02** | **0.01** | **0.03** | **0.03** | **0.02** | **0.00** | **5.36** | **7.40** | **6.93** | **12.49** | **12.36** | **13.97** | **11.33** |
| **701 R** | 0.21 | 0.21 | 0.20 | 0.24 | 0.24 | 0.25 | 0.28 | 0.29 | 100.0 | 100.0 | 95.2 | 114.3 | 114.3 | 119.0 | 133.3 | 138.1 |
| **701 L** | 0.22 | 0.21 | 0.21 | 0.26 | 0.26 | 0.27 | 0.28 | 0.29 | 100.0 | 95.5 | 95.5 | 118.2 | 118.2 | 122.7 | 127.3 | 131.8 |
| **702 R** | 0.24 | 0.21 | 0.22 | 0.27 | 0.25 | 0.23 | 0.32 | 0.29 | 100.0 | 87.5 | 91.7 | 112.5 | 104.2 | 95.8 | 133.3 | 120.8 |
| **702 L** | 0.23 | 0.21 | 0.20 | 0.25 | 0.28 | 0.27 | 0.35 | 0.29 | 100.0 | 91.3 | 87.0 | 108.7 | 121.7 | 117.4 | 152.2 | 126.1 |
| **703 R** | 0.23 | 0.22 | 0.22 | 0.31 | 0.31 | 0.29 | 0.38 | 0.31 | 100.0 | 95.7 | 95.7 | 134.8 | 134.8 | 126.1 | 165.2 | 134.8 |
| **703 L** | 0.23 | 0.22 | 0.22 | 0.26 | 0.28 | 0.28 | 0.35 | 0.29 | 100.0 | 95.7 | 95.7 | 113.0 | 121.7 | 121.7 | 152.2 | 126.1 |
| **751 R** | 0.21 | 0.21 | 0.20 | 0.24 | 0.24 | 0.27 | 0.31 | 0.28 | 100.0 | 100.0 | 95.2 | 114.3 | 114.3 | 128.6 | 147.6 | 133.3 |
| **751 L** | 0.20 | 0.20 | 0.19 | 0.23 | 0.22 | 0.29 | 0.27 | 0.26 | 100.0 | 100.0 | 95.0 | 115.0 | 110.0 | 145.0 | 135.0 | 130.0 |
| **752 R** | 0.21 | 0.19 | 0.19 | 0.22 | 0.22 | 0.23 | 0.26 | 0.26 | 100.0 | 90.5 | 90.5 | 104.8 | 104.8 | 109.5 | 123.8 | 123.8 |
| **752 L** | 0.20 | 0.19 | 0.19 | 0.24 | 0.21 | 0.21 | 0.26 | 0.25 | 100.0 | 95.0 | 95.0 | 120.0 | 105.0 | 105.0 | 130.0 | 125.0 |
| **753 R** | 0.21 | 0.20 | 0.20 | 0.23 | 0.23 | 0.23 | 0.25 | 0.25 | 100.0 | 95.2 | 95.2 | 109.5 | 109.5 | 109.5 | 119.0 | 119.0 |
| **753 L** | 0.21 | 0.20 | 0.20 | 0.23 | 0.23 | 0.23 | 0.26 | 0.25 | 100.0 | 95.2 | 95.2 | 109.5 | 109.5 | 109.5 | 123.8 | 119.0 |
| **Mean** | **0.22** | **0.21** | **0.20** | **0.25** | **0.25** | **0.25** | **0.30** | **0.28** | **100.0** | **94.0** | **94.0** | **111.0** | **107.2** | **108.4** | **124.2** | **121.7** |
| **SD** | **0.01** | **0.01** | **0.01** | **0.02** | **0.03** | **0.03** | **0.04** | **0.02** | **0.00** | **3.89** | **2.75** | **7.63** | **8.99** | **12.85** | **14.19** | **6.29** |


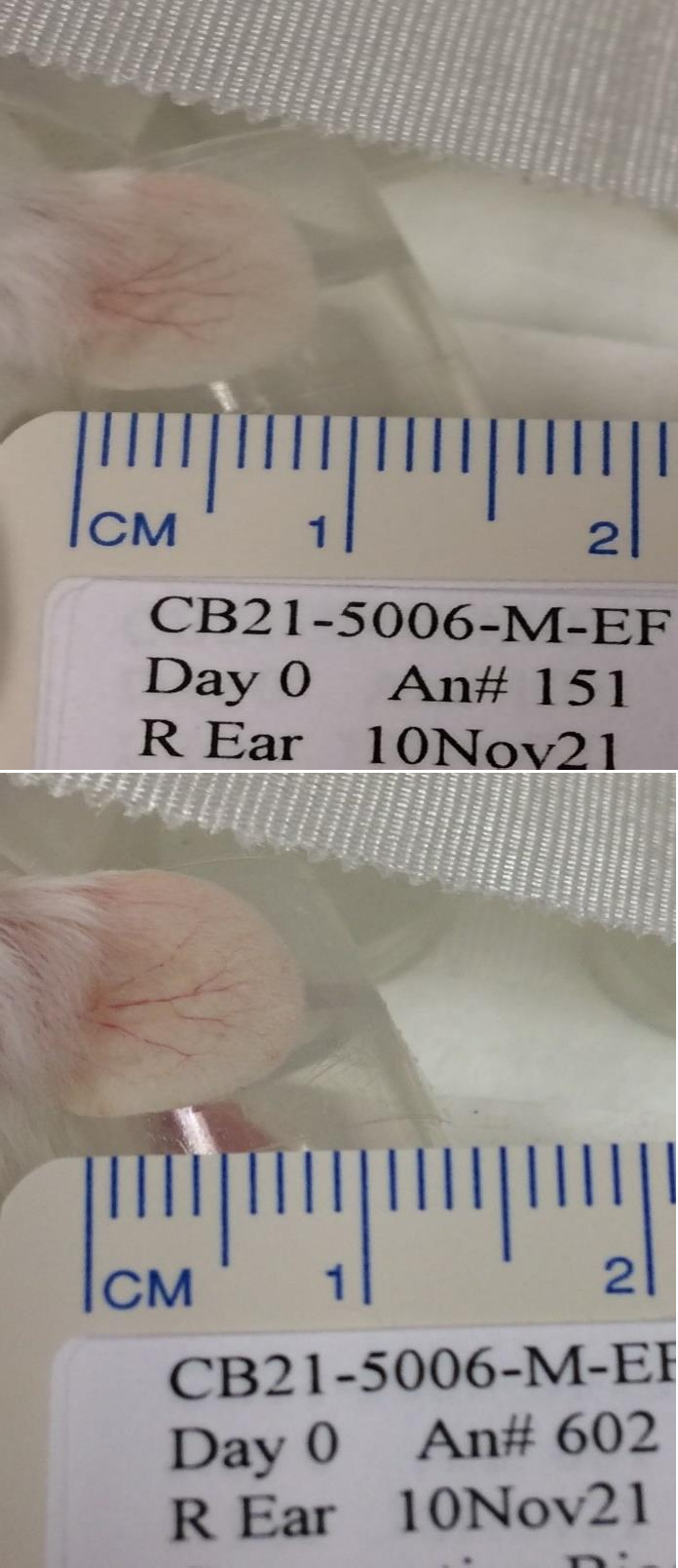

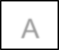

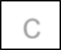

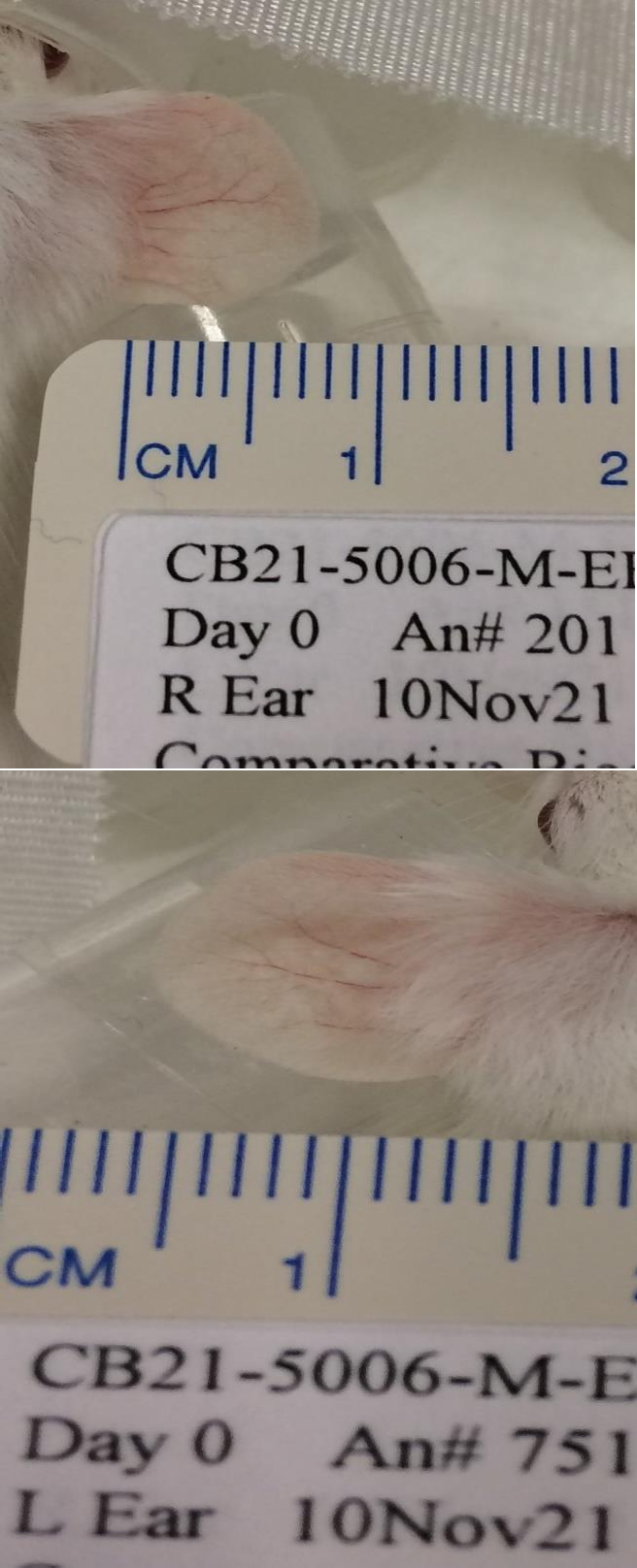

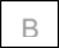

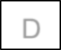


| **Day 0** | |
| --- | --- |
| A | B |
| C | D |
| **A-D: ERY=0, EDE=0** | |


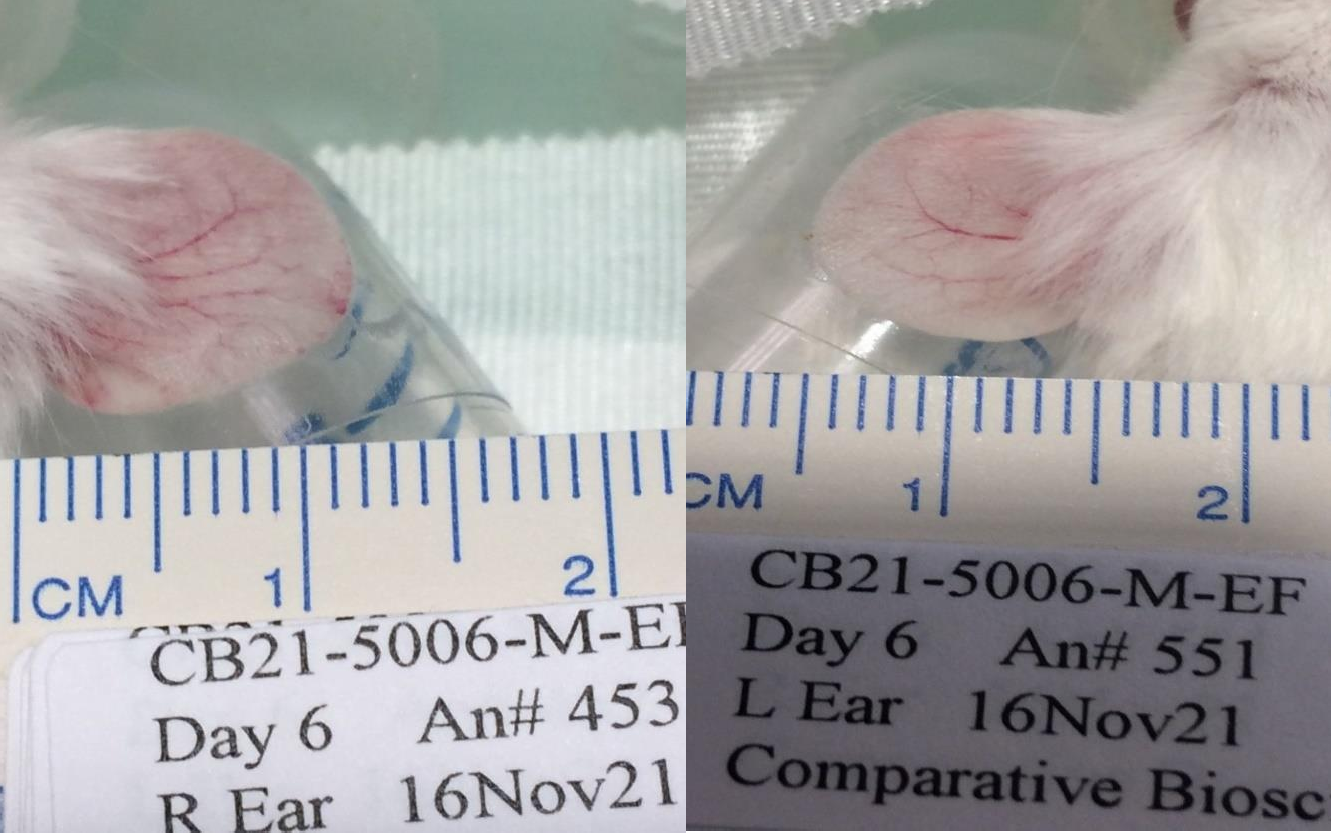

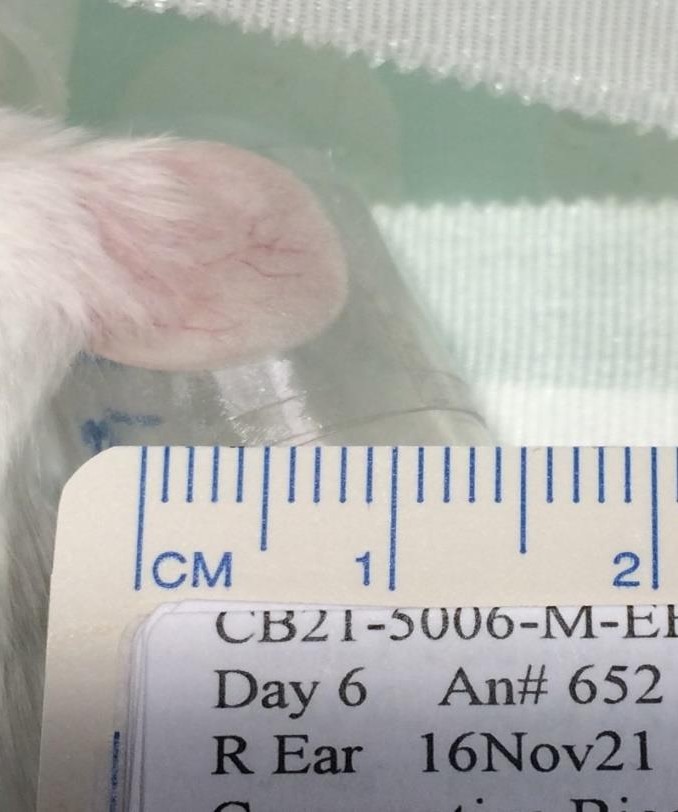

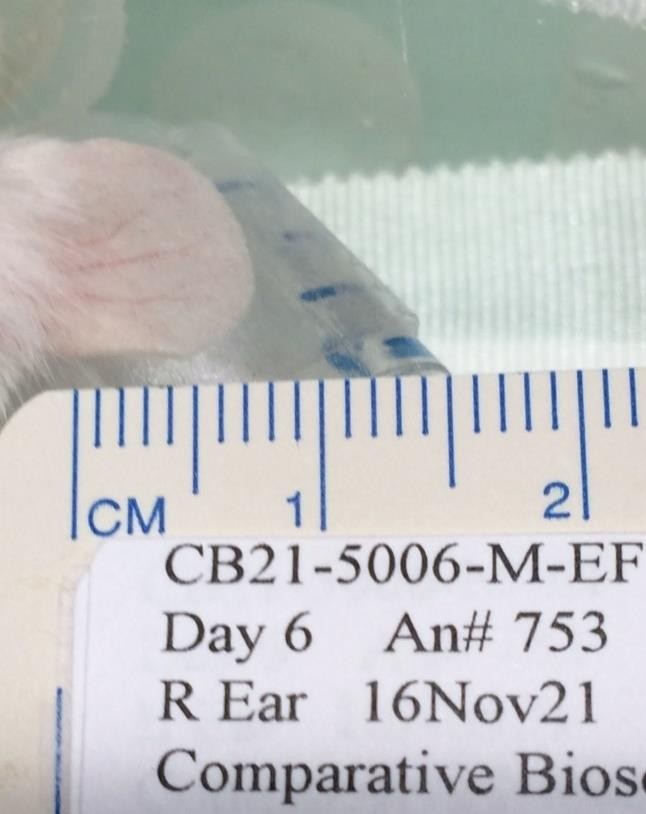

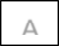

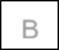

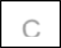

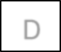


| **Day 6** | |
| --- | --- |
| A | B |
| C | D |
| **A: ERY=1, EDE=1; B: ERY=1, EDE=1; C: ERY=0, EDE=1; D: ERY=0, EDE=0** | |

### Figure 3. Representative Ear Photographs, Day 9


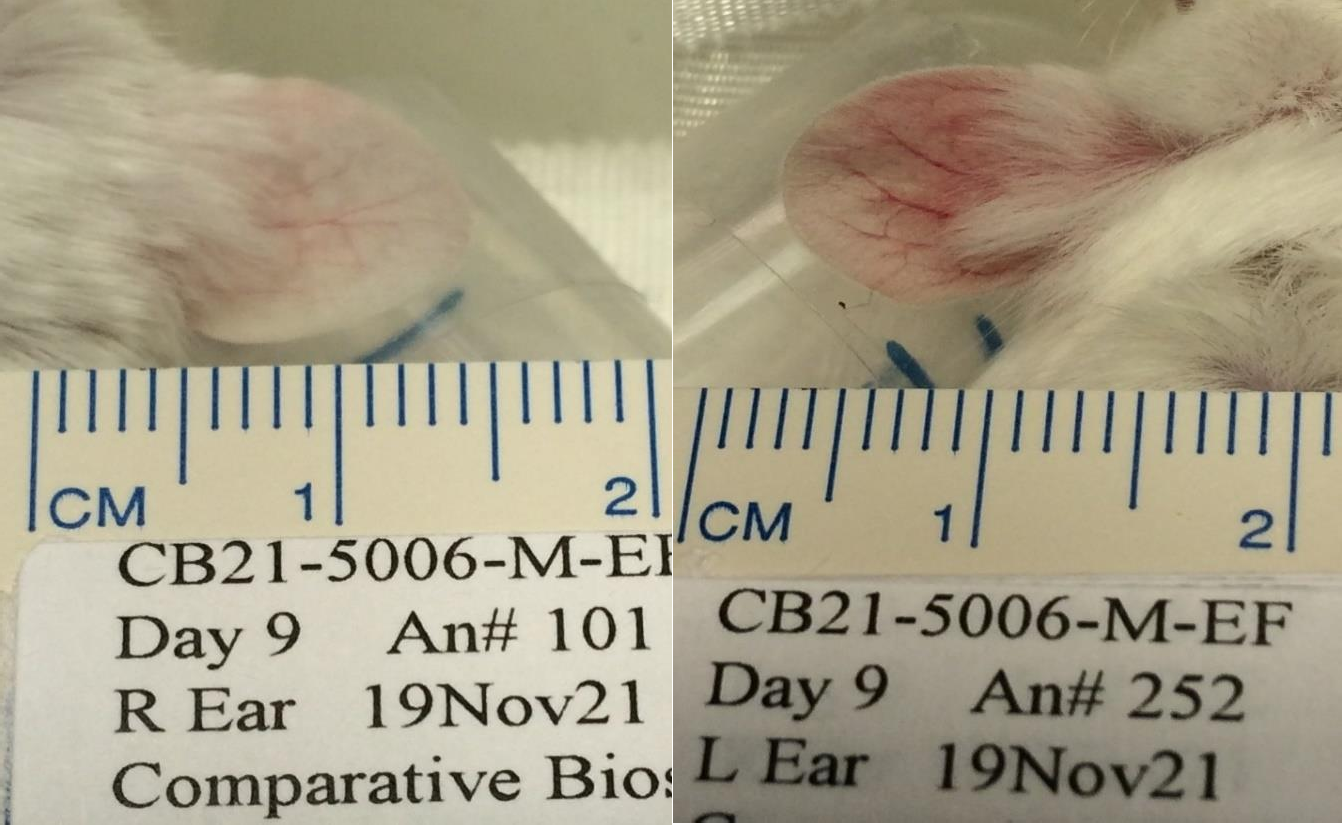

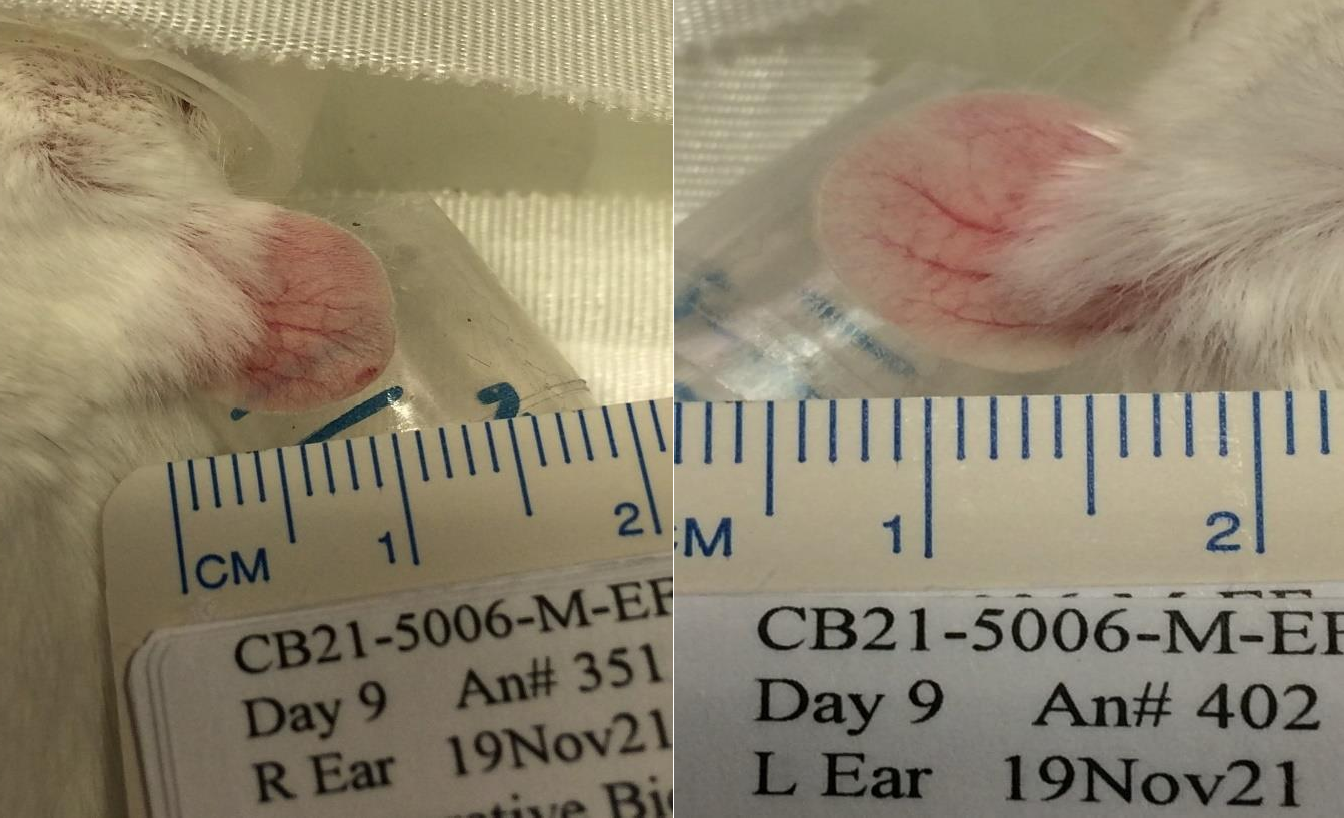

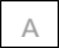

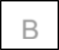

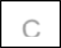

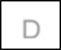


| **Day 9** | |
| --- | --- |
| A | B |
| C | D |
| **A: ERY=0, EDE=0; B: ERY=1, EDE=2; C: ERY=2, EDE=2; D: ERY=1, EDE=2** | |

### Figure 4. Representative Ear Photographs, Day 9


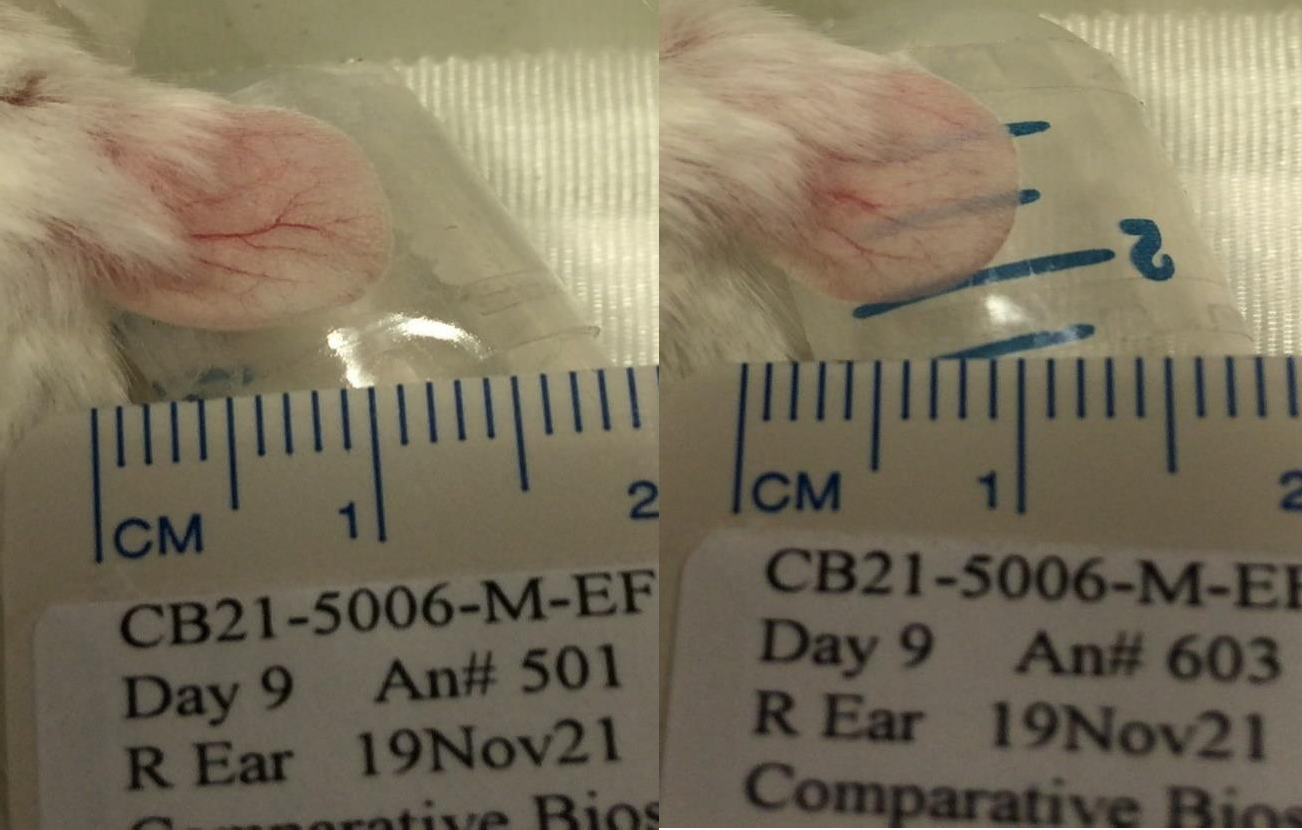

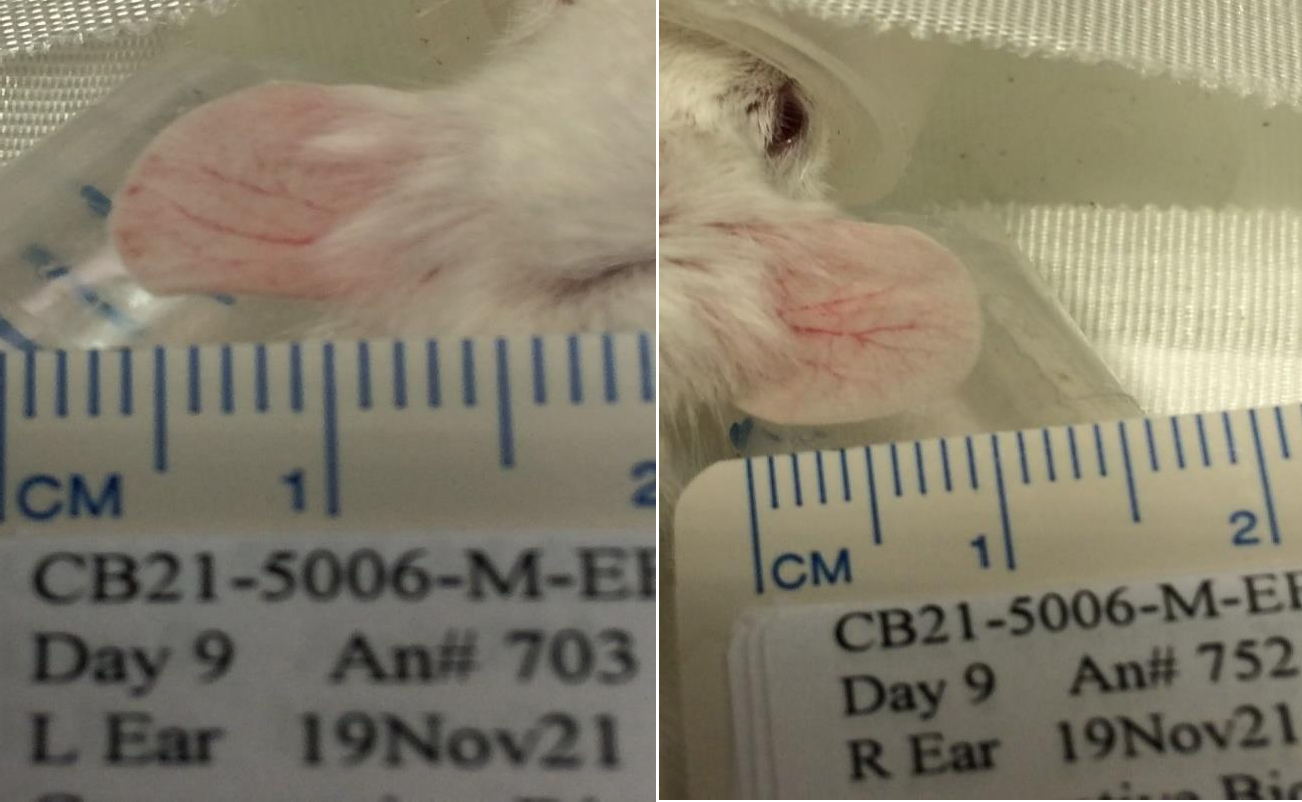

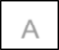

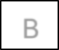

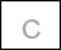

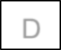


| **Day 9** | |
| --- | --- |
| A | B |
| C | D |
| **A: ERY=0, EDE=1; B: ERY=1, EDE=1; C: ERY=0, EDE=0; D: ERY=0, EDE=0** | |

ERY-erythema; EDE-edema

### Table 12. Histology Scores (Efficacy Study)

| **Group** | **Animal Number** | **Subacute Inflammation Left Ear** | **Epithelial hyperplasia Left Ear** | **Subacute Inflammation Right Ear** | **Epithelial hyperplasia Right Ear** | **Histopathologic Findings** |
| --- | --- | --- | --- | --- | --- | --- |
| **1**  **Naïve, Control** | **101** | 0 | 0 | 0 | 0 | No findings |
|  | **151** | 0 | 0 | 0 | 0 |  |
|  | **Mean** | **0** | **0** | **0** | **0** |  |
|  | **SD** | **0.0** | **0.0** | **0.0** | **0.0** |  |
| **2**  **Induced, Control** | **201** | 4 | 4 | 4 | 4 | Severe acute suppurative inflammation composed of neutrophils and fewer lymphoid cells with edema and hyperemia, consistent in all animals. There is marked hyperplasia of the epithelium with multifocal ulceration and loss of the epithelium. |
|  | **202** | 3 | 3 | 3 | 3 |  |
|  | **251** | 4 | 4 | 4 | 4 |  |
|  | **252** | 4 | 4 | 4 | 4 |  |
|  | **Mean** | **3.75** | **3.75** | **3.75** | **3.75** |  |
|  | **SD** | **0.5** | **0.5** | **0.5** | **0.5** |  |
| **3**  **Induced, Vehicle** | **301** | 3 | 3 | 3 | 3 |  |
|  | **302** | 3 | 3 | 3 | 3 |  |
|  | **351** | 4 | 4 | 3 | 3 |  |
|  | **352** | 4 | 4 | 3 | 3 |  |
|  | **Mean** | **3.5** | **3.5** | **3** | **3** |  |
|  | **SD** | **0.6** | **0.6** | **0.0** | **0.0** |  |
| **4**  **Induced, Low dose** | **401** | 3 | 3 | 3 | 3 |  |
|  | **402** | 3 | 3 | 3 | 3 |  |
|  | **403** | 3 | 3 | 3 | 3 |  |
|  | **451** | 4 | 4 | 4 | 4 |  |
|  | **452** | 4 | 4 | 4 | 4 |  |
|  | **453** | 4 | 4 | 4 | 4 |  |
|  | **Mean** | **3.5** | **3.5** | **3.5** | **3.5** |  |
|  | **SD** | **0.5** | **0.0** | **0.5** | **1.0** |  |
| **5**  **Induced, Mid dose** | **501** | 3 | 3 | 3 | 3 | Moderate suppurative inflammation composed of neutrophils and fewer lymphoid cells with edema and hyperemia, consistent in all animals. There is moderate hyperplasia of the epithelium with multifocal ulceration and loss of the epithelium. |
|  | **502** | 3 | 2 | 3 | 2 |  |
|  | **503** | 3 | 3 | 3 | 3 |  |
|  | **551** | 3 | 3 | 3 | 3 |  |
|  | **552** | 2 | 2 | 2 | 2 |  |
|  | **553** | 3 | 3 | 3 | 3 |  |
|  | **Mean** | **2.8** | **2.7** | **2.8** | **2.7** |  |
|  | **SD** | **0.4** | **0.0** | **0.4** | **1.0** |  |
| **6**  **Induced, High dose** | **601** | 2 | 2 | 2 | 2 |  |
|  | **602** | 3 | 2 | 3 | 2 |  |
|  | **603** | 2 | 3 | 2 | 3 |  |
|  | **651** | 3 | 3 | 3 | 3 |  |
|  | **652** | 3 | 3 | 3 | 3 |  |
|  | **653** | 3 | 3 | 3 | 3 |  |
|  | **Mean** | **2.7** | **2.7** | **2.7** | **2.7** |  |
|  | **SD** | **0.5** | **0.0** | **0.5** | **1.0** |  |
| **7**  **Induced, Positive Control** | **701** | 2 | 3 | 1 | 1 | Mild suppurative inflammation composed of neutrophils and fewer lymphoid cells with edema and hyperemia, consistent in all animals.  There is moderate hyperplasia of the epithelium with multifocal ulceration and loss  of the epithelium. |
|  | **702** | 2 | 3 | 2 | 3 |  |
|  | **703** | 1 | 2 | 1 | 2 |  |
|  | **751** | 1 | 3 | 1 | 3 |  |
|  | **752** | 1 | 2 | 1 | 2 |  |
|  | **753** | 1 | 3 | 1 | 3 |  |
|  | **Mean** | **1.3** | **2.7** | **1.2** | **2.3** |  |
|  | **SD** | **0.5** | **0.0** | **0.4** | **1.0** |  |


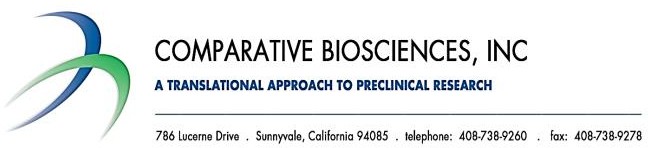


# APPENDIX A: Protocol and Amendment

This Appendix consists of 18 pages, including this cover page.

Comparative Biosciences, Inc. Study Report


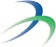

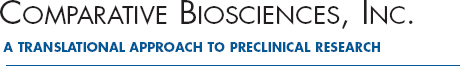


# STUDY PROTOCOL

**Model Development and Efficacy Study of an Orally Administered HSP90 Inhibitor (RGRN-305) in Atopic Dermatitis Induced by MC903 in BALB/c Mice**

# Study Number: CB21-5006-M-EF

## Testing Facility: Comparative Biosciences, Inc.

**786 Lucerne Drive**

## Sunnyvale, CA 94085

**Sponsor: Regranion**

## 300 West Coleman Boulevard, Suite 206 Mount Pleasant, SC 29464

**Sponsor’s Test Article: RGRN-305**


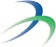

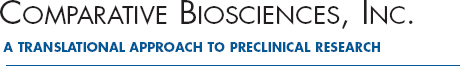


**TABLE OF CONTENTS**

### [TABLE OF CONTENTS 2](#_bookmark70)

### [APPROVALS 3](#_bookmark71)

### [TITLE 4](#_bookmark72)

### [STUDY OBJECTIVE 4](#_bookmark73)

### [SPONSOR AND SPONSOR'S REPRESENTATIVE 4](#_bookmark74)

### [TESTING FACILITY 4](#_bookmark75)

- 1. [OFFICES AND LABORATORIES 4](#_bookmark76)
  2. [STUDY TEAM 4](#_bookmark77)
  3. [TEST SITES 5](#_bookmark78)

### [REGULATORY STATUS OF PROJECT 5](#_bookmark79)

### [TEST SYSTEM 5](#_bookmark80)

### [ANIMAL CARE, HOUSING, AND ENVIRONMENTAL CONDITIONS 5](#_bookmark81)

- 1. [INSTITUTIONAL ANIMAL CARE AND USE COMMITTEE APPROVAL 5](#_bookmark82)
  2. [RECEIPT AND ACCLIMATION 6](#_bookmark83)
  3. [DROP OUTS AND REPLACEMENTS 6](#_bookmark84)
  4. [ENVIRONMENT AND HUSBANDRY 6](#_bookmark85)

### [TEST AND CONTROL ARTICLES 7](#_bookmark86)

- 1. [INDUCTION AGENT 7](#_bookmark87)
  2. [TEST ARTICLE 8](#_bookmark88)
  3. [VEHICLE ARTICLE 9](#_bookmark89)

### [EXPERIMENTAL DESIGN 9](#_bookmark90)

[TABLE 1. SUMMARY OF MODEL DEVELOPMENT PILOT STUDY DESIGN 10](#_bookmark91)

[TABLE 2. SUMMARY OF EFFICACY STUDY DESIGN 11](#_bookmark92)

### [OBSERVATIONS, MEASUREMENTS, AND SAMPLES 12](#_bookmark93)

[TABLE 3: MODIFIED DRAIZE SCORING METHOD 12](#_bookmark94)

### [DATA PRESENTATION AND STATISTICAL ANALYSIS 13](#_bookmark95)

### [RECORDS AND REPORTS 14](#_bookmark96)

### [MONITORING OF STUDY 14](#_bookmark97)

### [ADMINISTRATIVE PROCEDURES 14](#_bookmark98)

### [REFERENCES 15](#_bookmark99)

Comparative Biosciences, Inc. STUDY PROTOCOL

CB21-5006-M-EF Page 2 of 15


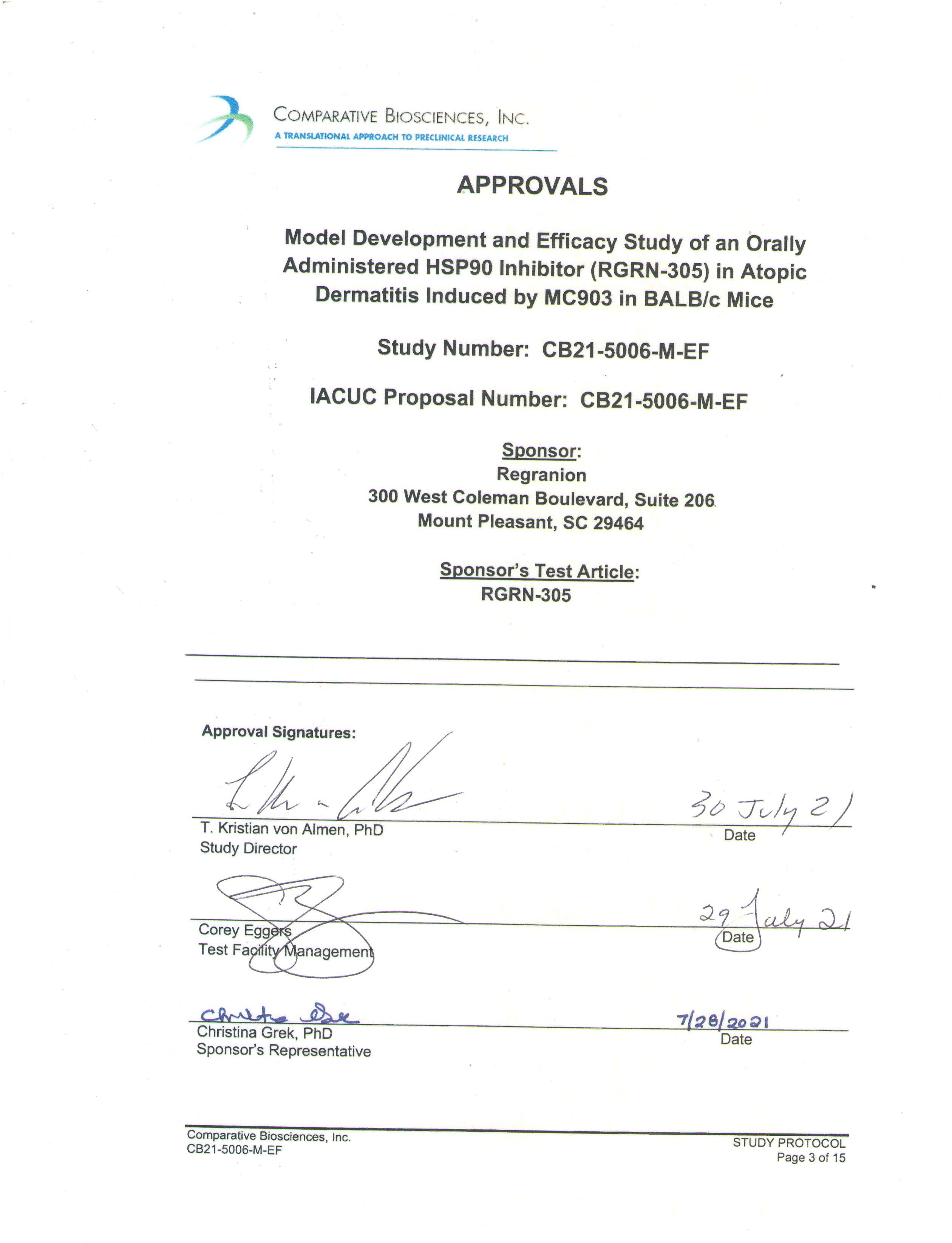


# Model Development and Efficacy Study of an Orally Administered HSP90 Inhibitor (RGRN-305) in Atopic Dermatitis Induced by MC903 in BALB/c Mice

**Study Number: CB21-5006-M-EF**

### TITLE

Model Development and Efficacy Study of an Orally Administered HSP90 Inhibitor (RGRN- 305) in Atopic Dermatitis Induced by MC903 in BALB/c Mice

### STUDY OBJECTIVE

The objective of this study is to provide data on the efficacy of an orally administered HSP90 Inhibitor (RGRN-305) in atopic dermatitis induced by MC903 in mice.

### SPONSOR AND SPONSOR'S REPRESENTATIVE

Regranion

300 West Coleman Boulevard, Suite 206 Mount Pleasant, SC 29464

Sponsor’s Representative: Christina Grek, PhD

Chief Scientific Officer Phone: 843-532-8470

Email: [grek@regranion.com](mailto:grek@regranion.com)

### TESTING FACILITY

- 1. **Offices and Laboratories**

Comparative Biosciences, Inc. 786 Lucerne Drive

Sunnyvale, CA 94085

### Study Team

Study Director: T. Kristian von Almen, PhD

Lead Biologist: Ethan Haslett

Attending Veterinarian: Miranda Abrahams, DVM

Study Pathologist: Carol Meschter, DVM, PhD, DACVP

### Test Sites

Cytokine Analysis:

The Cytokine Core, LLC 5108 West 79th Street Indianapolis, IN 46268

(317) 973-4079

ATTN: Jon Follas

[jon@multiplex-cytokine-analysis.com](mailto:jon@multiplex-cytokine-analysis.com) Tissue Analysis

Icahn School of Medicine at Mount Sinai Icahn Medical Institute #13-76

1424 Madison Ave New York, NY 10029 212-659-1617

ATTN: Yeriel Estrada [yeriel.estrada@mssm.edu](mailto:yeriel.estrada@mssm.edu)

### REGULATORY STATUS OF PROJECT

This study will be conducted in accordance with this protocol and Testing Facility Standard Operating Procedures. It will not be conducted in compliance with the US Food and Drug Administration’s Good Laboratory Practices regulations (21 CFR Part 58).

### TEST SYSTEM

**Species:** Outbred mouse (*Mus musculus*)

**Strain/Stock:** Balb/C

**Vendor:** Jackson Laboratory or other approved vendor

**Age:** Approximately 6-8 weeks

**Sex:** Males and Females

**Body weight range:** Approximately 20-25 g

**Justification for use:** Mice are an accepted species frequently used in pre-clinical evaluation of drugs and biologics intended for human use.

**Number required for study:** Total 42 mice (21 males, 21 females) on study plus 2 male and 2 female spares (Model development = 4M, 4F; Main Study = 17M, 17F)

### ANIMAL CARE, HOUSING, AND ENVIRONMENTAL CONDITIONS

- 1. **Institutional Animal Care and Use Committee Approval**

This study will be conducted according to a research proposal approved by the Institutional Animal Care and Use Committee of Comparative Biosciences, Inc.

### Receipt and Acclimation

Animals will be acclimated for a minimum of three (3) days after receipt at Comparative Biosciences, Inc. Animals will be observed daily during the acclimation period for clinical signs of abnormality. If, in the opinion of the Study Director or veterinarian the health status of any animal is questionable, that animal may be excluded from the study.

### Drop outs and Replacements

Any animal determined to be unfit for the study during the acclimation period may be excluded from the study. After initial dosing, an animal may be replaced on or after Day 0 at the discretion of the Study Director. Records of the reasons for replacement will be maintained and explained in the study report. If an animal is excluded or replaced, any data already collected on that animal will be retained.

- 1. Environment and Husbandry 7.4.1.Temperature and Humidity

Temperature controls will be set to maintain room temperature within the range of 68-79 ºF (20-26 ºC). Relative humidity in the facility is generally within the range of 30-70%. These environmental parameters will be monitored and daily minima and maxima recorded. Any excursions from these ranges will be noted in the study report.

- - 1. Light Cycle

Twelve hours of light and twelve hours of dark will be provided in the animal rooms. A fluorescent light source will be used, with lights turned on at approximately 0700 hours and turned off at approximately 1900 hours each day.

- - 1. Feed

LabDiet® 5001 Rodent Diet (Purina Mills, Inc., St. Louis, MO) or other approved diet will be provided *ad libitum* throughout the acclimation and in-life phases. Lot number(s) and Certificate(s) of Analysis (as applicable) will be maintained by the Testing Facility. There are no known contaminants that are reasonably expected to be present in the diet that are known to be capable of interfering with the purpose or conduct of the study.

- - 1. Water

Fresh water from the Sunnyvale Municipal Water Supply will be provided *ad libitum* to the animals via water bottles. The water supply is periodically tested by the City of Sunnyvale for chemical and bacterial contamination. In addition, the Testing Facility tests water from its own taps for bacteria at least once a year. Results of these analyses (City and Testing Facility) will be maintained on file at the Testing Facility. There are no known contaminants

that are reasonably expected to be present in the water that are known to be capable of interfering with the purpose or conduct of the study.

- - 1. Husbandry

Animals will be group-housed in plastic static micro-isolator cages in a room dedicated to rodents. General procedures for animal housing and husbandry will meet all regulations concerning use of animals in research including the U.S. Department of Agriculture regulations (9 CFR Ch. 1) implementing the Animal Welfare Act (7 USC 2131 *et seq*.) and the recommendations of the National Research Council’s *Guide for Care and Use of Laboratory Animals* (National Academy Press, 2011).

- - 1. Animal Identification

Mice will be arbitrarily assigned sequential temporary animal identification numbers after receipt at the Testing Facility. The study number and temporary identification number will be displayed on each cage card during the acclimation period. Upon allocation to a study group, animals will be assigned “permanent” identification numbers as described in the Final Selection and Assignment of Animals, below. Permanent identification numbers will be displayed on cage cards and coded on individual mice by tail marks.

### TEST AND CONTROL ARTICLES Safety Data Sheets (SDS)

The Sponsor will provide a Safety Data Sheet (SDS) (formerly MSDS or Material Safety Data Sheets) or equivalent for each Test or Control Article supplied to the Testing Facility by the Sponsor.

### Induction Agent.

Atopic dermatitis induction agent will be MC903. MC903 is formulated to contain calcipotriol as an active ingredient. The pre-formulated solution will be provided by the Testing Facility.

- - 1. **Source:** Test Facility will obtain MC903 (Sigma, St. Louis, Missouri or other approved vendor) in amount sufficient for study.
    2. **Lot Number:** The lot number will be recorded and included in the study report.
    3. **Storage:** MC903 will be stored according to manufacturer’s instructions.
    4. **Expiration Date:** The expiration date, if any, will be recorded and included in the study report.
    5. **Dose Preparation:** No dose preparation needed as the Induction Agent, MC903, will be provided in ready-to-use form.
    6. **Special Handling:** Handling per manufacturer instruction. Standard laboratory precautions.

### Test Article.

Test Article, REGRN-305 (an HSP90 Inhibitor), will be provided by Sponsor as a lyophilized powder.

- - 1. **Source:** Sponsor
    2. **Lot Numbers:** The lot or batch number will be recorded and included in the study report.
    3. **Storage:** The Test Article will be stored at room temperature (20-26 ºC).
    4. **Expiration Date:** The expiration dates, if available, will be recorded and included in the study report.

**Dose Preparation:** Test Article will be supplied as a lyophilized powder. Test Article: RGRN-305 will be supplied as a lyophilized powder by Sponsor.

Low (20 mg/kg), medium (40 mg/kg) and high dose (80 mg/kg) concentrations will be prepared (batches of 15 mL, stored at 4ºC and used within one week) as follows:

- - - - 120 mg of RGRN-305 will be mixed with 11 mL of vehicle solution in a glass vial
      - The solution will be mixed by vortexing for 1 minute
      - The pH will be adjusted to 4.5 with 0.1N HCl (approximately 500 uL), the solution will be vortexed regularly during pH adjustment to solubilize particles remaining on the vial wall
      - The solution will be vortexed for 10 min, this will be repeated as needed if particles remain visible
      - The solution will be completed with vehicle solution up to 15 mL
      - The solution will be filtered on 0.2 or 0.22 um GHP or PSE filters and stored in a sterile vial
    1. **Dose Analysis:** Dose analysis is not planned.
    2. **Characterization:** The Sponsor is responsible for characterization and stability of the Test Article and will provide a C of A, or equivalent documentation, for inclusion in the final report. The raw data generated by the Sponsor in support of this C of A or its equivalent will not be verified or maintained by Comparative Biosciences.
    3. **Special Handling:** Standard laboratory precaution. At end of the study, remaining Test Articles will be returned to Sponsor or disposed of with biological waste.
    4. **Archiving Samples:** Not planned for this study.

### Vehicle Article:

Vehicle Article is 5% Kleptose solution (pH 4.5). The Testing Facility will obtain the Vehicle Article from a commercial source and provide an amount sufficient for use in the study.

- - 1. **Source:** Commercial vendor.
    2. **Lot Number:** The lot number will be recorded in the raw data.
    3. **Storage:** Vehicle solution will be stored at 4ºC.
    4. **Expiration Date:** The expiration date will be recorded in the raw data.
    5. **Dose Preparation:** Vehicle solution will be prepared in batches of 30 mL (stored at 4ºC and used within one week) as follows:
       - 1.5 g of Kleptose (HPB Oral grade) will be dissolved in 20 mL deionized water in a glass vial
       - The solution will be mixed by vortexing for 1 minute
       - The pH will be adjusted to 4.5 with 0.1N HCl
       - The solution will be completed with deionized water up to 30 mL
       - The solution will be filtered on 0.2 or 0.22 um GHP or PSE filters and stored in a sterile vial
    6. **Dose Analysis:** None required.
    7. **Characterization**: Purity and identity of the Vehicle Article will be accepted based on the product labeling.
    8. **Special Handling:** None required.

### EXPERIMENTAL DESIGN

The study design is outlined in [Table 1](#_bookmark91) (Pilot Model Development Study) and [Table 2](#_bookmark92) (Efficacy Study), below.

Model development study will consist of two groups of 4 animals each (2 male, 2 female). Atopic Dermatitis (AD) will be induced bilaterally in shaved ears of all mice ([Table 1,](#_bookmark91) [ref 1](#_bookmark99)) with topical application of MC903 (1.125nmol per ear or 25 µl total: 12.5 µl on dorsal, 12.5 µl ventral side of each ear). Group 1 will receive no treatment; Group 2 will be treated orally with Positive Control (Dexamethasone). Daily Draize scores, ear thickness measures and photos will be taken for nine days, beginning Day 0 (prior to induction) on weekdays only. This portion of study may be extended to assure observable skin changes in ears compatible with AD.

Efficacy study will consist of the following. Group 1 will be naïve, untreated controls. AD will be induced in Groups 2-7 with topical administration of MC9031 (1.125nmol per ear or 25 µl total: 12.5 µl on dorsal, 12.5 µl ventral side of each ear) applied to both shaved ears from Day

0-4 and Day 7-8. Mice in Groups 2-7 will be administered no treatment, oral Vehicle, oral Test Article (low, medium or high dose), or oral Positive Control (Dexamethasone) prior to induction of skin inflammation, then daily thereafter through in-life. Clinical observations, body weights, Draize scores and ear thickness will be measured and photos taken. Terminal blood will be collected and sent to Cytokine Core, LLC (Indianapolis, IN) for cytokine analysis. Necropsy will be performed at end term and ear tissue will be collected and standardly processed for histopathology. Slides will be read by a board-certified veterinary pathologist. Additional tissue samples (fixed, snap frozen, and stored in RNAlater) will be collected and shipped to the Icahn School of Medicine at Mount Sinai (New York, NY). Carcasses will be disposed of with no further analysis.

### Table 1. Summary of Pilot Model Development Study Design

| **Group** | **Animal Numbers** | **Induction^1^ (topical)** | **Treatment^2^** | **In-Life Duration** | **Tests or Assays** |
| --- | --- | --- | --- | --- | --- |
|  | **101-102M** | MC903 |  |  |  |
| **1** |  | 25 µl per ear (12.5 µl dorsal,  12.5 µl ventral) (1.125nmol per ear)  Applied to both shaved ears (Day 0 – Day 4;  Day 7-84) | NA | Acclimation  +  9 Days4 | - Daily clinical observations - Weekly body weights - Daily Draize scoring3 - Ear thickness3, with photos |
|  | **151-152F** |  |  |  |  |
| **2** | **203-204M** |  | Positive Control (Dexamethasone) oral |  |  |
|  | **253-254F** |  |  |  |  |

M=male, F=female, 1 Administered 1 hour after Test/Control Article, 2 Daily administration Day 0-Day 8,

3 Measurements prior to TA administration-weekdays only, 4=option to extend study per Sponsor request

### Table 2. Summary of Efficacy Study Design

| **Group** | **Animal Numbers** | **Induction^1^ (topical)** | **Treatment^2^** | **Dose (mg/kg)** | **In-Life Duration** | **Tests or Assays** |
| --- | --- | --- | --- | --- | --- | --- |
| **1** | 101M  151F | N/A | Naïve, untreated | N/A |  | - Daily clinical observations |
| **2** | 201-202M  251-252F | MC903  Topical Administration 25 µl per ear  (12.5 µl dorsal,  12.5 µl ventral) (1.125nmol per ear)  Applied to both shaved ears Day 0 – Day 4;  Day 7-84) | Model Control (no treatment) | N/A |  | - Weekly body weights |
| **3** | 301-302M |  | Vehicle | N/A |  | - Daily Draize scoring, EOD |
|  | 351-352F |  | oral |  |  | ear thickness3, with photos |
| **4** |  |  |  | 20 |  |  |
|  | 401-403M |  | RGRN-305 |  |  | (weekdays only) |
|  | 451-453F |  | oral |  | Acclimation  +  9 Days4 | - Terminal blood collection (for cytokine analysis) |
| **5** | 501-503M  551-553F |  | RGRN-305  oral | 50 |  |  |
| **6** | 601-603M |  | RGRN-305 | 100 |  |  |
|  | 651-653F |  | oral |  |  | - Necropsy, collect ears |
| **7** | 401-403M  451-453F |  | Positive Control (Dexamethasone) oral | 5 |  | - Histopathology of ears - Remaining ear tissue:   1) fixed, 2) snap frozen, |
|  |  |  |  |  |  | 3) stored in RNAlater, |

M=male, F=female, EOD=every other day, 1 Administered 1 hour after Test/Control Article, 2 Daily administration Day 0-Day 8,

3 Measurements prior to TA administration, 4=option to extend study per Sponsor request

- 1. Dosing Cohorts

Animals will be dosed as a single cohort.

- 1. Rationale for Selection of Dose and Route

The dose and route of administration were selected by the Sponsor based on the anticipated clinical use.

- 1. Final Selection and Assignment of Animals

No earlier than one (1) day prior to dosing, all animals will be examined for general health by a qualified veterinarian. Animals not excluded for health reasons will be weighed and selected for the study based on normal clinical presentation and moderate body weight. Animals will be randomly assigned study groups. Details of the allocation procedure will be filed with the raw data.

- 1. Animal Identification

Upon assignment to a study group, each animal will be uniquely identified with ear punches and/or ear marks and/or tail marks. A cage card displaying the animals’ permanent identification numbers and the study number will be displayed throughout the in-life period. The animal identification numbers and their groups are shown in [Table 1.](#_bookmark91)

- 1. Dose Administration

On the day of dosing, Test Article, RGRN-305 8 mg/mL, will be diluted in Vehicle to permit dosing at low (20 mg/kg), medium (50 mg/kg) and high dose (100 mg/kg). Dose volumes will be calculated based on body weights. Animals will be dosed orally via gavage.

- 1. Adverse Reactions

Animals experiencing adverse reactions may receive supportive veterinary care if, in the opinion of the Study Director and the veterinarian, such treatment will not interfere with the purpose or conduct of the study. Moribund animals will be euthanized after veterinary consultation. Animals in extreme pain or distress may be euthanized by qualified personnel without veterinary consultation.

- 1. Unscheduled/Early Death

If an animal dies on study or is judged to be moribund and euthanized, it will be submitted for gross necropsy. An attempt will be made to determine if the death or moribund condition was Test Article-related. Tissues will be fixed for histopathologic examination at the discretion of Study Pathologist and Sponsor. The Sponsor will be notified promptly.

### OBSERVATIONS, MEASUREMENTS, AND SAMPLES

- 1. Clinical Observations

Clinical observations, including overt signs of non-normal response will be recorded once daily in all animals from Day 0 and throughout the study duration. All signs of clinical abnormality will be recorded.

- 1. Body Weights

The animals will be weighed prior to Day 0, then weekly during study conduct.

- 1. Draize Scoring

Draize scores will be recorded daily (weekdays only) for edema and erythema according to [Table 3.](#_bookmark94) A representative photograph will be taken at the time of scoring. Additional photographs may be taken at the Sponsor’s request as an additional Work Order.

| **Table 3: Modified Draize Scoring Method** | |
| --- | --- |
| **Erythema** | **Edema** |
| Grade 0: No erythema | Grade 0: No edema |
| Grade 1: Slight erythema | Grade 1: Slight edema |
| Grade 2: Erythema clearly visible | Grade 2: Moderate edema |
| Grade 3: Moderate erythema | Grade 3: Intense edema with bulging |
| Grade 4: Intense erythema | - |

- 1. Ear Thickness

Ear thickness will be measured using a caliper device. Measures will be taken every other day (weekdays only).

- 1. Blood Collection

Terminal blood will be collected and samples will be shipped to Cytokine Core, LLC (Indianapolis, IN) for cytokine analysis. A mouse cytokine/chemokine panel (full panel, 32- plex) or similar will be used to analyze samples.

The Cytokine Core, LLC 5108 West 79th Street Indianapolis, IN 46268

(317) 973-4079

ATTN: Jon Follas

[jon@multiplex-cytokine-analysis.com](mailto:jon@multiplex-cytokine-analysis.com)

- 1. Necropsy

The animals will be euthanized on Day 9 (unless the study is extended per Sponsor request). Necropsy will be performed at end term and ears will be collected. Punch biopsies (3 mm, taken from the center of the injection area) will be standardly processed for histology evaluation. Remaining ear tissue will be separated as follows for additional analysis. Fixed tissue, snap frozen samples and samples stored in RNAlater will be collected. All samples will be shipped to Icahn School of Medicine, Mount Sinai (New York, NY) for analysis (see attached manual for processing and shipping details). Carcasses will be fixed in 10% neutral buffered formalin for subsequent analysis by Additional Work Order.

Icahn School of Medicine at Mount Sinai Icahn Medical Institute #13-76

1424 Madison Ave New York, NY 10029 Yeriel Estrada

[yeriel.estrada@mssm.edu](mailto:yeriel.estrada@mssm.edu)

- 1. Histopathology

Bilateral ear tissues (punch biopsies) will be examined histopathologically. Tissues will be dehydrated, embedded in paraffin, sectioned at 3-5 μm, and stained with hematoxylin and eosin. Slides will be evaluated via light microscopy by a board-certified veterinary pathologist.

### DATA PRESENTATION AND STATISTICAL ANALYSIS

- 1. Data Presentation

Data will be presented as raw data and in summary tables and/or displays as appropriate.

- 1. Statistical Analysis

11.2.1. Descriptive statistics:

Means, standard deviations, standard error of measurements, graphic displays, and other appropriate techniques will be employed as deemed necessary.

### RECORDS AND REPORTS

- 1. Study Records

The following records will be retained at the Testing Facility in accordance with 21 CFR Part 58.195: Final study protocol, protocol amendments, and study report; associated documentation and formal correspondence with Sponsor; Test/Control Article records; all study raw data; as well as pretest, in-life, and post-mortem animal records.

- 1. Final Report

The draft report will be generated and issued following data collection. Extensive comments may require additional drafts. A final report will be issued after the Sponsor’s comments on the draft report are received by the Study Director. The final report will include the final study protocol (with amendments), study raw data, interpretative results, appropriate graphs, tables and statistics, Test Article characterization information and sub-reports from Principal Investigators. In the event that review of report draft is delayed by the Sponsor greater than

90 days, then the Study Director may move forward to study finalization. Subsequent changes to the final report could be incorporated through a report amendment that would entail an additional scope of work and additional cost.

### MONITORING OF STUDY

This study will be conducted in compliance with this protocol, and with Testing Facility Standard Operating Procedures. The Sponsor may monitor the study during normal business hours by appointment with the Study Director.

### ADMINISTRATIVE PROCEDURES

- 1. Amendments to the Protocol

Any modification to the approved protocol will be documented and agreed to as an amendment. Such modifications will be approved jointly by the Sponsor and Study Director prior to the described actions being conducted. Reasons for any amendment(s) will also be documented.

The study protocol will be the controlling document in case of discrepancies between the protocol and the SOPs of Comparative Biosciences, Inc.

- 1. Deviations from the Protocol

All protocol deviations will be documented and brought to the attention of the Study Director. Any deviation judged reasonably by the Study Director to have possible impact on the outcome of the study or interpretation of the data will be communicated to the Sponsor as soon as possible and corrective actions will be determined. All protocol deviations will be summarized in the final report.

- 1. Test/Control Article, Tissues, Slides, Blocks and Sample Accountability

Comparative Biosciences, Inc. will maintain complete records of all materials provided by Sponsor including receipt, use, and disposition of materials. Comparative Biosciences Inc. will dispose of all materials one month after a final study report is issued to Sponsor or will return materials to the Sponsor pending their written instructions.

- 1. Independent Audits

The Sponsor may arrange for an independent audit of the Testing Facility and/or any of its subcontractors. Any such audit shall be conducted by appointment during regular business hours and the Sponsor shall ensure that the independent auditor(s) comply with Testing Facility procedures and policies.

- 1. Safety Data Sheets (SDS)

The Sponsor will provide a SDS or equivalent for each Test Article, Control Article, Vehicle, and/or diluent supplied to the Testing Facility by the Sponsor.

- 1. Confidentiality

All information obtained during conduct of the study will be considered confidential. At no time will the nature or the study, study protocol, or study data be disclosed by Comparative Biosciences, Inc., to any third party without written consent from the Sponsor, except as may be required by regulatory authorities. Likewise, Sponsor will treat all correspondence from Comparative Biosciences, Inc., except the final report, but including draft reports, as confidential information.

### REFERENCES

1Moosbrugger-Martinz V, Schmuth M, Dubrac S. A Mouse Model for Atopic Dermatitis Using Topical Application of Vitamin D3 or of Its Analog MC903. Methods Mol Biol. 2017;1559:91- 106. doi: 10.1007/978-1-4939-6786-5_8. PMID: 28063040.


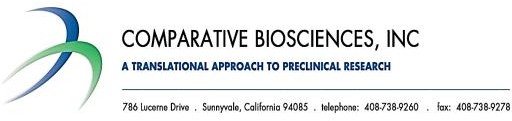


# Protocol Amendment #1

| **Study No.: CB21-5006-M-EF** | **Sponsor:** Regranion |
| --- | --- |
| **Title:** Model Development and Efficacy Study of an Orally Administered HSP90 Inhibitor (RGRN-305) in Atopic Dermatitis Induced by MC903 in BALB/c Mice | |
| **Effective:** Date of Study Director Signature | |

**Item No. 1**

Page 10, Protocol Section 9, Experimental Design

**Change:** “Mice in Groups 2-7 will be administered no treatment, oral Vehicle, oral Test Article (low, medium or high dose), or oral Positive Control (Dexamethasone) prior to induction of skin inflammation, then daily thereafter through in-life.“

**To read:** “Mice in Groups 2-7 will be administered no treatment, oral Vehicle, oral Test Article (low, medium or high dose), or subcutaneous Positive Control (Dexamethasone) prior to induction of skin inflammation, then daily thereafter through in-life.“

**Justification:** “Positive Control Article (dexamethasone) was changed to subcutaneous dosing to ensure more robust effect.”

**Item No. 2**

Page 11, Protocol Section 9, Table 2. Summary of Efficacy Study Design

**Change:**

| **Group** | **Animal Numbers** | **Induction^1^ (topical)** | **Treatment^2^** | **Dose (mg/kg)** | **In-Life Duration** | **Tests or Assays** |
| --- | --- | --- | --- | --- | --- | --- |
| **1** | 101M  151F | N/A | Naïve, untreated | N/A |  | - Daily clinical observations |
| **2** | 201-202M  251-252F | MC903  Topical Administration 25 µl per ear  (12.5 µl dorsal,  12.5 µl ventral) (1.125nmol per ear)  Applied to both shaved ears Day 0 – Day 4;  Day 7-84) | Model Control (no treatment) | N/A |  | - Weekly body weights |
| **3** | 301-302M |  | Vehicle | N/A |  | - Daily Draize scoring, EOD |
|  | 351-352F |  | oral |  |  | ear thickness3, with photos |
| **4** |  |  |  | 20 |  |  |
|  | 401-403M |  | RGRN-305 |  |  | (weekdays only) |
|  | 451-453F |  | oral |  | Acclimation  +  9 Days4 | - Terminal blood collection (for cytokine analysis) |
| **5** | 501-503M  551-553F |  | RGRN-305  oral | 50 |  |  |
| **6** | 601-603M |  | RGRN-305 | 100 |  |  |
|  | 651-653F |  | oral |  |  | - Necropsy, collect ears |
| **7** | 401-403M  451-453F |  | Positive Control (Dexamethasone) oral | 5 |  | - Histopathology of ears - Remaining ear tissue:   1) fixed, 2) snap frozen, |
|  |  |  |  |  |  | 3) stored in RNAlater, |


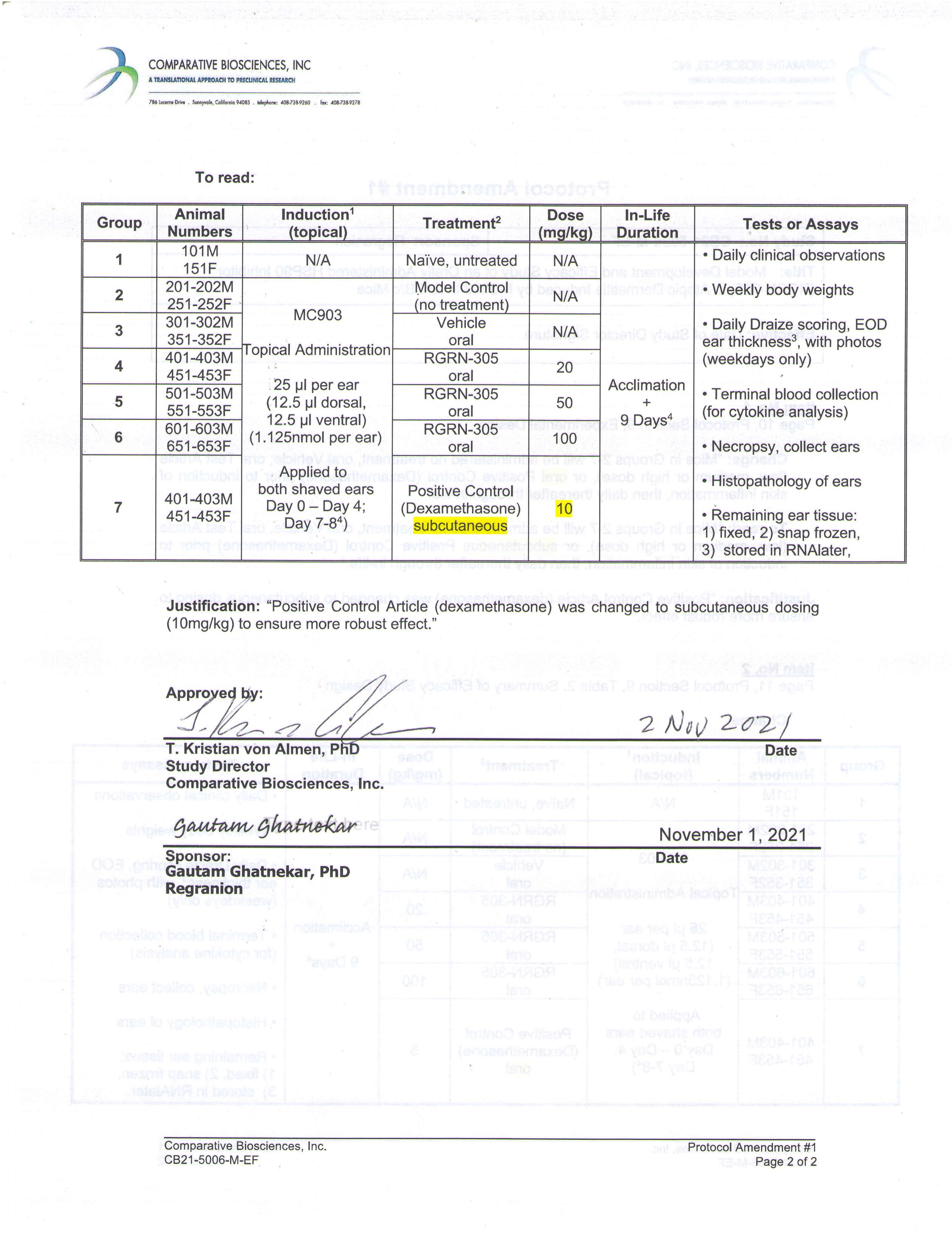


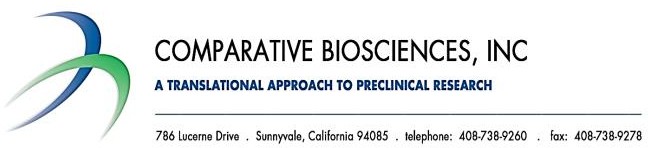


# APPENDIX B: Certificates of Analysis and MSDS

This Appendix consists of 6 pages, including this cover page.

Comparative Biosciences, Inc. Study Report

CB21-5006-M-EF Page B1 of 6


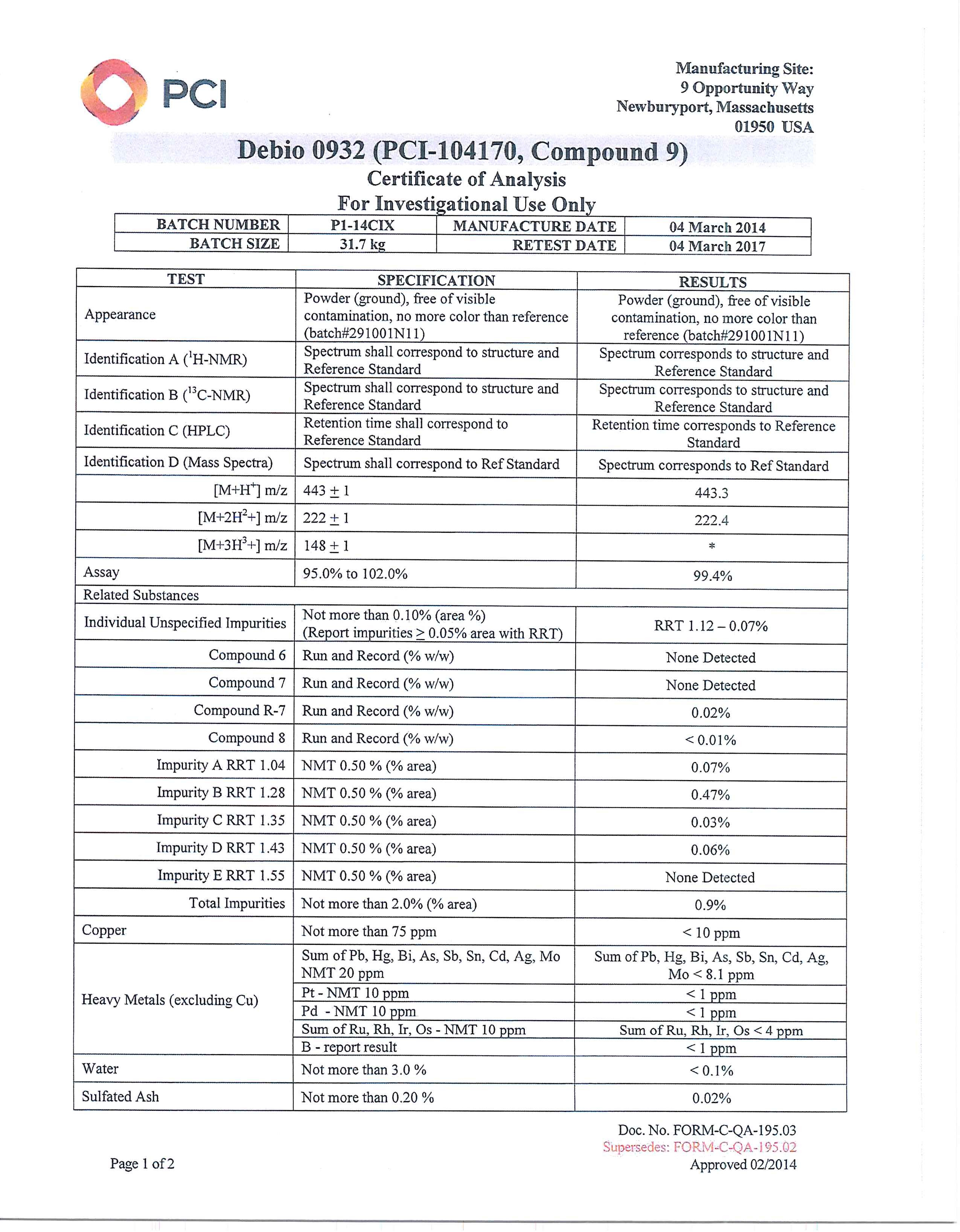


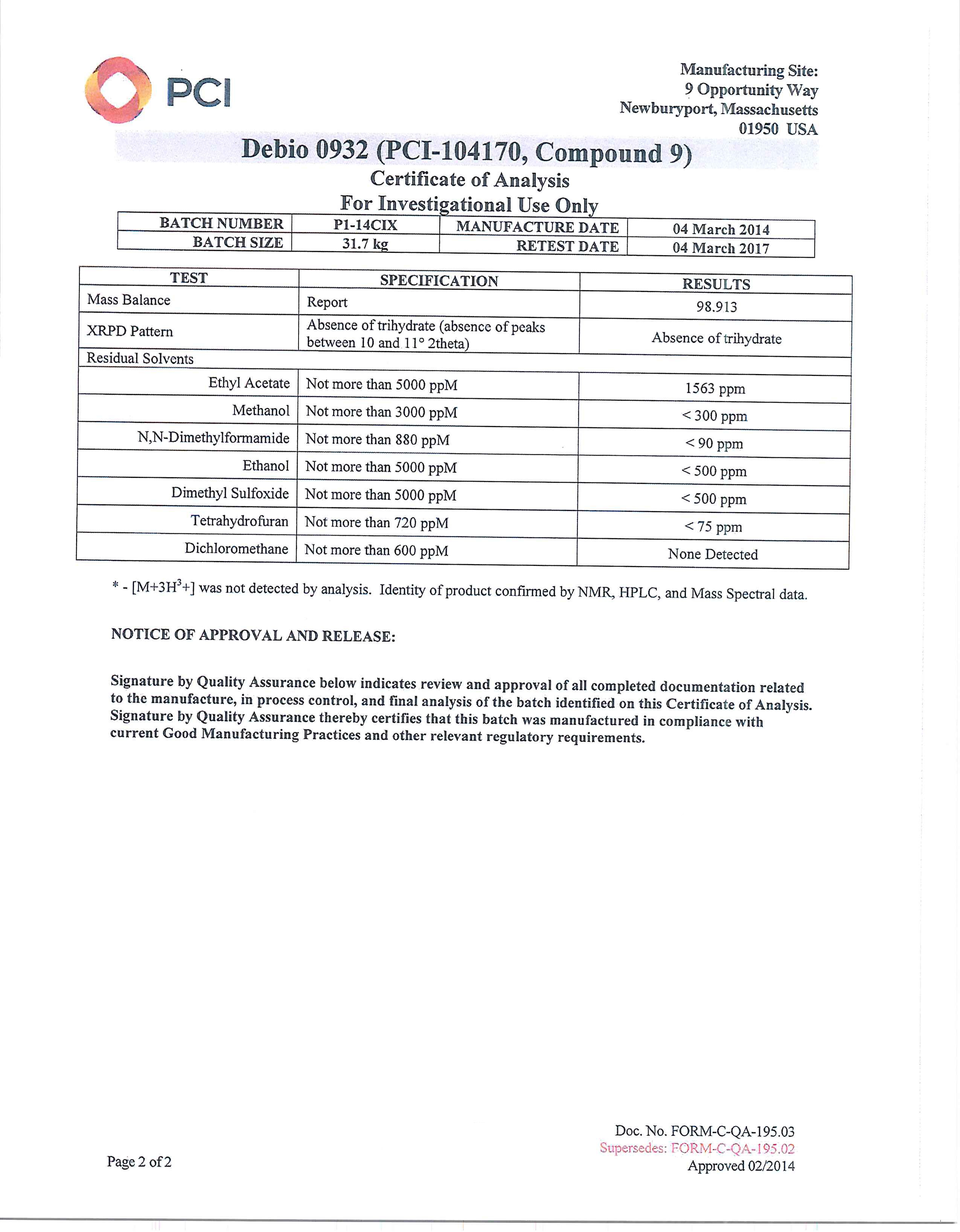


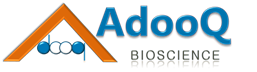

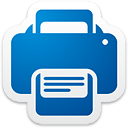
Material Safety Data Sheet

Section 1 - Chemical Product and Company Identification

**Product Name:** CUDC-305 (DEBIO-0932 )

**Catalog Numbers:** A12648 **Manufacturer** Adooq Bioscience **Address:** Adooq Bioscience

4000 Barranca Parkway, Suite 250

Irvine, CA 92604

**For information, call:** 866-930-6790 (Toll Free)

Section 2 - Composition, Information on Components

| **CAS Number:** | 1061318-81-7 |
| --- | --- |
| **Molecular Formula:** | C22H30N6O2S |
| **Molecular Weight:** | 442.58 |
| **Synonyms:** | Debio0932; CUDC305 |

Section 3 - Hazards Identification

Toxic. Contains a pharmaceutically active ingredient. Handling should only be performed by personnel trained and familiar with handling of potent active pharmaceutical ingredients. Moderate to severe irritant to the skin and eyes.

Section 4 - First Aid Measures

**Eyes:** Immediately flush eyes with plenty of water for at least 15 minutes, occasionally lifting the upper and lower eyelids. Get medical aid immediately. **Skin:** Immediately flush skin with plenty of soap and water for at least 15 minutes while removing contaminated clothing and shoes. Get medical aid if irritation develops or persists. Wash clothing before reuse.

**Ingestion:** If victim is conscious and alert, give 2-4 cupfuls of milk or water. Never give anything by mouth to an unconscious person. Get medical aid immediately. Induce vomiting by giving one teaspoon of Syrup of Ipecac.

**Inhalation:** Get medical aid immediately. Remove from exposure to fresh air immediately. If breathing is difficult, give oxygen. Do NOT use mouth-to- mouth resuscitation. If breathing has ceased apply artificial respiration using oxygen and a suitable mechanical device such as a bag and a mask.

**Notes to Physician:** Effects may be delayed. Ethanol may inhibit methanol metabolism.

Section 5 - Fire Fighting Measures

**Suitable extinguishing agents: Protective equipment:** wear self-contained breathing apparatus and protective clothing to prevent contact with skin and eyes.

**Unusual fire hazard:**emits toxic fumes such as carbon monoxide, etc.

Section 6 - Accidental Release Measures

**After Inhalation:** cordon off area of spill; wear self-contained breathing apparatus, protective clothing and heavy rubber gloves

**Measures for cleaning/collecting** absorb solutions with finely- powdered liquid-binding material (diatomite, universal binders); decontaminate surfaces and equipment by scrubbing with alcohol; dispose of contaminated material according to Section 13

Section 7 - Handling and Storage

**Handling:** void inhalation and contact with skin, eyes and clothing; material may be an irritant

**Storage:** Keep away from heat, sparks, and flame. Keep away from sources of ignition. Store in a cool, dry, well-ventilated area away from incompatible substances. Flammables-area. Keep containers tightly closed.

Section 8 - Exposure Controls, Personal Protection

**Eyes:** Wear chemical goggles.

**Skin:** Wear appropriate protective gloves to prevent skin exposure.

**Clothing:** Wear appropriate protective clothing to prevent skin exposure.

**Respirators:** A respiratory protection program that meets OSHA's 29 CFR §1910.134 and ANSI Z88.2 requirements or European Standard EN 149 must be followed whenever workplace conditions warrant a respirator's use.

Section 9 - Stability and reactivity

**Stability:** stable if stored as directed; avoid strong oxidizing agents

**Thermal decomposition / conditions to be avoided:** protect from light and heat

**Dangerous products of decomposition:** thermal ecomposition may produce toxic gases such as carbon monoxide, carbon dioxide, and nitrogen oxides

Section 10 - Toxicological information

**RTECS#:**

**Acute toxicity:** not known **Primary irritant effect:** not known **On the skin:** not known

**On the eye:** not known

Section 11 - Ecological information

**General notes:** no data available

Section 12 - Disposal consideration

Dispose of in accordance with prevailing country, federal, state and local regulations

Section 13 - Transport information

**DOT:**

**Proper shipping name:** none

**Non-Hazardous for transport:** this substance is considered to be non-hazardous for transport

**IATA class:**

**IATA class:** none

**Non-Hazardous for transport:** this substance is considered to be non-hazardous for transport

Section 14 - Regulations

**Code letter and hazard designation of product:**

**Hazard-determining components of labeling:**

**EU Risk And Safety phrases:**

S24/25: avoid contact with skin and eyes

S26: in case of contact with eyes, rinse immediately with plenty of water and seek medical advice S28: after contact with skin, wash immediately with plenty of water

S36/37/39: wear protective clothing, gloves and eye/face protection R28: very toxic if swallowed

R38: irritating to skin

R41: risk of serious damages to eyes

R48: toxic; danger of serious damage to health by prolonged exposure

**R62: possible risk of impaired fertility R63: possible risk of harm to unborn child**

Section 15 - Additional Information

**Revision #2 Date:** 1/14/2014

*The information above is believed to be accurate and represents the best information currently available to us. However, we make no warranty of merchantability or any other warranty, express or implied, with respect to such information, and we assume no liability resulting from its use. Users should make their own investigations to determine the suitability of the information for their particular purposes. In no event shall Adooq bioscience be liable for any claims, losses, or damages of any third party or for lost profits or any special, indirect, incidental, consequential or exemplary damages, howsoever arising, even if Adooq bioscience has been advised of the possibility of such damages.*


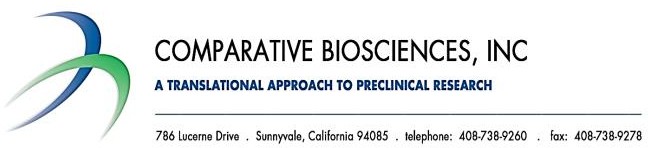


# END of REPORT

Comparative Biosciences, Inc. Study Report

CB21-5006-M-EF Page 63 of 63
