# Supplementary material for: Heat shock protein 90 inhibition attenuates inflammation in models of atopic dermatitis: a novel mechanism of action
Source: Front Immunol. 2024 Jan 11;14:1289788. doi: 10.3389/fimmu.2023.1289788 (PMC10808526; doi:10.3389/fimmu.2023.1289788)
Supplement: Supplementary Figure 1 — Cytotoxicity of RGRN-305 in stimulated primary human keratinocytes. [file DataSheet_2.zip › Figure S1.DOCX]

**
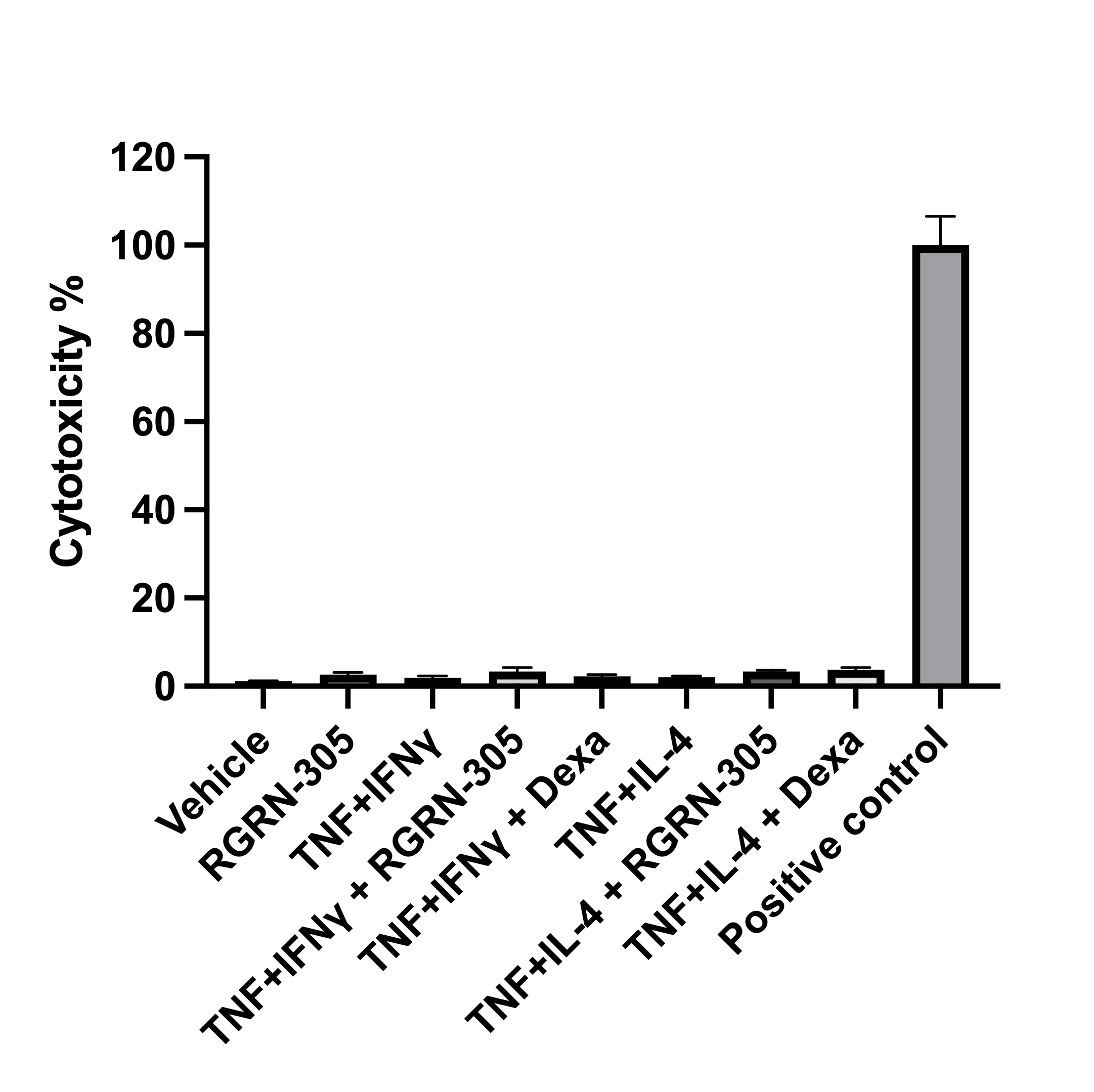
**

**Figure S1 Lactate dehydrogenase cytotoxicity assay in primary human keratinocytes.** The keratinocytes were cultured in 6-well plates and preincubated with 5 µM RGRN-305 or 0.1 µM dexamethasone for 2 hours before stimulation with TNF (10 ng/mL) + IFNγ (10 ng/mL) or TNF (10 ng/mL) + IL-4 (50 ng/mL) for 24 hours. The cytotoxicity was determined by measuring the release of lactate dehydrogenase into the culture media following the manufacturer’s protocol (LDH-Glo^TM^ cytotoxicity assay; Promega, Madison, WI, USA). Fluoroskan Ascent FL (a microplate reader) was used to measure the absorbance at 490 nm. Triton X-100 served as a positive control and was set to 100% cell death.

Abbreviations: LDH, Lactate dehydrogenase. Dexa, dexamethasone
