# Supplementary material for: Heat shock protein 90 inhibition attenuates inflammation in models of atopic dermatitis: a novel mechanism of action
Source: Front Immunol. 2024 Jan 11;14:1289788. doi: 10.3389/fimmu.2023.1289788 (PMC10808526; doi:10.3389/fimmu.2023.1289788)
Supplement: Supplementary Figure 1 — Cytotoxicity of RGRN-305 in stimulated primary human keratinocytes. [file DataSheet_2.zip › Figure S4.DOCX]

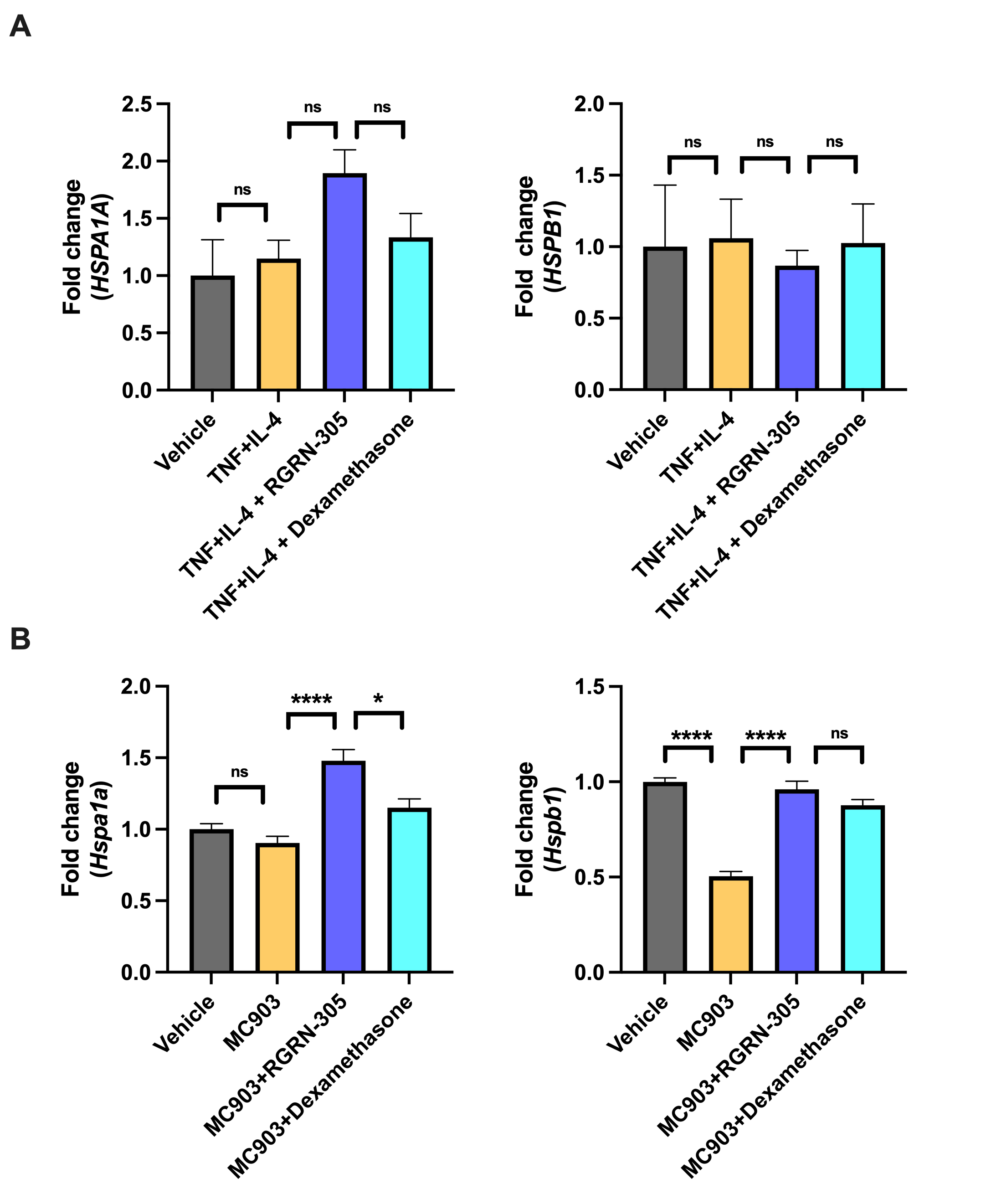


**Figure S4** **Gene expression of *HSPA1A/Hspa1a* and *HSPB1/Hspb1***

**(A)** RT**-**qPCR analysis in primary human keratinocytes. **(B)** RNA sequencing analysis of the MC903-mouse model experiment (mice challenged daily with 1 nmol of MC903 and treated with drug-vehicle, topical RGRN-305 or topical dexamethasone). Data are shown as mean ± SEM. *p < 0.05, ****p ≤ 0.0001. ns, not significant.
