# Supplementary figures and images for: Heat shock protein 90 inhibition attenuates inflammation in models of atopic dermatitis: a novel mechanism of action
Source: Front Immunol. 2024 Jan 11;14:1289788. doi: 10.3389/fimmu.2023.1289788 (PMC10808526; doi:10.3389/fimmu.2023.1289788)

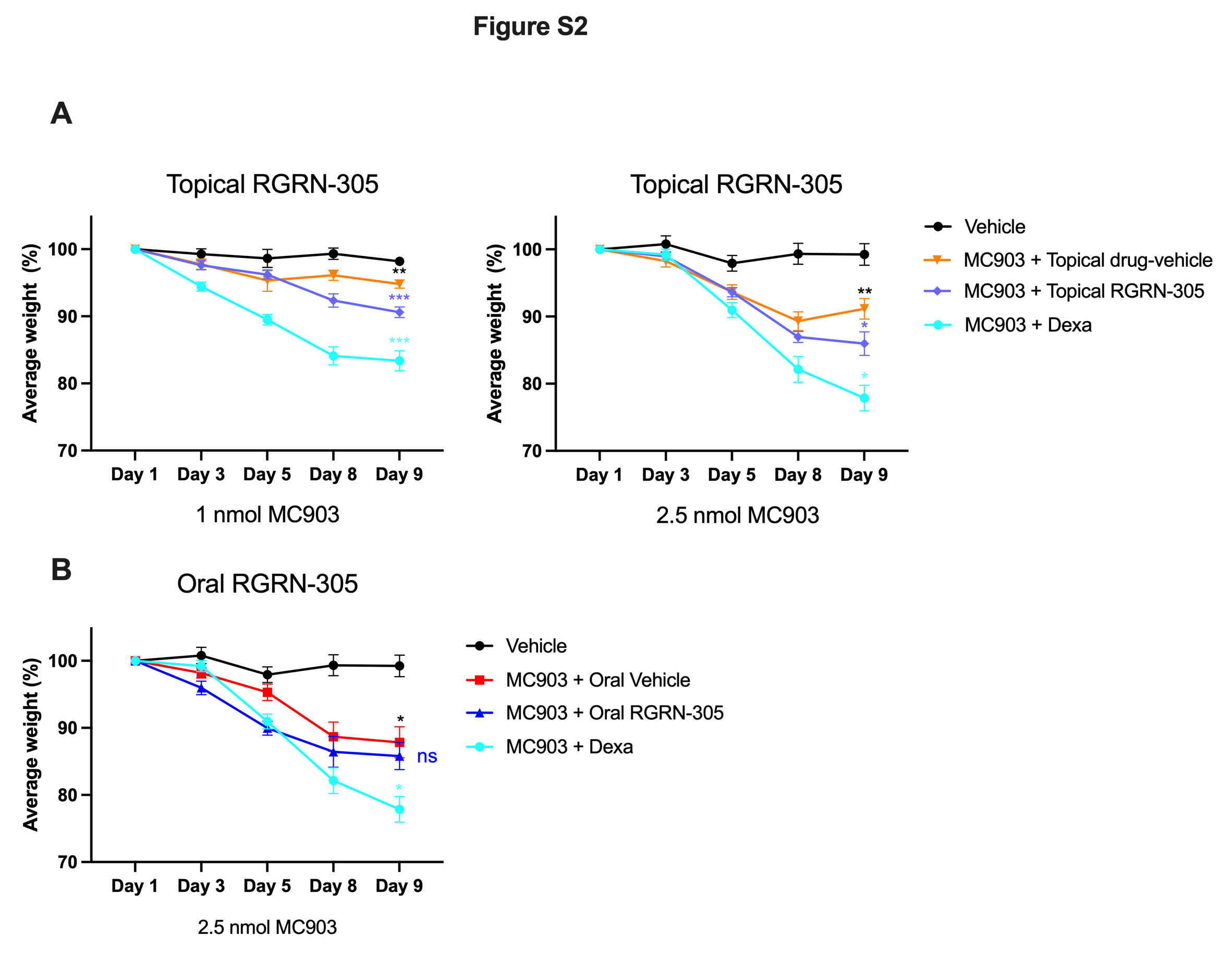

Supplement: Supplementary Figure 1 — Cytotoxicity of RGRN-305 in stimulated primary human keratinocytes. [file DataSheet_2.zip › Figure S2.TIFF]

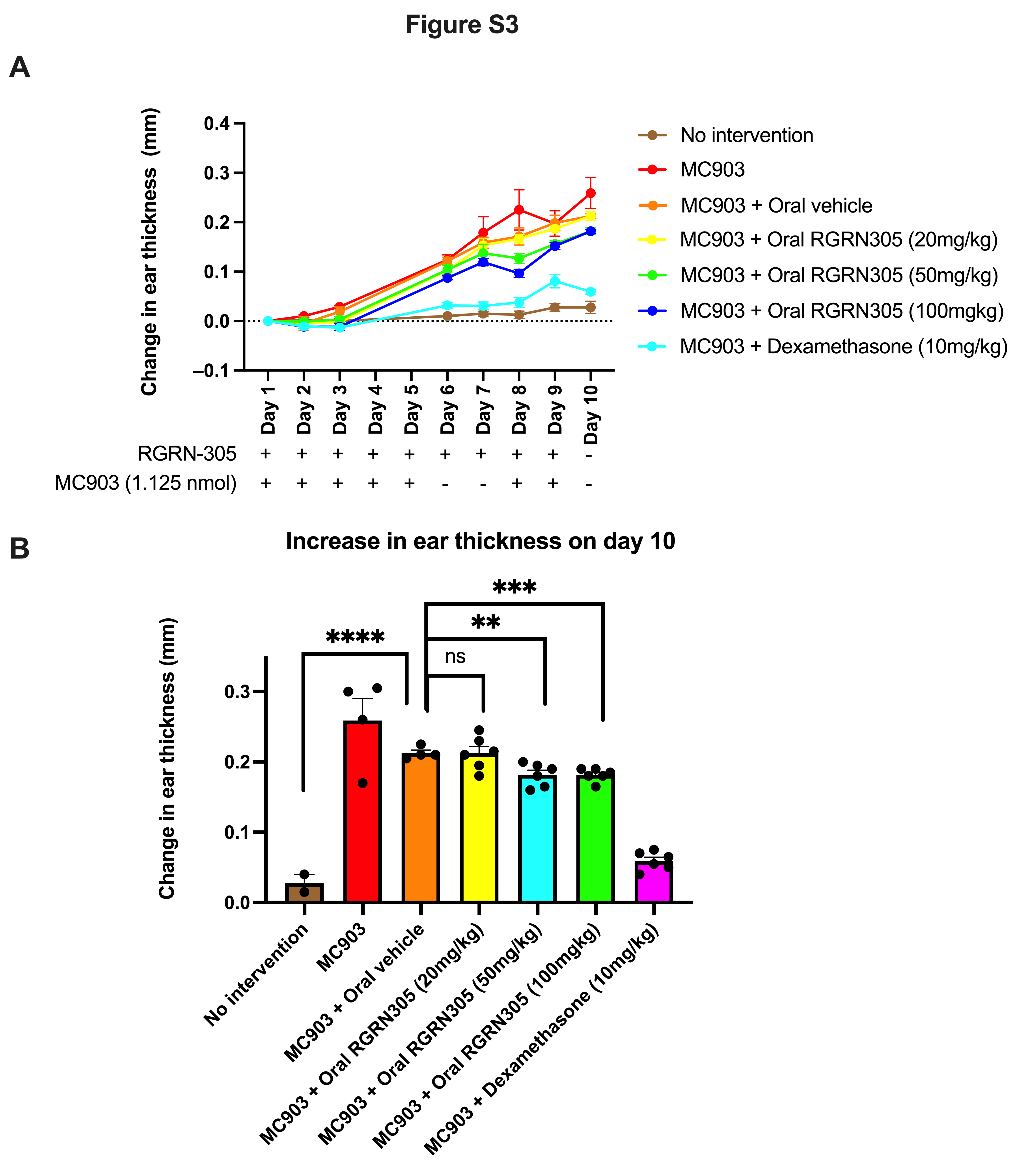

Supplement: Supplementary Figure 1 — Cytotoxicity of RGRN-305 in stimulated primary human keratinocytes. [file DataSheet_2.zip › Figure S3.tiff]
